# Cryo-EM structures of Gid12-bound GID E3 reveal steric blockade as a mechanism inhibiting substrate ubiquitylation

**DOI:** 10.1038/s41467-022-30803-9

**Published:** 2022-06-01

**Authors:** Shuai Qiao, Chia-Wei Lee, Dawafuti Sherpa, Jakub Chrustowicz, Jingdong Cheng, Maximilian Duennebacke, Barbara Steigenberger, Ozge Karayel, Duc Tung Vu, Susanne von Gronau, Matthias Mann, Florian Wilfling, Brenda A. Schulman

**Affiliations:** 1 Department of Molecular Machines and Signaling, Max Planck Institute of Biochemistry, 82152 Martinsried, Germany; 2 Fourth Affiliated Hospital, Zhejiang University School of Medicine, Yiwu, 322000 Zhejiang China; 3 Department of Molecular Structural Biology, Max Planck Institute of Biochemistry, 82152 Martinsried, Germany; 4 Department of Molecular Metabolism, Harvard T.H. Chan School of Public Health, Boston, MA 02115 USA; 5 Institutes of Biomedical Sciences, Shanghai Key Laboratory of Medical Epigenetics, International Co-laboratory of Medical Epigenetics and Metabolism, University of Fudan, 200032 Shanghai, China; 6 Mass Spectrometry Core Facility, Max Planck Institute of Biochemistry, 82152 Martinsried, Germany; 7 Department of Proteomics and Signal Transduction, Max Planck Institute of Biochemistry, 82152 Martinsried, Germany; 8 Mechanisms of Cellular Quality Control, Max Planck Institute of Biophysics, 60438 Frankfurt am Main, Germany

**Keywords:** Ubiquitin ligases, Cryoelectron microscopy, Ubiquitin ligases

## Abstract

Protein degradation, a major eukaryotic response to cellular signals, is subject to numerous layers of regulation. In yeast, the evolutionarily conserved GID E3 ligase mediates glucose-induced degradation of fructose-1,6-bisphosphatase (Fbp1), malate dehydrogenase (Mdh2), and other gluconeogenic enzymes. “GID” is a collection of E3 ligase complexes; a core scaffold, RING-type catalytic core, and a supramolecular assembly module together with interchangeable substrate receptors select targets for ubiquitylation. However, knowledge of additional cellular factors directly regulating GID-type E3s remains rudimentary. Here, we structurally and biochemically characterize Gid12 as a modulator of the GID E3 ligase complex. Our collection of cryo-EM reconstructions shows that Gid12 forms an extensive interface sealing the substrate receptor Gid4 onto the scaffold, and remodeling the degron binding site. Gid12 also sterically blocks a recruited Fbp1 or Mdh2 from the ubiquitylation active sites. Our analysis of the role of Gid12 establishes principles that may more generally underlie E3 ligase regulation.

## Abstract (web summary)

The GID E3 ligase regulates glucose-induced degradation in yeast, and key physiology. This study unveils E3 ligase regulation by reshaping the substrate binding site, blocking substrate access to ubiquitination active sites, and a Cage-like assembly.

## Introduction

A major eukaryotic mechanism controlling the timing of protein expression is ubiquitin-mediated proteolysis. Proteins are selected for degradation by E3 ligases, which recruit specific substrate “degron” motifs and promote ubiquitylation^1^. Ubiquitylation is tightly regulated. In some cases, regulation is achieved by a substrate modification triggering binding to an E3 ligase. In other cases, the E3s are regulated. For example, multiprotein E3 ligases are often activated by timely incorporation of their substrate-binding subunits, or inhibited by factors that block E3 ligase assembly or activity. Despite great progress in understanding how some ubiquitin ligases are controlled, for most E3s, the factors modulating activity, and their mechanisms-of-action remain unknown.

We set out to explore E3 ligase regulatory mechanisms through studies of the multiprotein GID complex. Genetic studies have shown that mammalian, frog, and fly orthologs of the budding yeast *S. cerevisiae* GID E3 (also called “CTLH complex” in higher eukaryotes) are essential for erythropoiesis, organ development and embryogenesis, respectively^2–8^. Moreover, the GID E3 in soybean legumes is critical for rhizobial nodulation required for nitrogen fixation^9^. Molecular functions are best understood for the yeast GID E3, which regulates nutrient-dependent control of carbon metabolism^10–16^. Yeast grown on non-fermentable carbon sources depend on gluconeogenic enzymes to produce glucose. However, when a fermentable carbon source is available, gluconeogenesis is superfluous and terminated through transcriptional and post-transcriptional mechanisms^17,18^. In yeast, the gluconeogenesis enzymes fructose 1,6-biphosphatase (Fbp1), malate dehydrogenase (Mdh2), isocitrate lyase (Icl1) and phosphoenolpyruvate carboxykinase (Pck1) are subject to degradation depending on ubiquitylation by the GID E3 ligase^11,19^. The term GID derives from Fbp1 being ***G***lucose-***i***nduced degradation ***d***eficient in mutants, a majority of which are now recognized to encode subunits (Gid1, Gid2, Gid4, Gid5, Gid7, Gid8, and Gid9) of the multiprotein E3^10–12,19^. Another mutant, *GID3*, encodes the GID E3’s dedicated E2 enzyme partner, Ubc8^20^.

Biochemical and structural studies have shown that the GID E3 is not a singular complex, but a collection of assemblies that vary in activity^21–24^. Some complexes are incompletely assembled, and are inactive due to lack of a substrate-binding receptor subunit. These assemblies are denoted with “Ant” in superscript because they “anticipate” a switch in conditions that triggers expression of a substrate receptor. The core GID^Ant^ complex comprises the Gid1, Gid2, Gid5, Gid8, and Gid9 subunits. A bound substrate receptor is indicated by the nomenclature GID^SR#^, with # referring to its Gid subunit number. The best understood substrate receptor is Gid4, which binds GID^Ant^ to generate GID^SR4^. Gid4 binds a motif, termed Pro/N-degron, defined by a proline at the N-termini of substrates^25,26^.

GID^SR4^ resembles a modular clamp^21^. Cryo-EM structures showed one jaw comprises Gid4, which binds the substrate degron. The center is a Gid5-Gid1-Gid8 scaffold wherein Gid5 binds Gid4, and Gid8 binds Gid9. The other jaw is formed by Gid9 and Gid2, with RING-like and RING motifs, respectively, forming a heterodimeric catalytic domain that activates ubiquitin transfer from Ubc8 to Gid4-bound substrates. GID^SR4^ is sufficient for ubiquitylation and degradation of Mdh2. However, another subunit, Gid7, substantially potentiates ubiquitylation and degradation of Fbp1 by driving supramolecular assembly of two GID^SR4^ E3s into a hollow oval structure termed Chelator-GID^SR4 22^. Fbp1 is encapsulated in the center of the oval, much like a small ligand captured by an organometallic supramolecular chelate^22^.

Although some GID^SR4^-containing assemblies are now understood, several questions about regulation remain unanswered. Gid4 is induced during the switch from gluconeogenic to glycolytic conditions and is essential for ubiquitylation of gluconeogenic enzyme substrates^11^. Although Gid4 levels are controlled by expression and degradation, the regulation is not absolute; low levels of Gid4 are detected in lysates of yeast grown under gluconeogenic conditions^15^. This raises the question of whether there is additional regulation. Further questions relate to assemblies formed in the presence of Gid7. When recombinant Gid7 is added to a GID^SR4^ complex (including two Gid1 and Gid8 subunits), size exclusion chromatography indicated multiple potential higher-order assemblies. Moreover, two human Gid7 orthologs form related but different higher-order assemblies^22,23^, raising the possibility that additional yeast Gid7-driven supramolecular assemblies remain to be discovered.

Here, through studies of Ipf1 (systematic name YDL176W), which we term Gid12, we discover mechanisms modulating GID E3 activity, and a higher-order assembly of three Chelator-GID^SR4^ complexes into a 60-subunit complex that resembles a supramolecular cage (Cage-GID^SR4^) that along with GID^SR4^ and Chelator-GID^SR4^ binds Gid12. The data suggest Gid12 regulates the timing of GID E3 activity by preventing otherwise activated assemblies from engaging in futile ubiquitylation, and may stimulate ubiquitylation of presently unknown substrates. Moreover, the distinctive molecular effects of Gid12 establish principles of E3 ligase regulation.

## Results

### Gid12 binds Gid4-containing GID assemblies

**Fig. 1:**
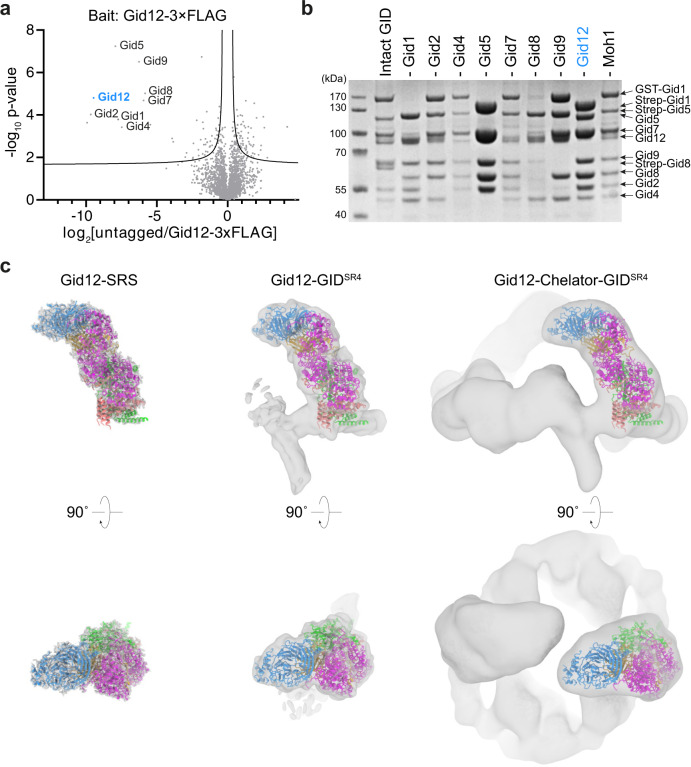
Gid12 binds to the Gid4-containing GID E3 ligase complexes. **a** Quantitative MS identification of proteins interacting with Ipf1-3×FLAG (originally YDL176W, now called Gid12) or an untagged control strain under glycolytic conditions (*n* = 3 biologically independent samples). Data are log-transformed ratios of protein LFQ intensities versus −log10-transformed *P* values of two-tailed Student’s *t* tests. The hyperbolic curve separates specifically interacting proteins from background (square; false-discovery-rate-adjusted *P* = 0.05; minimal fold change S0 = 0.1). The bait protein Gid12 is highlighted in blue. **b** SDS-PAGE of GST- or Strep-affinity purifications after co-infecting insect cells with all GID E3 ligase subunits except the one indicated above the lane. Lane 1 shows the result from a control co-infection with all subunits (*n* = 3 biologically independent experiments). **c** Coordinates of Gid12 bound to the substrate-receptor and scaffolding module (Gid12-SRS) were docked into the cryo-EM density maps of Gid12-SRS, Gid12 bound to GID^SR4^ (Gid12-GID^SR4^) and Chelator-GID^SR4^ (Gid12-Chelator-GID^SR4^) at 3.3, 9.8, and 19.4 Å resolution, respectively, from left to right. Gid12 is colored in blue, Gid4 in gold, Gid5 in magenta, Gid8 in salmon, and Gid1 in green.

Proteomics studies identified Ipf1 in immunoprecipitating with Gid1 and Gid5^16,27^, but the function of this interaction has remained elusive. We confirmed Ipf1 associates with GID E3 ligase complexes using quantitative mass spectrometry (qMS) analysis of immunoprecipitated endogenously C-terminally triple FLAG-tagged Ipf1 (Gid12-3×FLAG) compared to an untagged control strain. Along with Ipf1, all Chelator-GID^SR4^ E3 subunits (Gid1, Gid2, Gid4, Gid5, Gid7, Gid8, and Gid9) were strongly enriched (Fig. 1a; Supplementary Fig. 1a). To maintain terminology for proteins associating with Gid subunits, we use the nomenclature Gid12 rather than Ipf1 or YDL176W.

To enable interrogating Gid12 functions, we sought a recombinant protein expression system. We were unable to generate well-behaved recombinant Gid12 alone. Reasoning that challenges in producing an individual protein can arise from exposure of a hydrophobic surface that is normally buried within a multiprotein complex, we tested if Gid12 could be produced upon co-expression with interaction partners. We generated individual baculoviruses for Gid12, all Gid subunits, and Moh1 (the yeast ortholog of human protein YPEL5, which binds the human Gid7 ortholog WDR26^22,24^), some with GST or Strep affinity tags. Affinity purifications from lysates of insect cells infected with various combinations tested roles of each subunit in binding Gid12 and led to three key conclusions (Fig. 1b): (i) Co-expression of all subunits, or all subunits except Gid7 showed that Gid12 associates with both Chelator-GID^SR4^ and GID^SR4^; (ii) Gid12 can form a subcomplex with the substrate receptor (Gid4) and its interacting protein in the scaffold (Gid5); and (iii) Gid12 binding was reduced upon exclusion of Gid4. Taken together, the data with recombinant GID assemblies suggest that Gid12 associates with complexes containing Gid4. Furthermore, qMS analysis of the endogenous yeast GID E3, immunoprecipitated based on a core subunit (Gid8 endogenously C-terminally 3×HA-tagged), showed Gid4 plays a crucial role for Gid12 association with the GID E3 in vivo (Supplementary Fig. 1b).

### Cryo-EM structure shows Gid12 projects from the scaffold to surround Gid4

To gain insights into potential functions of Gid12, we obtained cryo-EM reconstructions for Gid12 bound to the substrate-receptor and scaffolding (SRS) module (a Gid12-Gid4-Gid5-Gid1-Gid8 complex, referred to as Gid12-SRS), to GID^SR4^ (referred to as Gid12-GID^SR4^), and to Chelator-GID^SR4^ (referred to as Gid12-Chelator-GID^SR4^) at 3.3, 9.8, and 19.4 Å resolution, respectively (Fig. 1c; Supplementary Figs. 1c–h, 2; Supplementary Tables 2, 3). Docking of prior GID subcomplex structures into the maps showed Gid4 covered by a prominent dome-shaped density corresponding to Gid12 (Supplementary Fig. 3a). Thus, we built coordinates for Gid12-SRS, and generated atomic models of Gid12-GID^SR4^ and Gid12-Chelator-GID^SR4^ by docking them with Gid12-SRS and the respective prior structures of GID^SR4^ and Chelator-GID^SR4^ (Fig. 1c; Supplementary Fig. 3b, c; Supplementary Table 4).

**Fig. 2:**
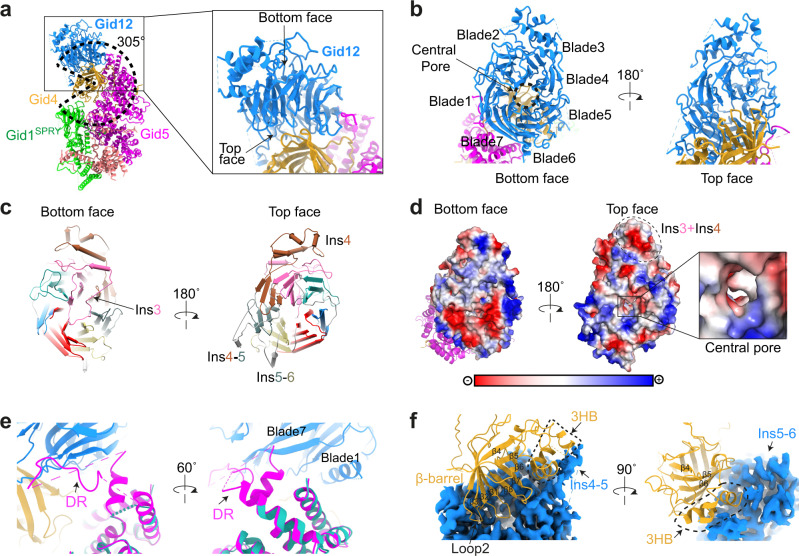
Gid12 binds both the substrate receptor Gid4 and the tip of Gid5 in the scaffolding module. **a** Overall structure of Gid12-SRS. Gid12 is colored in blue, Gid4 in gold, Gid5 in magenta, Gid8 in salmon, and Gid1 in green. Dotted wedge highlights that Gid12 projects from the scaffolding module such that the substrate receptor, Gid4, is enwrapped around 305° in the complex. Closeup highlights interactions between Gid12, Gid4, and Gid5. **b** Gid12 forms a 7-bladed β-propeller, with its top face and central pore extensively interacting with Gid4, and edge binding Gid5. The β-propeller blades are labelled 1–7. **c** Gid12 structural elements, showing blades 1–7 from bottom and top faces of the β-propeller, color-coded in blue, green, hot pink, burnt orange, slate gray, khaki, and red, respectively. A meandering insertion in the 3rd blade (Ins3), indicated by the arrow, plugs the central pore from the bottom face of β-propeller (left); Insertion between the 3rd and 4th β-sheets in blade 4 (Ins4), insertion between blades 4 and 5 (Ins4-5), and insertion between blades 5 and 6 (Ins5-6) project outward from the center of the β-propeller (right). **d** Gid12 surface representation, colored by electrostatic surface potential. The bottom face is highly charged (red), whereas the top face, the surface of Ins3-Ins4 5-helix bundle, and central pore (see closeup), which interact with Gid4, are substantially hydrophobic (white). **e** Closeups of the superposition of Gid12-SRS with a prior structure without Gid12 (cyan, PDB ID: 6SWY) show that a ≈30-residue Gid5 disordered region (DR)—invisible in the published cryo-EM maps without Gid12—binds an edge of Gid12 formed by blades 1 and 7. **f** Closeups showing extensive interactions between Gid12, shown as blue cryo-EM density, and Gid4 shown as gold ribbon. The Gid4 β-barrel interacts with the top face of Gid12. The Gid4 3-helix bundle (3HB) interacts with Ins4-5 and Ins5-6 from Gid12.

In agreement with the biochemical data, Gid12 primarily contacts Gid4, and also Gid5. Prior structures showed that Gid4 contains a cylindrical 8-stranded β-barrel domain that engages a substrate’s Pro/N-degron in a central tunnel^22,26^, while the exterior of the cylinder nestles in a semicircular groove formed by the SPRY domain of Gid1 and the C-terminal half of Gid5’s armadillo repeats^21^. Gid12 emanates from and extends this groove, to wrap ≈305° around Gid4. In essence, Gid12 seals Gid4 onto the scaffolding module (Fig. 2a).

Gid12 forms a seven-bladed β-propeller (Fig. 2b). β-propellers are characterized by bottom and top faces and a central pore (Fig. 2a, b). The Gid12 propeller is embellished by sequences inserted between strands within blades. Three insertions (Ins4, Ins4-5 and Ins5-6) form helical structures that pack either against the sides of the propeller or between blades, while an insertion in the third blade of Gid12 (Ins3) forms a meandering loop that plugs the central pore on the bottom face of the propeller (Fig. 2c). Although the bottom face of Gid12 is charged, the top face of the propeller, its entrance to the central pore, and exposed surfaces from a helical embellishment (Ins3 + Ins4) are all exceptionally hydrophobic (Fig. 2d). The extensive hydrophobic surface presumably accounts for the challenges in producing Gid12 alone. However, much of this hydrophobic surface is buried through intricate interactions with Gid4 (Fig. 2a, b).

Gid12’s interactions with Gid4 and Gid5 bury more than 2400 Å^2^ of surface area. The Gid12-Gid5 interaction primarily involves the edges of blades 1 and 7 on the side of the Gid12 propeller binding a ≈ 30-residue Gid5 region that was disordered in prior structures and is presumably stabilized in the complex (Fig. 2e).

More than 60 residues from Gid12 interact with more than 40 residues from Gid4. Gid4 contacts loops within every blade—and every loop between blades—on the top face of the Gid12 β-propeller, and two insertions. Gid12 interacts across a diagonal around all eight strands in the Gid4 β-barrel, from the N-terminal tip of strand 4 at one edge of the cylinder, nearly the entire length of strands 1 and 8 at the center of the interaction, to the N-terminal tip of strand 5 at the other edge of the cylinder. Also, insertions between Gid12 blades 4 and 5 (Ins4-5) and blades 5 and 6 (Ins5-6) bind a 3-helix bundle on one side of the Gid4 β-barrel (Fig. 2f).

### Gid12-binding remodels the substrate-binding site

**Fig. 3:**
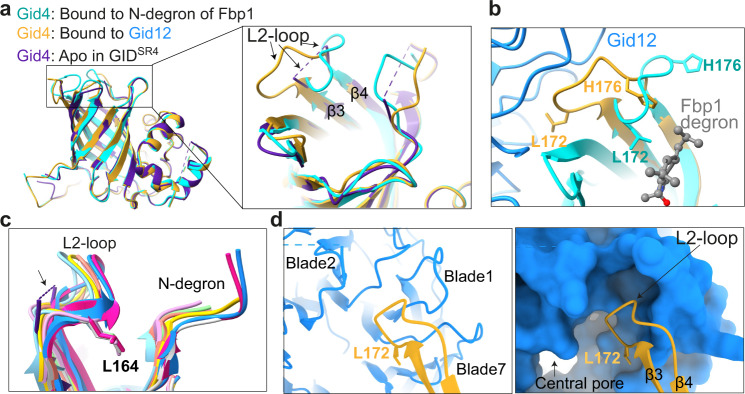
Gid12 remodels the N-degron binding pocket in Gid4. **a** Superposition of Gid12-bound Gid4 (gold) with prior structures of Gid4 from GID^SR4^ complexes without substrate (“Apo”, PDB ID: 6SWY, purple) and bound to the Pro/N-degron of Fbp1 (PDB ID: 7NS3, cyan) reveals conformational heterogeneity of Gid4 L2-loop. **b** Closeup showing structural differences in Gid4 L2-loop when bound to Gid12 (Gid4 in gold) or to Pro/N-degron of Fbp1 substrate (Gid4 in cyan, PDB ID: 7NS3). **c** L2-loop structural similarity amongst human Gid4 structures with various N-degron peptides (PDB ID: 6CCT, 6CCU, 6CD8, 6CD9, 6CDC, 6CDG, 6WZX, 6WZZ; colored in cyan, pink, lawn green, salmon, gray, yellow, deep pink and blue respectively). Yeast Gid4 from apo GID^SR4^ (6SWY) is shown in purple with its disordered L2-loop indicated with arrow and dotted line, and human Gid4 Leu164, which corresponds to yeast Gid4 Leu172, are shown for reference. **d** Closeups showing Gid4 L2-loop (gold) bound to Gid12 (blue), with Gid12 depicted as ribbon (left) or surface (right). The Gid4 L2-loop conformation complements the Gid12 pocket at the junction between blades 7 and 1 in the central pore.

Although at an overall level, Gid12-bound Gid4 superimposes with prior structures without Gid12 (0.47 Å RMSD, 6SWY.PDB; and 0.56 Å RMSD, 7NS3.PDB) (Fig. 3a), these structures themselves show variability in the conformations of loops at the entrance to the substrate-binding tunnel^21,22^. Among the differences, Gid4’s L2-loop was not visible in the cryo-EM density for GID^SR4^ in the absence of substrate, potentially reflecting intrinsic conformational flexibility (Fig. 3a). However, in the structure of Chelator-GID^SR4^ bound to Fbp1, Gid4’s L2-loop clamps around the peptide-like N-terminus of an Fbp1 protomer inserted into the substrate-binding tunnel, and the loop residue Leu172 contacts the Pro/N-degron (Fig. 3b). Moreover, the position of the L2-loop and surrounding side-chains vary amongst crystal structures of human Gid4 bound to different peptides; the Gid4 loop structures conform to diverse peptide sequences, although they all show Leu164 (corresponding to yeast Gid4 Leu172) facing into the substrate-binding tunnel and contacting a bound peptide^26,28,29^ (Fig. 3c).

The structure of Gid4’s L2-loop is strikingly rearranged in the complex with Gid12 (Fig. 3a). Gid12 extracts Gid4’s L2-loop, such that Leu172 and surrounding residues faces outside the central cylinder, and bind a pocket at the junction between Gid12’s blades 7 and 1 (Fig. 3d). This L2-loop remodeling results in relocation of His176 from the periphery to inside the substrate-binding tunnel, where it approaches the position normally occupied by Leu172 (Fig. 3b).

### Cryo-EM maps suggest Gid12 would occlude substrate encapsulation by Chelator-GID^SR4^

**Fig. 4:**
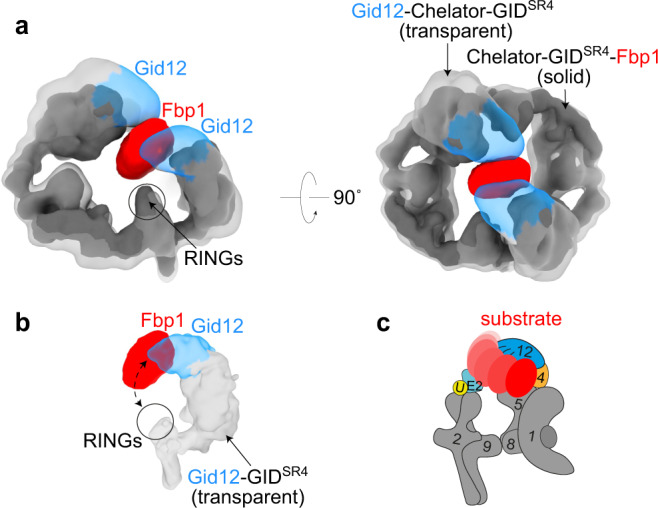
Gid12 fully and partially obstructs substrate placement in Chelator-GID^SR4^ and GID^SR4^ assemblies, respectively. **a** Superposition of 3D reconstructions of Gid12-Chelator-GID^SR4^ (transparent gray, with Gid12 in blue) and Chelator-GID^SR4^-Fbp1 (solid gray, with substrate Fbp1 in red) shows that Gid12 fills much of the central region of Chelator-GID^SR4^ that encapsulates the globular domain of the recruited Fbp1 substrate. **b** Superposition of 3D reconstruction of Gid12-GID^SR4^ with the corresponding region of the map of Chelator-GID^SR4^-Fbp1. The relatively open structure of GID^SR4^ does not encapsulate Fbp1, whose globular domain may thus occupy various relative positions between the Pro/N-degron recruited to Gid4 and RING-based catalytic module. **c** Cartoon representation showing how GID^SR4^ could dynamically accommodate various orientations of Fbp1 between Gid4 and RING-activated Ubc8~ubiquitin intermediate, some of which could be compatible with Gid12 bound to Gid4.

Gid12 projects over the substrate-binding site in Gid4 toward the heterodimeric catalytic domain consisting of the Gid2 RING and Gid9 RING-like motifs in GID^SR4^ and Chelator-GID^SR4^. These catalytic domains are unencumbered by Gid12 (Fig. 4a, b), suggesting that Gid12 would not impact the RING-catalyzed chemical reaction of discharging ubiquitin from a recruited E2 enzyme. However, Gid12, rigidly affixed to Gid4 and Gid5, would partially occupy the space between Gid4 and the RING domains and could limit substrate accessibility of a bulky substrate to a ubiquitylation active site. This is most striking for the Chelator assembly, where Gid12 fills much of the region previously shown to encapsulate the centrally-recruited Fbp1 substrate^22^ (Fig. 4a). However, the relatively open structure of GID^SR4^ may in principle accommodate some orientations of protein substrates between Gid4 and a RING-activated Ubc8~ubiquitin intermediate (Fig. 4b, c).

### A ≈ 5 MDa 60-subunit Cage-GID^SR4^ assembly with or without Gid12

**Fig. 5:**
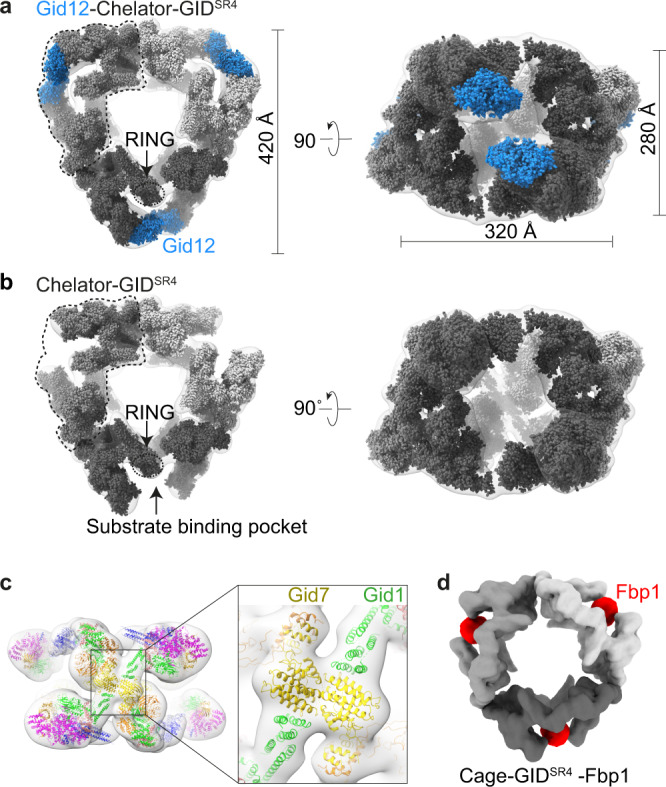
Cryo-EM maps of a ≈ 5 MDa 60-subunit Cage-GID^SR4^ assembly. **a** Overall architecture of Gid12-Cage-GID^SR4^ is seen from its transparent cryo-EM density fit with three copies of models of Gid12-Chelator-GID^SR4^ in solid surfaces in different shades of gray, with models for Gid12 (this study) in blue. **b** 19.8 Å-resolution cryo-EM density for Cage-GID^SR4^ is shown as a transparent surface fit with three copies of models of Chelator-GID^SR4^ (EMD-12541) in solid surfaces in different shades of gray. **c** Models of Gid1 and Gid7 domains fitted at junctions of the three individual Chelator-GIDs. These interaction at the junction constitute the cage architecture. The resolution of the density, together with prior maps and models, allows attributing domains but not their specific orientations or interactions. **d** 12 Å-resolution cryo-EM density map of Cage-GID^SR4^-Fbp1 (here, the N-terminal degron of Fbp1 - PTLVNG was exchanged with Mdh2 degron - PHSVTP to increase stability). Fbp1 tetramer encapsulated in the oval center of each Chelator-GID^SR4^ is color-coded in red.

Unexpectedly, the cryo-EM data for the Gid12-bound complex prepared with Gid7 also revealed an even larger assembly with dimensions of 420 Å × 320 Å × 280 Å (Fig. 5a; Supplementary Fig. 2d–f; Supplementary Tables 2, 3). A 20.6 Å resolution reconstruction was readily fit with three copies of Gid12-Chelator-GID^SR4^. Cryo-EM data for a complex prepared without Gid12 also showed the same arrangement of three Chelator-GID^SR4^ assemblies (Fig. 5b; Supplementary Fig. 4; Supplementary Tables 2, 3). Composite models showed 66- or 60-protein ≈5 MDa complexes comprising 12 Gid1:6 Gid2:6 Gid4:6 Gid5:12 Gid7:12 Gid8:6 Gid9 subunits, with or without 6 protomers of Gid12, respectively (Supplementary Fig. 5a, b). We refer to this assembly as Cage-GID^SR4^ (or Gid12-Cage-GID^SR4^) due to its overall structure comprising three oval-shaped Chelator-GID^SR4^ assemblies fused in a cage-like arrangement. Reevaluation of cryo-EM data obtained for GID purified from yeast^22^ confirmed that the endogenous complex also forms a Cage assembly (Supplementary Fig. 6a–c; Supplementary Tables 2, 3). For perspective, Cage-GID is roughly twice the molecular weight of a doubly-capped 26 S proteasome.

The maps show that the cage is formed through three total junctions between the constituent ovals, one between each pair of Chelator-GID^SR4^ assemblies (Fig. 5c). The oval Chelator-GID^SR4^ shape arises through daisy chain-like interactions between double-sided LisH-CTLH-CRA domains in Gid1, Gid8, Gid2, Gid9, and Gid7. The side containing CTLH helices and the N-terminal CRA helices is known as “CTLH-CRA^N^”^22^. The composite model of Cage-GID^SR4^ shows that the junctions responsible for cage formation correspond to CTLH-CRA^N^ domains of the Gid7 protomers that connect to CTLH-CRA^N^ domains of Gid1 in the substrate receptor scaffolding modules (Fig. 5c). These domains were poorly resolved in the prior cryo-EM reconstructions of Chelator-GID^SR4 22^. Nonetheless, they can be roughly approximated based on homologous but asymmetric interactions between CTLH-CRA^N^ domains of other non-equivalent Gid7 and Gid1 protomers in Chelator-GID^SR4^ (Supplementary Fig. 5c).

To date, Fbp1 is the only GID E3 substrate known to require Gid7 for glucose-induced degradation. A 12.2 Å resolution Fbp1-bound Cage-GID^SR4^ (Cage-GID^SR4^-Fbp1) showed Fbp1 encapsulated within the centers of each constituent Chelator as in the prior structure of Fbp1-bound Chelator-GID^SR4 22^ (Fig. 5d; Supplementary Fig. 6d–f; Supplementary Tables 2, 3). Thus, the cage would be expected to retain the E3 ligase features of Chelator-GID^SR4^ including Gid12 blockage of the central space occupied by protein substrates. However, at this point, how, when, or why this Cage-GID^SR4^ assembly forms remain unknown. Future studies will be required to determine biological roles specific to Cage-GID^SR4^.

### Impact of Gid12 on biochemically reconstituted GID^SR4^ E3 assembly and substrate ubiquitylation

**Fig. 6:**
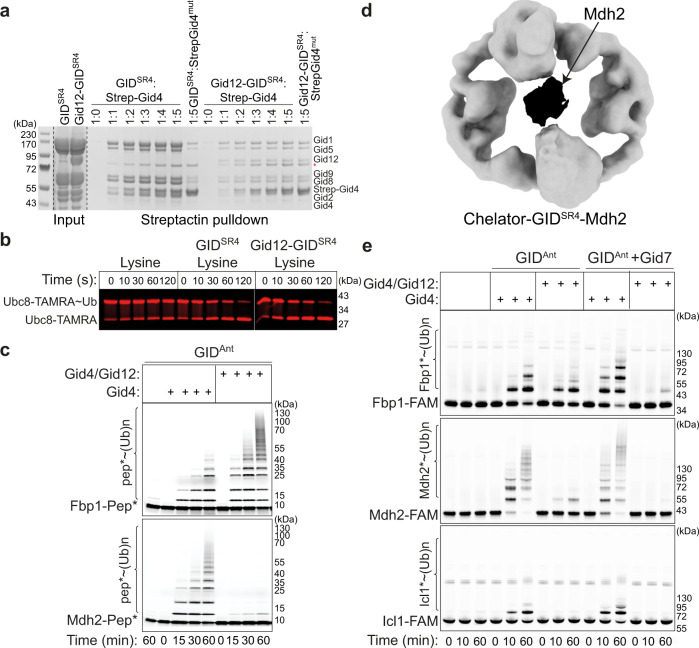
Gid12 biochemically modulates both assembly of Gid4 into the GID E3 ligase complex and its activity toward its substrates. **a** Strep-tagged Gid4 in solution can replace untagged Gid4 within GID^SR4^ but not in the Gid12-bound complex. A mutant (mut) lacking the C-terminal four residues 359–362 required for incorporation into GID^SR4^ is used as a control for specificity of Strep-Gid4 incorporation into the complex (*n* = 3 biologically independent experiments). **b** Pulse chase assay examining effects of Gid12 on GID ubiquitin transferase activity in substrate-independent manner. Ubc8 is C-terminally fluorescently labeled with TAMRA for fluorescence detection of the free E2 versus the version thioester-linked to Ub. First, the thioester-linked Ubc8-TAMRA~Ub intermediate was generated in a pulse reaction. Second, the chase reaction monitors formation of the faster-migrating Ubc8-TAMRA upon Ub discharge to free lysine, stimulated by GID^SR4^ with or without a bound Gid12 (*n* = 3 biologically independent experiments). **c** In vitro ubiquitylation assay of model peptide substrates (with an N-terminal substrate degron, a flexible linker, a single lysine at optimal position to accept ubiquitin, and a C-terminal fluorescein for fluorescent detection) testing effects of adding purified Gid4 alone or Gid4 co-expressed with Gid12 to GID^Ant^ (*n* = 3 biologically independent experiments). **d** 18.9 Å-resolution cryo-EM density map of Chelator-GID^SR4^-Mdh2. Mdh2 dimer encapsulated in the oval center of Chelator-GID^SR4^ is color-coded in black. **e** Experiment as in (**c**), except with the indicated full-length metabolic enzyme substrates, each appended to a C-terminal fluorescein for fluorescent detection (*n* = 3 biologically independent experiments).

Given that the structures show Gid12 sealing Gid4 onto the scaffold, it seemed likely that Gid12 would reduce Gid4 dissociation from the GID^SR4^ complex. To test this hypothesis, we devised a competitive binding assay. Briefly, Gid4 dissociation from GID^SR4^ would produce GID^Ant^, which could then assemble with free Gid4 in solution^11,21^. We mixed GID^SR4^ or the Gid12-GID^SR4^ - both containing untagged Gid4 - with excess Strep-tagged Gid4 in solution. Performing Strep-Tactin pulldowns showed Strep-tagged Gid4 provided in solution could readily replace the untagged version in the GID^SR4^ complex. Importantly, a negative control mutant version of Strep-Gid4 (lacking C-terminal residues required for binding Gid5 in the scaffold module) failed to associate with any GID^SR4^ or Gid12-bound GID^SR4^ subunits. In agreement with the structural data, Gid12 greatly reduced the capacity of the Gid4 subunit within the GID^SR4^ complex to be replaced by even fivefold molar excess of Strep-tagged Gid4 (Fig. 6a). Thus, Gid12 reduces the dissociation of Gid4 from within a GID^SR4^ E3 complex.

Several features displayed in the cryo-EM structures suggest aspects of E3 ligase activity that would or would not be affected by Gid12. First, Gid12 is distal from the catalytic domains, which suggests that it would not impact substrate-independent E3 ligase activity. Indeed, GID^SR4^-dependent discharge of ubiquitin to the amino acid acceptor lysine was unaffected by Gid12 (Fig. 6b).

Second, the remodeled substrate-binding tunnel in Gid12-bound Gid4 could affect recognition of a substrate’s degron. Although neither we nor others^26,28^ have been able to generate sufficiently high concentrations of well-behaved yeast Gid4—either alone or in complex with other GID E3 subunits—to quantify degron binding, we could test effects on ubiquitylation of fluorescently-labeled model substrates wherein a lysine is flexibly tethered to substrate’s Pro/N-degron sequence. Prior studies showed differences amongst substrate degrons: the Mdh2 degron motif is sufficient to confer robust GID^SR4^-mediated polyubiquitylation of a model peptide substrate, whereas a peptide harboring the weaker Fbp1 degron is ubiquitylated to a lesser extent^22^. For the Fbp1 protein, tetramerization overcomes the weak degron of an individual protomer by enabling avid binding to higher-order GID^SR4^ assemblies (Chelator or presumably Cage)^22^. Interestingly, Gid12 swaps intrinsic preference of GID^SR4^ toward the different degrons, inhibiting ubiquitylation of the peptide with the Mdh2 degron, but potentiating ubiquitylation of that with the Fbp1 degron (Fig. 6c).

Third, the structural location of Gid12 would obstruct a Gid4-bound substrate from accessing the ubiquitylation active site of the Ubc8~ubiquitin intermediate bound to the Gid2-Gid9 catalytic module^21,22,25,26,30,31^. This is even more striking for a Chelator (or Cage) assembly, wherein Gid12 blocks the central space shown previously to encapsulate Fbp1, and which we find also encapsulates Mdh2 (Fig. 6d; Supplementary Fig. 7; Supplementary Tables 2, 3). Thus, we performed in vitro ubiquitylation experiments to test impact of Gid12 on various protein substrates by GID^SR4^ and Chelator/Cage-GID^SR4^. As described previously, adding Gid4 to GID^Ant^ enables substrate binding and thus ubiquitylation, which is potentiated upon Gid7-mediated higher-order GID E3 assembly^21,22^. To test effects of Gid12, we purified Gid4 bound to Gid12, and compared effects of adding this complex side-by-side with Gid4 alone. Ubiquitylation of gluconeogenic enzyme protein substrates diminished upon addition of Gid12. In accordance with the structural models, the suppressive effect of Gid12 was particularly potent toward the substrate Fbp1 in the reactions including Gid7 (Fig. 6e). Thus, even if an Fbp1 degron encounters a Gid12-bound GID E3, its ubiquitylation would be sterically hindered.

### Gid12 overexpression stabilizes Fbp1 protein in vivo

**Fig. 7:**
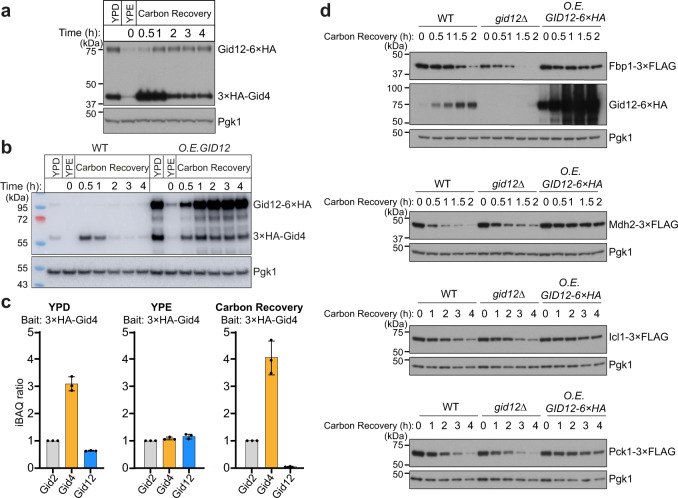
Overexpression of Gid12 stabilizes Fbp1 in vivo. **a** Expression profiles of Gid4 and Gid12 tagged at their endogenous loci, under different growth conditions, monitored by western blotting against hemagglutinin tag (HA). YPD: glycolytic conditions; YPE: overnight non-fermentable carbon source starvation; Carbon Recovery: glucose replenished with YPD. Pgk1 served as loading control (*n* = 3 biologically independent experiments). **b** Degradation of 3×HA chromosomally tagged Gid4 in wildtype (WT) and Gid12-overexpressing (*GPD* promoter) cells were monitored using anti-HA immunoblotting. Gid4 was stable under carbon recovery conditions in a strain continuously overexpressing Gid12 (*n* = 3 biologically independent experiments). **c** Quantitative mass spectrometry analysis of the relative levels of Gid2, Gid4 and Gid12 in anti-HA immunoprecipitates of lysates expressing Gid4 with an N-terminal 3×HA tag at the endogenous loci. Yeast cells were grown under glycolytic (YPD), non-fermentable carbon source starvation (YPE), or carbon recovery conditions. Data are mean ± s.d. of *n* = 3 biologically independent experiments. iBAQ ratios are formed using the respective Gid2 iBAQ value as denominator. **d** Monitoring degradation of 3×FLAG chromosomally tagged gluconeogenic enzymes in wildtype (WT), Gid12-deficient (*gid12∆*), and Gid12-overexpressing (*ADH1* promoter) cells using anti-FLAG immunoblotting. The effects of *GID12* deletion to gluconeogenic enzymes are subtle, however, all four gluconeogenic enzymes are stabilized under carbon recovery conditions in a strain consecutively overexpressing Gid12 (*n* = 3 biologically independent experiments).

As first steps toward addressing functional roles of Gid12, we asked how its protein abundance changes across different metabolic conditions, and how this compares to the pattern of Gid4 expression. Appending six copies of the HA-tag to the C-terminus of Gid12 at the endogenous locus was required to robustly detect Gid12, which was previously estimated by quantitative total proteomics to be present at only 35 molecules of Gid12 per yeast cell in the S288C background^32^. We also appended our standard triple HA-tag to Gid4. The tagging scheme allowed simultaneous detection by immunoblotting lysates from yeast harvested across a time course from gluconeogenic to glycolytic growth conditions. Expression of both proteins was strongly suppressed during growth on a non-fermentable carbon source, and recovered following glucose replenishment. However, their relative expression patterns diverged upon the metabolic switch. As expected, there was a surge in Gid4 expression within the first hour after glucose was restored to carbon-starved yeast, followed by progressive decrease over time. In contrast, the relative levels of Gid12 increased at later time-points when Gid4 levels reach steady-state in glucose-rich media (Fig. 7a). Notably, repeating the experiment side-by-side with *GID12* overexpression (and detecting with decreased exposure time) showed that elevated Gid12 resulted in increased Gid4 levels at later time-points after switching from gluconeogenic to glycolytic conditions (Fig. 7b).

We next probed the relative abundance of endogenous Gid12 in complex with Gid4 by co-immunoprecipitating with endogenously tagged Gid4 (3×HA-Gid4) in different metabolic conditions. Relative levels of Gid4 and Gid12 were quantified by intensity-based absolute quantification (iBAQ^33^). The data correlated well with the relative levels observed by western blot (Fig. 7a) in that under ongoing glycolytic growth conditions, the ratio of Gid12 in a Gid4 immunoprecipitation was ~1:5. Even though the level of Gid4 decreased under gluconeogenic growth conditions as shown by western blot, the Gid4 that was present during gluconeogenesis co-immunoprecipitated Gid12 with nearly 1:1 stoichiometry (Fig. 7c; Supplementary Fig. 8a; Supplementary Table 5).

We monitored the levels of well-characterized Gid4-dependent substrates—Fbp1, Mdh2, Icl1, and Pck1 C-terminally 3×FLAG-tagged at their endogenous loci to allow detection by immunoblotting—upon glucose replenishment after growth in ethanol in strains in which *GID12* was either deleted or overexpressed. Although the effects of *GID12* deletion were subtle, all four endogenous substrates were stabilized upon *GID12* overexpression, in agreement with the in vitro data (Fig. 7d). Thus, despite the normally low Gid12 levels during the switch from gluconeogenesis to glycolysis, its overexpression blocks glucose-induced substrate degradation. To gain broader perspective, we performed quantitative proteomics and examined protein levels of known GID E3 substrates^10,34,35^. As expected, statistically significant increases in Gid4-dependent substrates (Fbp1, Mdh2, Icl1, and Pck1) were observed at various time-points after the switch from gluconeogenic to glycolytic growth conditions upon overexpression of *GID12* (Supplementary Figs. 8b, 9; Supplementary Tables 7–9). In addition to Gid4, recent studies have discovered two additional substrate receptors, Gid10 and Gid11, mediating distinct regulation^21,34,35^. The current working model is that the substrate receptors bind interchangeably, and mutually-exclusively, to GID^Ant^ under different environmental conditions. Gid10 was not detected in the proteomics experiments, while Gid11 was detected only in the set of experiments testing effects of deleting *GID12* (Supplementary Fig. 9; Supplementary Tables 7–9). Accordingly, the substrates attributed to recognition by Gid10 or Gid11 were not overtly impacted by overexpression of *GID12* (Supplementary Figs. 8b, 9; Supplementary Tables 7–9). Gid11 is predicted to form a unique β-propeller structure and has been recalcitrant to recombinant protein production, while the sequence and structure of Gid10 is substantially homologous to Gid4 (Supplementary Fig. 10a, b). Accordingly, Gid10 recognizes Pro/N-degron sequences much like Gid4^29,34,36^. Indeed, Gid12 can form a complex with GID^SR10^ when the subunits are co-expressed in insect cells (Supplementary Fig. 10c), which is consistent with the conservation of Gid12-binding residues of Gid4 in Gid10. Future studies will be required to determine if Gid12 regulates other complexes other than those containing Gid4—particularly those with Gid10—in different environmental settings.

Interestingly, the level of Aro10 also increased upon *GID12* overexpression (Supplementary Figs. 8b, 9; Supplementary Tables 7–9). Aro10 was previously identified as a candidate Pro/N-degron GID E3 substrate based on its stabilization upon mutation of catalytic subunits of the GID complex^35,37^, mutation of the Pro in position 3 in its encoded sequence^38^, and of N-terminal aminopeptidases that remove N-terminal residues upstream of a Pro^38^. Although to date Aro10 has not been found to be stabilized upon deletion of any known GID E3 ligase substrate receptors^35,37^, our data reveal a correlation with Gid4-dependent regulation, and raise the possibility of yet additional forms of GID E3 ligase regulation to be discovered in future studies.

## Discussion

Here, we show that both in vivo and in vitro Gid12 binds directly to the GID E3 ligase assemblies harboring the substrate receptor Gid4 (Fig.1; Supplementary Fig. 1b). Our collection of cryo-EM reconstructions shows that Gid12 forms an extensive interface with Gid4 involving a total of more than 100 residues, sprawling beyond Gid4 to form a junction with Gid5 (Fig. 2a). Notably, Gid12 also seems to capture a relatively dynamic region of Gid5 that was not well resolved in EM maps in the absence of Gid12 (Fig. 2e). The structural data served to guide biochemical studies with purified and defined components, and of regulation of gluconeogenic enzyme substrates of the GID E3 in vivo.

Gid12 influences GID E3 structure and function through multiple mechanisms. First, Gid12-interacting regions of Gid4 include flexible loops at the entrance to the substrate-binding tunnel (Fig. 3). As a result, the degron binding site is reshaped such that ubiquitylation of a peptide substrate bearing the Fbp1 Pro/N-degron is increased, but ubiquitylation of the peptide bearing the Mdh2 Pro/N-degron is inhibited (Fig. 6c). Notably, Gid12 does not impact GID^SR4^ E3-dependent ubiquitin discharge to lysine (Fig. 6b). Although we are unable to directly quantify degron peptide binding, the results from ubiquitylation assays are consistent with Gid12 modulating degron binding, and at minimum excluding some substrates (e.g., Mdh2) and allowing —and likely improving—intrinsic ability of GID^SR4^ to recognize at least the Fbp1 degron. Second, despite the potential to be part of an active E3 ligase, Gid12 precludes productive Fbp1 ubiquitylation (Fig. 6e), by sterically blocking the globular Fbp1 enzymatic domain from accessing the central space within the Chelator-GID^SR4^ (and Cage-GID^SR4^, which encompasses Chelator-GID^SR4^) supramolecular assemblies (Figs. 4, 5). Previous studies showed that catalytic activity of Chelator-GID^SR4^ toward Fbp1 depends on encapsulation of this tetrameric substrate within the central oval^22^; the Chelator assembly establishes avid capture of degrons from multiple Fbp1 protomers, and ubiquitin targeting to multiple protomers simultaneously from the two active sites. Thus, the data suggest that Gid12 potently inhibits Fbp1 ubiquitylation by blocking proper substrate positioning, rather than by blocking potential for its degron recruitment or activation of ubiquitin transfer from the GID E3 partner E2 enzyme Ubc8. Furthermore, our cryo-EM map of Mdh2-bound Chelator-GID^SR4^ (Fig. 6d) suggests Gid12 would generally obstruct substrate encapsulation by higher-order GID E3 assemblies.

In vivo, Gid12 has the potential to inhibit GID E3 substrate ubiquitylation, as shown by the effect of its overexpression when gluconeogenic enzyme substrates are normally degraded (Fig. 7d). Moreover, Gid12 expression inversely correlates with the conditions in which GID E3 activity has been observed (Fig. 7a), Gid4 most stoichiometrically associates with Gid12 under growth on a non-fermentable carbon source, when gluconeogenic enzymes are needed (Fig. 7c). Notably, the levels of Fbp1 tagged at the endogenous locus observed by immunoblotting are generally lower in strains deleted for *GID12* (Fig. 7d). Thus, we speculate that Gid12 may inhibit GID E3 ligase activity to suppress Fbp1 degradation during gluconeogenesis. It seems that after the switch from gluconeogenesis to glycolytic conditions, the production of new Gid4 in substantial excess over Gid12 levels would allow formation of active GID E3 ligases. Future studies will be required to determine whether additional forms of regulation, for example Gid12 degradation, further contribute to rapid activation of glucose-induced GID E3 ligase activity.

The structural and biochemical data also raise new questions about the regulation and function of the GID E3 ligase. For example, does Gid12 form part of an active E3 under other, as yet unidentified, cellular conditions, such as substrates awaiting discovery? Also, a high throughput screen found that *GID12* deletion impacts actin patch formation during endocytosis^39^, but whether this relates to GID E3 ligase function or alternative activities of Gid12 remains to be determined. Interestingly, subunits of the GID E3 were originally identified as playing a role in vacuolar degradation^40^. Although we and others were unable to observe such a role for the GID E3, it is nonetheless tempting to speculate a role for Gid12 in this process. In analogy, a number of uncharacterized proteins have been reported to associate with the human CTLH ortholog of the GID E3, raising the question of whether any of them plays a Gid12-like role^24^. In a related vein, the structures also raise questions about potential functions of Cage-GID^SR4^, for example are there exceptionally large substrates that require its extremely large central cavity. Intriguingly, some metabolic enzymes function in supercomplexes termed “metabolons”^41^. Although not reported for gluconeogenesis enzyme GID E3 ligase substrates, it is noteworthy that mitochondrial Mdh2 from higher organisms does form a “metabolon” with other metabolic enzymes. Thus, we wonder if the metabolic enzyme substrates may transiently form supercomplexes that are targeted by the Cage-GID E3 assembly. Furthermore, caged structures are often associated with coated vesicles, and perhaps Cage-GID^SR4^ functions in the context of vesicles. Future studies will be required to determine if there are distinct biological functions of Cage and Chelator-GID^SR4^ complexes.

Despite the preponderance of ubiquitylation in bioregulation, to date few cellular E3 ligase inhibitory factors have been characterized. Gid12 differs from the best-characterized factors—EMI1 or MCC that prevent premature activity of the multiprotein APC/C E3^42–44^, or GLMN^45,46^, CAND1^47–49^, or CSN inhibitors of multiprotein cullin-RING ligase (CRL) E3s^50–53^—in that it does not bind directly to surfaces required for substrate binding, or to surfaces required to catalyze ubiquitylation. Notably, the CRL regulators CAND1 and CSN have more elaborate functions, as they inhibit various CRL E3 ligase activities in vitro, but activate CRLs in vivo^54^. It is now known that the biological functions of these factors derive from their mediating disassembly of superfluous CRLs and assembly of those newly needed in response to ever-changing needs of cells. We propose that the nuanced effects of Gid12 on GID E3 ligase activities portend intricate regulation of the diverse multiprotein GID E3 assemblies in controlling cellular ubiquitylation.

## Methods

### Construction of plasmids

Coding sequences (cDNAs) of individual GID subunits, Gid3 (Standard name: Ubc8), and substrates (Fbp1, Mdh2, Icl1) were amplified from *S. cerevisiae* S288C genomic DNA using Phusion High-Fidelity DNA polymerase (Thermo Fisher Scientific) and cloned into various vectors using the Gibson assembly method with a home-made Gibson reaction mix. The *Escherichia coli* strain DH5α was used for cloning. To make recombinant GID complexes and subcomplexes by insect cell expression, cassettes encoding GID subunits were combined into one baculoviral expression vector using the biGBac method^55^. The plasmids used in this study are summarized in Supplementary Table 1.

### Yeast strains and growth conditions

Yeast strains used in this study were constructed using standard methods for transformation, mating, sporulation and tetrad dissection^56–58^ and are listed in the Supplementary Table 1. Chromosomally tagged strains and knockout strains were constructed using a PCR-based integration strategy (PMID: 15334558, PMID: 10407276). Standard cloning and site-directed mutagenesis techniques were used. Unless otherwise stated, yeast was grown in YPD (1% yeast extract, 2% peptone, 2% glucose) medium at 30 °C to an OD_600_ of 1.5. Cells were then centrifuged at 500 × *g* for 5 min, washed once with YPE (1% yeast extract, 2% peptone, 2% ethanol) medium, and resuspended in YPE medium to an OD_600_ of 1.0 to start overnight non-fermentable carbon source (ethanol) starvation at 30 °C. The next day, glucose retracted yeast cells were harvested by centrifugation at 500 × *g* for 5 min and resuspended to an OD_600_ of 1.0 in fresh YPD to start carbon recovery.

### Affinity purifications for Gid4, Gid8 and Gid12 for interactome studies

Proteins interacting with endogenously C-terminally FLAG-tagged Gid12 were identified from affinity purifications of BY4741 *GID12-3×FLAG::kanMX4*, compared to wild type BY4741 as background control, grown under glycolytic conditions (Fig. 1a; Supplementary Fig. 1a). Proteins interacting with endogenously C-terminally HA-tagged Gid8 were identified from affinity purifications of BY4741 *GID8-3×HA::kanMX4, Δgid4::natNT2*, compared to wild type BY4741 *GID8-3×HA* yeast strains, grown under glycolytic conditions (Supplementary Fig. 1b). Proteins interacting with endogenously N-terminally HA-tagged Gid4 were identified from affinity purifications of BY4741 *GID12-9×cMYC::kanMX4*, *3×HA-GID4*, compared to BY4741 *GID12-9×cMYC::kanMX4* as a background control, grown under either glycolytic conditions, ethanol starvation for 16 h or carbon recovery conditions (Fig. 7c; Supplementary Fig. 8a).

First, 2 liters of yeast cells were cultured under different growth conditions according to the protocol above, and harvested. Second, harvested cells were lysed by cryomilling (SPEX Sample Prep-6875 Freezer/Mill) and thoroughly dissolved in lysis buffer containing 200 mM NaCl, 50 mM Tris, pH 7.5, EDTA-free protease inhibitor cocktail (cOmplete Tablets, Roche), PhosStop tablets (Roche), 10 μg/ml Leupeptin, 20 μg/ml Aprotinin, 2 mM Benzonase, and 1 mM phenylmethylsulfonyl fluoride (PMSF). The mixture was precleared by centrifugation at 51,428 × *g* for 30 min and the supernatant was incubated with either anti-FLAG M2 resin (Sigma, A2220) or anti-HA resin (Pierce, 26181) and anti-HA magnetic resin (Pierce, 88837) for 2 h with head-over-tail rotation at 4 °C.

For sample preparation via Gid12 affinity purification, first Gid12-3×FLAG bound anti-FLAG M2 resin was washed five times with wash buffer (200 mM NaCl, 50 mM Tris, pH 7.5) to remove nonspecific interactors. The protein-bound resin was first verified by western blotting with 1:5000 diluted anti-FLAG M2 monoclonal antibody (Sigma, F1804) and then submitted for mass spectrometry analysis.

Similarly for sample preparation via Gid8 affinity purification, Gid8-3×HA bound anti-HA magnetic resin (Pierce, 88837) was washed five times with the wash buffer and were further used for mass spectrometry sample preparation steps.

In case of sample preparation by Gid4 affinity purification, 3×HA-Gid4 bound anti-HA resin (Pierce) was transferred into a 0.22 μm VWR centrifugal filter column, followed by five times washing steps with wash buffer. Bound proteins were eluted with elution buffer (wash buffer plus 1 mg/ml HA peptide (Pierce)). Eluted bait proteins were first verified by western blotting with 1:5000 diluted HA-Tag antibody (F-7) (Santa Cruz Biotechnology, sc-7392) and 1:5000 diluted anti-cMyc (9E10) (Santa Cruz Biotechnology, sc-40), then submitted for mass spectrometry analysis.

### Sample preparation for mass spectrometry to identify Gid8-interacting partners and Gid12-interacting partners

For reduction and alkylation of the bound proteins, previously washed anti-FLAG M2 resins or the anti-HA magnetic resins were incubated with SDC buffer containing 1% sodium deoxycholate (SDC, Sigma-Aldrich), 40 mM 2-cloroacetamide (CAA, Sigma-Aldrich), 10 mM Tris (2-carboxyethyl) phosphine (TCEP, Thermo Fisher Scientific) and 100 mM Tris, pH 8.0 at 37 °C. After incubation for 20 min at 37 °C, the samples were diluted 1:2 with MS grade water (VWR). Proteins were digested overnight at 37 °C with 0.5 µg trypsin (Promega). The beads were centrifuged and the supernatant was collected. In addition, the beads were washed with 100 µL of buffer A (0.1% formic acid (Roth) in MS grade water (VWR)), followed by centrifugation and collection of the supernatant. The combined supernatants were acidified with trifluoroacetic acid (TFA, Merck) to a final concentration of 1%. Precipitated SDC was removed by centrifugation and the peptide mixture was desalted via SCX StageTips^54^. Samples were vacuum dried and re-suspended in 10 µl of buffer A.

### Sample preparation for mass spectrometry to identify Gid4-interacting partners

For reduction and alkylation of the proteins, the solution containing eluted proteins were incubated with SDC buffer (see above) for 20 min at 37 °C. The samples were diluted 1:2 with MS grade water (VWR) and were digested overnight at 37 °C with 1 µg trypsin (Promega). The digestion mixture was acidified with TFA to a final concentration of 1%. After removing precipitated SDC, the peptides were desalted using SCX StageTips. Samples were vacuum dried and re-suspended in 10 µl of buffer A.

### Data-dependent acquisition liquid chromatography (LC)-mass spectrometry analysis of IP samples

The peptides were loaded in buffer A (0.1% formic acid) onto a 30 cm column (inner diameter: 75 microns; packed in-house with ReproSil-Pur C18-AQ 1.9-micron beads, Dr. Maisch GmbH) via the autosampler of the Easy-nLC 1200 (Thermo Fisher Scientific) at 60 °C and eluting peptides were directly sprayed either onto the mass spectrometer Orbitrap Exploris 480 (Thermo Fisher Scientific) or the mass spectrometer timsTOF Pro (Bruker Daltonics). For data acquisition on the Orbitrap Exploris 480 coupled to the Easy-nLC, peptides were separated at a flow rate of 300 nL/min by a gradient of buffer B (80% ACN, 0.1% formic acid) from 5 to 30% over 40 min followed by a ramp to 95% over 10 min and finally the percentage of buffer B was maintained at 95% for another 10 min. For data acquisition on the timsTOF Pro coupled to the Easy-nLC, peptides were separated at a flow rate of 400 nL/min by a gradient of buffer B (80% ACN, 0.1% formic acid) from 8 to 30% over 35 min followed by a ramp to 35% over 10 min, then to 58% over the next 5 min, 95% over the next 5 min and maintained at 95% for another 5 min.

The mass spectrometer Orbtrap Exploris 480 was operated in a data-dependent mode with survey scans from 300 to 1650 m/z (resolution of 60,000 at m/z = 200), and up to 15 of the top precursors were selected and fragmented using higher energy collisional dissociation (HCD with a normalized collision energy of value of 30). MS2 spectra were recorded at a resolution of 15,000 (at m/z = 200). AGC target for MS1 and MS2 scans were set to 3 × 106 and 1 × 105 respectively within a maximum injection time of 25 and 28 ms for MS and MS2 scans respectively.

Data acquisition on the mass spectrometer timsTOF Pro was performed using OtofControl 6.2. The mass spectrometer was operated in data-dependent PASEF mode with one survey TIMS-MS and ten PASEF MS/MS scans per acquisition cycle. Analysis was performed in a mass scan range from 100 to 1700 m/z and an ion mobility range from 1/K0 = 1.6 Vs cm^−2^ to 0.6 Vs cm^−2^ using equal ion accumulation and ramp time in the dual TIMS analyzer of 100 ms each at a spectra rate of 9.43 Hz. Suitable precursor ions for MS/MS analysis were isolated in a window of 2 Th for m/z < 700 and 3 Th for m/z > 700 by rapidly switching the quadrupole position in sync with the elution of precursors from the TIMS device. The collision energy was lowered as a function of ion mobility, starting from 59 eV for 1/K0 = 1.6 Vs cm^−2^ to 20 eV for 0.6 Vs cm^−2^. Singly charged precursor ions were excluded with a polygon filter mask and further m/z and ion mobility information was used for ‘dynamic exclusion’ to avoid re-sequencing of precursors that reached a ‘target value’ of 20,000 a.u. The ion mobility dimension was calibrated linearly using three ions from the Agilent ESI LC/MS tuning mix (m/z, 1/K0: 622.0289, 0.9848 Vs cm^−2^; 922.0097 Vs cm^−2^, 1.1895 Vs cm^−2^; 1221.9906 Vs cm^−2^, 1.3820 Vs cm^−2^).

### Data processing and bioinformatics analysis of IP samples

Raw data were processed using the MaxQuant computational platform (version 1.6.17.0 or 2.0.1.0) with standard settings applied for Orbitrap or Bruker Tims data^59,60^. The peak list was searched against the Uniprot database of *S. cerevisiae* (6049 entries, downloaded in July 2020) with an allowed precursor mass deviation of 10 ppm and an allowed fragment mass deviation of 20 ppm. MaxQuant by default enables individual peptide mass tolerances, which was used in the search. Cysteine carbamidomethylation was set as static modification, and methionine oxidation and N-terminal acetylation as variable modifications. The match between run feature was enabled, and proteins were quantified across samples using the label-free quantification (LFQ) algorithm in MaxQuant as LFQ intensities. Further statistical analysis was performed using the Perseus software platform (version 1.6.15.0)^61^. The iBAQ algorithm was used for calculation of approximate abundances for the identified proteins^33^, which normalizes the summed peptide intensities by the number of theoretically observable peptides of the protein.

### Sample preparation for total yeast proteome analysis

50 ml of WT, *gid12*Δ and overexpression Gid12 yeast strains were grown under glucose (for 8 h), ethanol (for 19 h) and recovery conditions (30 mins, 2 h and 4 h). After each time point, the yeast cells were collected by centrifugation (3 mins at 11,200 × *g*). The cell pellets were washed once with water before freezing in liquid nitrogen and stored at −80 °C for further total proteome MS analysis. Yeast growth and sample collection was conducted in triplicates (Supplementary Figs. 8b, 9).

Frozen cell pellets were lysed in SDC Buffer (1% Sodium deoxycholate (wt/vol) in 100 mM Tris pH 8.5) and boiled for 5 min at 95 °C. Lysates were then cooled on ice for 5 min and sonicated using the Bioruptor sonication device for 30 min. Reduction and alkylation was performed by adding Tris(2-carboxyethyl)phosphine (TCEP) and 2-Chloracetamide (CAA) at the final concentrations of 10 mM and 40 mM, respectively, and incubating them for 5 min at 45 °C. Samples were digested overnight by the addition of 1:50 LysC (1:50 wt/wt: Wako) and Trypsin (1:50 wt/wt: Sigma-Aldrich) overnight at 37 °C with agitation (1500 rpm) on an Eppendorf Thermomixer C. The next day, peptides were desalted using SDB-RPS (Empore) StageTips. Briefly, samples were tenfold diluted using 1% TFA in isopropanol and then loaded onto the StageTips, which were subsequently washed with 200 µL of 1% TFA in isopropanol and then with 0.2% TFA/2% acetonitrile (ACN) twice. Peptides were eluted using 75 µL of 80% ACN/1.25% NH_4_OH and dried using a SpeedVac centrifuge (Concentrator Plus; Eppendorf) for 1 h at 30 °C. Peptides were resuspended in 0.2% TFA/2% ACN and peptide concentration was determined using the Nanodrop 2000 (Thermo Scientific). 150 ng of peptides were subjected to LC-MS/MS analysis.

### Data-independent acquisition LC-MS analysis of total yeast proteomes

Peptides were loaded on a 50 cm reversed phase column (75 μm inner diameter, packed in house with ReproSil-Pur C18-AQ 1.9 μm resin). To maintain a column temperature of 60 °C, we used a homemade column oven. An EASY-nLC 1200 system (Thermo Fisher Scientific) was connected online with a mass spectrometer (Orbitrap Exploris 480, Thermo Fisher Scientific) via nano-electrospray source. Peptides were separated using a binary buffer system consisting of buffer A (0.1% formic acid (FA)) and buffer B (80% ACN, 0.1% FA). We used a constant flow rate of 300 nl/min. We loaded 150 ng of peptides and eluted them with a 60 min gradient. The gradient starts with 5% buffer B and increases consistently to 65% in 50 min, until it reaches 95% in 55 min and remains constant for the last 5 min. MS data was acquired using a data independent acquisition (DIA) mode with a full scan range of 300–1650 m/z at 120,000 resolution, automatic gain control (AGC) of 3e6 and a maximum injection time of 20 ms. The higher-energy collision dissociation (HCD) was set to 28. Each full scan was followed by 32 DIA scans which were performed at a 30,000 resolution, an AGC of 1e5 and a maximum injection time of 54 ms.

### Data processing and bioinformatics analysis of total yeast proteomes

DIA raw files were analyzed using directDIA in Spectronaut version 15 (Biognosys). The search was done against UniProt *S. cerevisiae* reference proteome of canonical and isoform sequences with 6077 entries for final protein identification and quantification. Enzyme specificity was set to trypsin with up to two missed cleavages. Maximum and minimum peptide length was set to 52 and 7, respectively. The search included carbamidomethylation as a fixed modification and oxidation of methionine and N-terminal acetylation of proteins as variable modifications. A protein and precursor FDR of 1% were used for filtering and subsequent reporting in samples (*q* value mode with no imputation) The bioinformatics analyses and visualization were done using Perseus version 1.6.1.3^61^ and Python version 3.5.5 with the following packages: pandas 1.3.2, numpy 1.21.2, matplotlib 3.4.3 and seaborn 0.11.2. First, protein intensities were log2-transformed. Next, the dataset was filtered by a minimum of three valid values in at least one experimental group and subsequently imputed using a Gaussian normal distribution (width = 0.3 and downshift = 1.8). Student *t* test was performed using a permutation-based false discovery rate (FDR) of 5% and S0 value of 0.1. Hierarchical clustering was performed using the Euclidian distance.

### Protein expression and purification for cryo-EM

To generate Gid12-SRS (Gid12-Gid4-Gid5-Gid1-Gid8), Gid12-GID^SR4^ (Gid12-Gid4-Gid5-Gid1-Gid8-Gid2-Gid9), Chelator/Cage GID^SR4^ (Gid7-Gid4-Gid5-Gid1-Gid8-Gid2-Gid9) and Gid12-Chelator/Cage GID^SR4^ (Gid12-Gid7-Gid4-Gid5-Gid1-Gid8-Gid2-Gid9) for single particle cryo-EM analysis, Hi5 insect cells were infected with recombinant baculoviruses co-expressing all subunits of a given complex, and grown for 60 h to 72 h in EX-CELL 420 Serum-Free Medium at 27 °C. Cell pellets were resuspended in a lysis buffer containing 50 mM Tris-HCl pH 8.0, 200 mM NaCl, 5 mM DTT, 10 mg/ml leupeptin, 20 mg/ml aprotinin, 2 mM benzamidine, EDTA-free protease inhibitor tablet (1 tablet per 50 mL of the buffer) and 1 mM PMSF. The tagged complexes were first purified from cell lysates by Strep-Tactin affinity chromatography. Gid12-SRS and Gid12-GID^SR4^ were purified based on a Twin-Strep tag fused at the N-terminus of Gid1. Chelator/Cage GID^SR4^ and Gid12-Chelator/Cage GID^SR4^ were purified based on a Twin-Strep tag fused at the C-terminus of Gid8. Further purification was carried on by anion exchange chromatography and size exclusion chromatography in a final buffer containing 25 mM MES pH 6.5, 150 mM NaCl and 1 mM DTT.

### Purification of endogenous yeast Cage-GID for cryo-EM

To purify endogenous GID complex, 3 liters of yeast strain with Gid7 and Gid5 C-terminally tagged at their endogenous loci with 3×HA and 3×FLAG tag, respectively, were grown in YPD (1% yeast extract, 2% peptone, 2% glucose) medium for 8 h. Subsequently, the cells were washed and resuspended in YPE medium (1% yeast extract, 2% peptone, 2% ethanol). The cells were harvested after 18 h growth in YPE medium. The pellet was resuspended in the lysis buffer (50 mM HEPES pH 7.5, 150 mM NaCl, 1 mM CaCl_2_, 0.2 M sorbitol, complete protease inhibitor tablets), frozen in liquid nitrogen in the form of small beads and were subjected to cryogenic grinding using a cryo-mill (SPEX Sample Prep-6875 Freezer/Mill). The obtained yeast powder was thawed and centrifuged at 51,428 × *g* for 10 min, and the resultant supernatant was incubated with anti-FLAG M2 affinity resin for an hour. After thorough washing, the protein was eluted using 3×FLAG peptide and visualized by Coomassie-stained SDS-PAGE. The eluted complex was concentrated to 1 mg/ml and used for cryo-EM grids preparation.

### Sample preparation of Cage-GID^SR4^-Fbp1 for cryo-EM

For cryo-EM sample preparation of Cage-GID^SR4^ with Fbp1, the GID complex consisting of Gid1, Gid2, Gid4, Gid5, Gid7, Gid8-2×Strep and Gid9 were co-expressed in Hi5 insect cells. The lysis buffer used for the purification contained 50 mM NaCl instead of 150 mM NaCl. After the pulldown, GID complex was purified by Strep-Tactin affinity purification, pulling on Twin-Strep tagged C-terminally on Gid8. The eluted protein was then purified by anion-exchange chromatography using Resource Q column. Only the first elution peak, corresponding to the Cage-GID complex was further concentrated and purified by SEC on a Superose 6 column in final buffer containing 25 mM HEPES pH 7.5, 50 mM NaCl and 5 mM DTT. The complex was concentrated to 1 mg/ml, and fivefold molar excess of purified Fbp1 with its N-terminus exchanged to Mdh2 degron (PHSVTP-Fbp1) was added to the complex directly before cryo-EM grids preparation.

### Sample preparation of Chelator-GID^SR4^-Mdh2 for cryo-EM

For cryo-EM sample preparation of Chelator-GID^SR4^-Mdh2, GID complex with Gid1, Gid2, Gid5, Gid8-2×Strep, Gid9 were co-expressed in Hi5 insect cells and purified using Strep-Tactin affinity resins followed by IEX and SEC in buffer containing 25 mM HEPES pH 7.5, 150 mM NaCl and 5 mM DTT. Before SEC, bacterially expressed Gid4 (Δ1-116) and Gid7 were added. The protein complex was then concentrated to 0.6 mg/ml and threefold molar excess of the purified Mdh2-6×His was added directly before cryo-EM grids preparation.

### Cryo-EM grids preparation and data acquisition

Cryo-EM grids were prepared using Vitrobot Mark IV (Thermo Fisher Scientific). 4 µl of freshly purified protein at 1 mg/ml was applied to glow discharged Quantifoil holey carbon grids (R1.2/1.3 200 mesh) at 4 °C with 100% humidity and then immediately blotted with Whatman no.1 filter paper (blot time 10 s, blot force 10) and vitrified by plunging into liquid ethane.

Data collection was carried out at the Max Planck Institute of Biochemistry cryo-EM core facility. For all complexes data were collected on either a Talos Arctica transmission electron microscope or a Glacios transmission electron microscope operated at 200 kV and equipped with a Falcon III or a K2 (Gatan) direct electron detector. Automated data collection was carried out using either EPU software at a nominal magnification of ×73,000 corresponding to 1.997 Å/pixel or using SerialEM (in case of Glacios) with nominal magnification of ×22,000 corresponding to 1.885 Å/pixel, with total exposure of around 60–65 e^−^/Å^2^ and the target defocus range between 1.5 and 3.5 µm.

The data collected using the Talos Arctica for the Gid12-SRS subcomplex yielded a reconstruction at subnanometer resolution, suggesting capacity for high resolution. Thus, the best grids were used for data acquisition using an FEI Titan Krios microscope, installed with a K3 direct electron detector, and operated at 300 kV. Data were collected under a nominal magnification of ×81,000 corresponding to 1.09 Å**/**pixel at the specimen level using a SerialEM multi-record mode. Similarly, the Titan Krios dataset was collected for the Cage-GID^SR4^-Fbp1 under a magnification corresponding to 0.85 Å**/**pixel at the specimen level.

### Data processing

Movie stacks were motion-corrected and dose-weighted using the Motioncor2^62^ program. Contrast transfer function parameters were estimated from dose-weighted, aligned micrographs using Gctf^63^. Particles were picked by the auto-picking program implemented in Relion 3.1 with a reference template generated by manual picked particles. Further processing was carried out using Relion 3.1^64^. Iterative rounds of 2D classifications were performed to clean up the data. 3D classifications were done using the initial model generated. The resulting 3D classes were individually inspected, and those with complete features were selected for further processing. Particles selected from 3D classification were finally re-extracted, re-centered and subjected to auto-refinement (with and without a mask).

For Gid12-SRS and Gid12-GID^SR4^ data, automatic B-factor weighting was performed using post-processing in Relion. Local resolution estimates were performed for Gid12-SRS data, as implemented in Relion. Reported resolutions are based on the gold-standard Fourier Shell Correlation at 0.143 criterion. Processing details for each dataset are provided in Supplementary Figs. 1c–h, 2, 4, 6 and 7. Data collection and 3D reconstruction statistics are provided in Supplementary Tables 2 and 3.

The Titan Krios dataset for Cage-GID^SR4^-Fbp1 was pre-processed with Focus^65^ during data collection, which automatically discarded low quality images. Particles were picked using Laplacian-of-Gaussian algorithm implemented in Relion. The extracted particles were cleaned up by 2D classification, followed by 3D classification using the model of apo Cage-GID^SR4^ as a reference. Finally, the selected set of particles was subject to a masked 3D refinement with imposed D3 symmetry.

### Model building and refinement

The substrate receptor scaffolding module (SRS) from the previous structure of GID^SR4^ (PDB ID: 6SWY)^21^ was readily fit into the 3.3 Å resolution reconstruction of the Gid12-SRS complex. Gid12 was manually built using Coot (v. 0.8.9.1)^66^. Atomic model refinement was performed using ‘phenix.real_space_refine’ available in the PHENIX software suite^67^ and the model was validated using Molprobity^68^. The entire model was checked manually and regions of weak or unclear density were modelled as polyalanine. Model refinement and validation details are given in Supplementary Table 4.

### Composite models of Gid12-GID^SR4^, Gid12-Chelator-GID^SR4^, Gid12-Cage-GID^SR4^ and Cage-GID^SR4^

To generate models of Gid12 bound GID complexes and other assemblies, the structure of Gid12-SRS was docked in maps, along with Gid2-Gid9 (PDB ID: 7NS4)^22^, and for Chelator and Cage assemblies the so-called supramolecular assembly module consisting of two molecules of Gid7 and an additional copy of Gid1 and Gid8 (PDB ID: 7NSB)^22^, using Chimera (v. 1.11.2)^69^ (Supplementary Figs. 3b–c; 5a–b). However, the structures of the connections between the supramolecular assembly module and adjacent proteins in Chelator-GID^SR4^ (EMD-12563) were not modeled previously due to low map resolution, so while the domains at these junctions are known, their relative orientations and the details of interactions remain unknown. Figures of maps and models were prepared with Chimera, ChimeraX (v. 1.2)^70^ and PyMol-v 1.8.2.

### Protein expression and purification for in vitro biochemistry assays

GST-Gid7, 2×Strep-Gid4, 2×Strep-Gid4/Gid12 subcomplex, 2×Strep-Gid4_1-358aa_ (with C terminal 359-362 aa deleted), Mdh2-LPETGG-2×Strep, Fbp1- LPETGG-2×Strep, and Icl1- LPETGG-2×Strep were expressed in Hi5 insect cells. Cell pellets were resuspended in a lysis buffer containing 50 mM Tris-HCl pH 8.0, 200 mM NaCl, 5 mM DTT, 10 mg/ml leupeptin, 20 mg/ml aprotinin, 2 mM benzamidine, EDTA-free protease inhibitor tablet (1 tablet per 50 mL of the buffer) and 1 mM PMSF. The tagged proteins except Gid7 were purified from cell lysates by Strep-Tactin affinity chromatography. GST-Gid7 was purified by glutathione affinity chromatography followed by incubation overnight at 4 °C with TEV protease at a concentration of 0.05 mg/ml to remove the GST tag. 2×Strep-Gid4, 2×Strep-Gid4/Gid12 subcomplex, 2×Strep-Gid4_1-358aa_ were further purified by size exclusion chromatography in a buffer containing 25 mM HEPES pH 7.5, 150 mM NaCl and 1 mM DTT. For Gid7, Mdh2-LPETGG-2×Strep, Fbp1-LPETGG-2×Strep, and Icl1-LPETGG-2×Strep, both anion exchange chromatography and size exclusion chromatography in a final buffer containing 25 mM HEPES pH 7.5, 150 mM NaCl and 1 mM DTT were used for further purification.

GST-Ubc8 (Gid3), Ubc8-LPETGG-6×His and untagged wildtype ubiquitin (WT-Ub) were expressed in *E. coli* BL21(DE3) RIL cells. First, BL21(DE3) RIL cells harboring the respective plasmids were grown at 37 °C in TB medium, followed by lowering of temperature to 21 °C. After the cells reached OD_600_ of 1.0, they were grown for 1–2 h at 18 °C. IPTG was then added to a final concentration of 0.2 mM to induce protein expression. GST-Ubc8 was purified in a similar manner to GST-Gid7. Ubc8-LPETGG-His was purified by Nickel affinity chromatography followed by anion exchange chromatography and size exclusion chromatography. WT Ub was purified from bacterial lysates using the resistance to precipitation in glacial acetic acid method^71^ and further purified by gravity ion exchange chromatography using a home-made S-sepharose column and size exclusion chromatography in a final buffer containing 25 mM HEPES pH 7.5, 150 mM NaCl and 1 mM DTT.

### Fluorescent labeling of substrates and Ubc8

Substrates (Fbp1, Mdh2 and Icl1) with LPETGG-2×Strep tag at the C-termini were labeled with a C-terminal fluorescein (FAM), and Ubc8-LPETGG-2×Strep with a C-terminal TAMRA by sortase-mediated transpeptidation reaction with a fluorescently-labeled peptide as described previously^72^.

### Co-expression affinity purification assay to identify Gid subunits binding Gid12

Baculoviruses that express individual Gid subunits (Strep-Gid1, Gid2, Gid4 Gid5, Gid7, Gid8, Gid9, or Moh1), or combinations of subunits (baculovirus AB1 co-expressing GST-Gid1, Strep-Gid5 and Gid12, AB3 co-expressing GST-Gid1, Gid5 and Gid12, BC2 co-expressing Gid7, Strep-Gid8 and Gid9, BC4 co-expressing Gid7, Gid8 and Gid9and CD4 co-expressing Gid2, Gid4 and Moh1) were mixed in various combinations to test effects of loss of various subunits on interactions with Gid12 (Fig. 1b). To test effects of excluding Gid1, the following baculoviruses were used: Strep-Gid5, Gid12, BC4 and CD4. To test effects of excluding Gid2, the following baculoviruses were used: AB1, BC4, Gid4 and Moh1. To test effects of excluding Gid4, the following baculoviruses were used: AB1, BC4, Gid2 and Moh1. To test effects of excluding Gid5, the following baculoviruses were used: Strep-Gid1, Gid12, BC4 and CD4. To test effects of excluding Gid7, the following baculoviruses were used: AB1, Gid8, Gid9 and CD4. To test effects of excluding Gid8, the following baculoviruses were used: AB1, Gid7, Gid9 and CD4. To test effects of excluding Gid9, the following baculoviruses were used: AB1, Gid7, Gid8 and CD4. To test effects of excluding Gid12, the following baculoviruses were used: Strep-Gid1, Gid5, BC4 and CD4. To test effects of excluding Moh1, the following baculoviruses were used: AB1, BC4, Gid2 and Gid4.

Each combination of baculoviruses was used to infect Hi5 insect cells grown in EX-CELL 420 Serum-Free Medium at 27 °C for 60 h to 72 h. Infected cells were lysed in a buffer containing 50 mM Tris-HCl pH 8.0, 200 mM NaCl, 5 mM DTT, 10 mg/ml leupeptin, 20 mg/ml aprotinin, 2 mM benzamidine, EDTA-free protease inhibitor tablet (1 tablet per 50 mL of the buffer) and 1 mM PMSF. The intact GID complex as well as GID subcomplexes without Gid2, Gid4, Gid7, GID9, or Moh1 were purified from cell lysates by pulldown of N-terminal GST-tagged Gid1. GID subcomplexes without Gid1 or Gid8 were purified by pulldown of N-terminal Twin-Strep-tagged Gid5; while GID subcomplexes without Gid5 or Gid12 were purified by pulldown of N-terminal Twin-Strep-tagged Gid1. Eluted samples were separated by SDS-PAGE (NuPAGE gel, 4-12% Bis-Tris, Thermo Fisher) and visualized by Coomassie blue staining.

### Affinity purification assay to test Gid12 binding to substrate receptors Gid4 and Gid10

Multiple combination of GID subcomplexes containing 2×Strep-Gid1, Gid8, Gid5, Gid4 and Gid10 were co-expressed in Hi5 insect cells, by transfecting cells with baculovirus variants containing the respective coding DNA and were purified by Strep-Tactin affinity purification, pulling on Twin-Strep tagged on the N-terminus of Gid1 (Supplementary Fig. 10c). The pellets were lysed by sonication and lysates were spun at 51,428 × g for 45 min at 4 °C. The supernatant was then incubated with equilibrated Strep-resins at 4 °C for 1 h. The resins were washed several times with the wash buffer containing 50 mM Tris/HCl pH 7.5, 200 mM NaCl and 5 mM DTT and eluted with wash buffer containing 2.5 mM Desthiobiotin. Eluted protein fractions were analyzed by SDS-PAGE on a 12% gel.

### Ubc8~Ub discharge to lysine

The intrinsic E3 ligase activity of various GID complexes was assessed by a substrate-independent pulse-chase assay monitoring discharge of the Ubc8~Ub intermediate to the amino acid lysine, where Ub is transferred from Ubc8 to the lysine (Fig. 6b). In the pulse reaction, thioester-linked Ubc8-TAMRA~Ub was generated by mixing 0.2 µM Uba1, 4 µM Ubc8-TAMRA and 100 µM WT-Ub in a buffer containing 25 mM HEPES pH 7.5, 150 mM NaCl, 5 mM ATP and 5 mM MgCl_2_ at room temperature for 20 min. The pulse reaction was quenched by addition of 50 mM EDTA. The chase reaction was initiated by mixing 5 µl of the pulse reaction containing Ubc8-TAMRA~Ub was mixed with 5 µl “chase initiating” mixture containing 2 µM of the given GID E3 and 25 mM lysine pH 8.0 in a buffer containing 25 mM HEPES pH 7.5 and 100 mM NaCl. The reaction mix was quenched at the indicated time points by mixing the chase reaction with SDS loading buffer without any reducing agent. The samples were heated at 98 °C, loaded onto 12% SDS-PAGE gel, and then visualized on a Typhoon scanner (GE healthcare), additionally visualized with Coomassie staining (Fig. 6b).

### Indirect assay for effect of Gid12 on Gid4 dissociation from GID^SR4^

0.5 µM purified GID^SR4^ and Gid12 bound GID^SR4^ complexes with 3×FLAG tagged at the C-terminus of Gid8 were incubated with 0, 0.5, 1, 1.5, 2, 2.5 µM of 2×Strep-Gid4, or 4 µM of 2×Strep-Gid4^mut^ (Gid4 residues 1-358, lacking the C-terminal four residues that are required to bind Gid5 and incorporate into GID^SR4^)^21^, respectively. The mixtures were gently vortexed and incubated for 20 min at room temperature. Strep-Tactin resin was added to each mixture, incubated at 4 °C for 20 min and washed thoroughly twice with 25 mM HEPES pH 7.5, 150 mM NaCl, 1 mM DTT. 2×Strep-Gid4 and associate proteins were eluted with wash buffer plus 2.5 mM Desthiobiotin. Eluted proteins were separated by SDS-PAGE (NuPAGE gel, 4–12% Bis-Tris, Thermo Fisher) and visualized by Coomassie blue staining (Fig. 6a).

### In vitro ubiquitylation assay

We sought to directly compare effects of Gid12 on different GID E3 assemblies. However, we were unable to produce Gid12 on its own. Instead, we were able to produce Gid12-Gid4, and thus this was compared with Gid4 alone as a reference. Prior to performing the in vitro assays, the inactive anticipatory complex GID^Ant^ (Gid1-Gid2-Gid5-Gid8-Gid9) was incubated with either Gid4 or Gid12-Gid4 to generate GID^SR4^ or Gid12-GID^SR4^, respectively, while addition of Gid7 to the GID^SR4^ or Gid12-GID^SR4^ generated their respective supramolecular assembly versions.

Ubiquitylation assays were carried out using reaction mixtures containing 0.1 µM UBA1 (E1), 2 µM Ubc8 (E2), 0.5 µM GID E3 (complexes mentioned above), 0.5 µM fluorescently-labeled substrates (Mdh2-FAM, Fbp1-FAM, or Icl1-FAM), and 100 µM WT Ub at room temperature in a reaction buffer containing 25 mM HEPES pH 7.5, 150 mM NaCl, 5 mM ATP, 5 mM MgCl_2_ and 0.25 mg/mL BSA (Fig. 6e).

Ubiquitylation was also tested as described for model peptide substrates (Fig. 6c) where the Pro/N-degrons of either Fbp1 or Mdh2 was connected with a flexible linker with lysine at position 27 and a C-terminal fluorophore (Fbp1-Pep*: PTLVNGPRRDSTEGFTGRGWSGRGWSKGGK-FAM; Mdh2-Pep*: PHSVTPSIEQDRLGITGRGWSGRGWSKGGK-FAM)^22^. Peptides were synthesized by the Max Planck Institute of Biochemistry, biochemistry core facility. The reaction mix contained 0.1 µM UBA1 (E1), 2 µM Ubc8 (E2), 0.5 µM GID E3 (complexes mentioned above), 0.5 µM Fbp1-Pep* or Mdh2-Pep* and 100 µM WT Ub. Reactions were carried out at room temperature in a buffer containing 25 mM HEPES pH 7.5, 150 mM NaCl, 5 mM ATP, 5 mM MgCl_2_ and 0.25 mg/mL BSA.

The reactions were quenched at indicated time points by addition of SDS sample loading buffer. Samples were loaded onto 12% SDS-PAGE gels and visualized by Typhoon scanner (GE Healthcare).

### Gluconeogenic enzymes degradation assays

Yeast cells harboring BY4741 *FBP1-3×FLAG*, *GID12-6×HA*; BY4741 *FBP1-3×FLAG*, Δ*gid12*; BY4741 *FBP1-3×FLAG*, *pADH::GID12-6×HA*; BY4741 *MDH2-3×FLAG*, *GID12-6×HA*; BY4741 *MDH2-3×FLAG*, Δ*gid12*; BY4741 *MDH2-3×FLAG*, *pADH::GID12-6×HA*; BY4741 *PCK1-3×FLAG*, *GID12-6×HA;* BY4741 *PCK1-3×FLAG*, Δ*gid12*; BY4741 *PCK1-3×FLAG*, *pADH::GID12-6×HA*; BY4741 *ICL1-3×FLAG*, *GID12-6×HA*; BY4741 *ICL1-3×FLAG*, Δ*gid12*; BY4741 *ICL1-3×FLAG*, *pADH::GID12-6×HA* were grown in YPD/YPE medium as mentioned in the section “Yeast strains and growth conditions”. 1.0 OD_600_ equivalent of yeast cells were harvested and flash frozen in liquid nitrogen after overnight non-fermentable carbon source starvation and at indicated time points during carbon recovery.

Cells were lysed using alkaline lysis (2 M NaOH and 7.5% (v/v) 2-mercaptoethanol for 10 min on ice) followed by trichloroacetic acid precipitation (to a final concentration of 15% for 10 min on ice). Proteins were pelleted by centrifugation at 51,428×g for 10 min, and solubilized in 20 µl 2.5× SDS sample loading buffer at 95 °C for 10 min. Samples were loaded on 12% SDS-PAGE gels and then analyzed by western blotting with 1:5000 diluted anti-FLAG M2 monoclonal (Sigma, F1804), 1:5000 diluted anti-HA (F-7) (Santa Cruz Biotechnology, sc-7392), and 1:50,000 diluted anti-Pgk1 (22C5D8) (Invitrogen, Catalog #459250) antibodies (Fig. 7d). Image was taken with classical film development.

### Gid4 and Gid12 expression profiling

Yeast cells expressing *3×HA-GID4*, *GID12-6×HA* from the endogenous promoter were grown in YPD and YPE medium as described in the section “Yeast strains and growth conditions”. 1.0 OD_600_ equivalent of yeast cells were harvested under following conditions: glycolytic conditions (initial growth in glucose), overnight non-fermentable carbon source starvation (replacing carbon source with ethanol) and at indicated time points during carbon recovery (glucose replenishment), respectively. Cells were lysed followed by sample preparation and immunoblotting with 1:5000 diluted anti-HA (F-7) (Santa Cruz Biotechnology, sc-7392), and 1:50,000 diluted anti-Pgk1 (22C5D8) (Invitrogen, Catalog #459250) antibodies (Fig. 7a). Image was taken with classical film development.

### Gid4 stabilization assay

Yeast cells expressing *3×HA-GID4, GID12-6×HA* and *3×HA-GID4, pGPD-GID12-6×HA* were grown in YPD/YPE medium respectively as mentioned in the section “Yeast strains and growth conditions”. The conditions for cells harvesting, sample preparation and immunoblotting are the same as “Gid4 and Gid12 expression profiling” assay. Final images were taken and analyzed with Amersham^TM^ Imager 600 (Fig. 7b).

### Statistics and reproducibility

Representative results of at least three independent experiments were presented in all of the figure panels. *P* values for all graphs were generated using two-tailed Student’s *t* tests, as indicated in the figure legends; statistical analysis for MS data was performed using the Perseus (v. 1.6.15.0) software platform. For all error bars, data are mean ± s.d.

### Reporting summary

Further information on research design is available in the Nature Research Reporting Summary linked to this article.

## Supplementary information

Supplementary Information

Peer Review File

Reporting Summary

## Data Availability

The structural data generated in this study have been deposited in the EMDB and RCSB databases under accession codes: Gid12-SRS, EMD-32830, PDB ID: 7WUG; Gid12-GID^SR4^, EMD-32831; Gid12-Chelator-GID^SR4^, EMD-32833; Gid12-Cage-GID^SR4^, EMD-32835; Cage-GID^SR4^, EMD-32834; Chelator-GID^SR4^-Mdh2, EMD-14323; Endogenous Cage-GID^Ant^, EMD-14338; Cage-GID^SR4^-Fbp1, EMD-14324; Proteomics data of both the interactomes and the total proteome data will be available upon publication from the ProteomeXchange via the Pride database with the data set identifier PXD028579 [http://proteomecentral.proteomexchange.org/cgi/GetDataset?ID=PXD028579] (interactome) (username: reviewer_pxd028579@ebi.ac.uk and password: 4QjjFS8w) and PXD031713 [http://proteomecentral.proteomexchange.org/cgi/GetDataset?ID=PXD031713] (total proteome) (username: reviewer_pxd031713@ebi.ac.uk and password: msghv4mn) (Supplementary Table 6). Source data are provided with this paper. Biological materials are available on request.

## Source data

Source Data
