# Supplementary material for: Cryo-EM structures of Gid12-bound GID E3 reveal steric blockade as a mechanism inhibiting substrate ubiquitylation
Source: Nat Commun. 2022 Jun 1;13:3041. doi: 10.1038/s41467-022-30803-9 (PMC9160049; doi:10.1038/s41467-022-30803-9)
Supplement: Supplementary file 1 — Supplementary Information [file 41467_2022_30803_MOESM1_ESM.pdf]

Supplementary Information

Cryo-EM structures of Gid12-bound GID E3 reveal steric blockade as a mechanism inhibiting substrate ubiquitylation

Shuai Qiao[1,2], Chia-Wei Lee[1,3,4,*], Dawafuti Sherpa[1,*], Jakub Chrustowicz[1,*], Jingdong Cheng[5], Maximilian Duennebacke[1], Barbara Steigenberger[6], Ozge Karayel[7], Duc Tung Vu[7], Susanne von Gronau[1], Matthias Mann[7], Florian Wilfling[1,8], Brenda A. Schulman[1,9]

1. Department of Molecular Machines and Signaling, Max Planck Institute of Biochemistry, 82152, Martinsried, Germany
2. The Fourth Affiliated Hospital, Zhejiang University School of Medicine, Yiwu, Zhejiang, 322000, China
3. Department of Molecular Structural Biology, Max Planck Institute of Biochemistry, 82152, Martinsried, Germany
4. Department of Molecular Metabolism, Harvard T.H. Chan School of Public Health, Boston, MA 02115, USA
5. Institutes of Biomedical Sciences, Shanghai Key Laboratory of Medical Epigenetics, International Co-laboratory of Medical Epigenetics and Metabolism, University of Fudan, 200032, Shanghai, China
6. Mass Spectrometry Core Facility, Max Planck Institute of Biochemistry, 82152, Martinsried, Germany
7. Department of Proteomics and Signal Transduction, Max Planck Institute of Biochemistry, 82152 Martinsried, Germany
8. Mechanisms of Cellular Quality Control, Max Planck Institute of Biophysics, 60438, Frankfurt am Main, Germany
9. Lead Contact and Correspondence: schulman@biochem.mpg.de

* These authors contributed equally

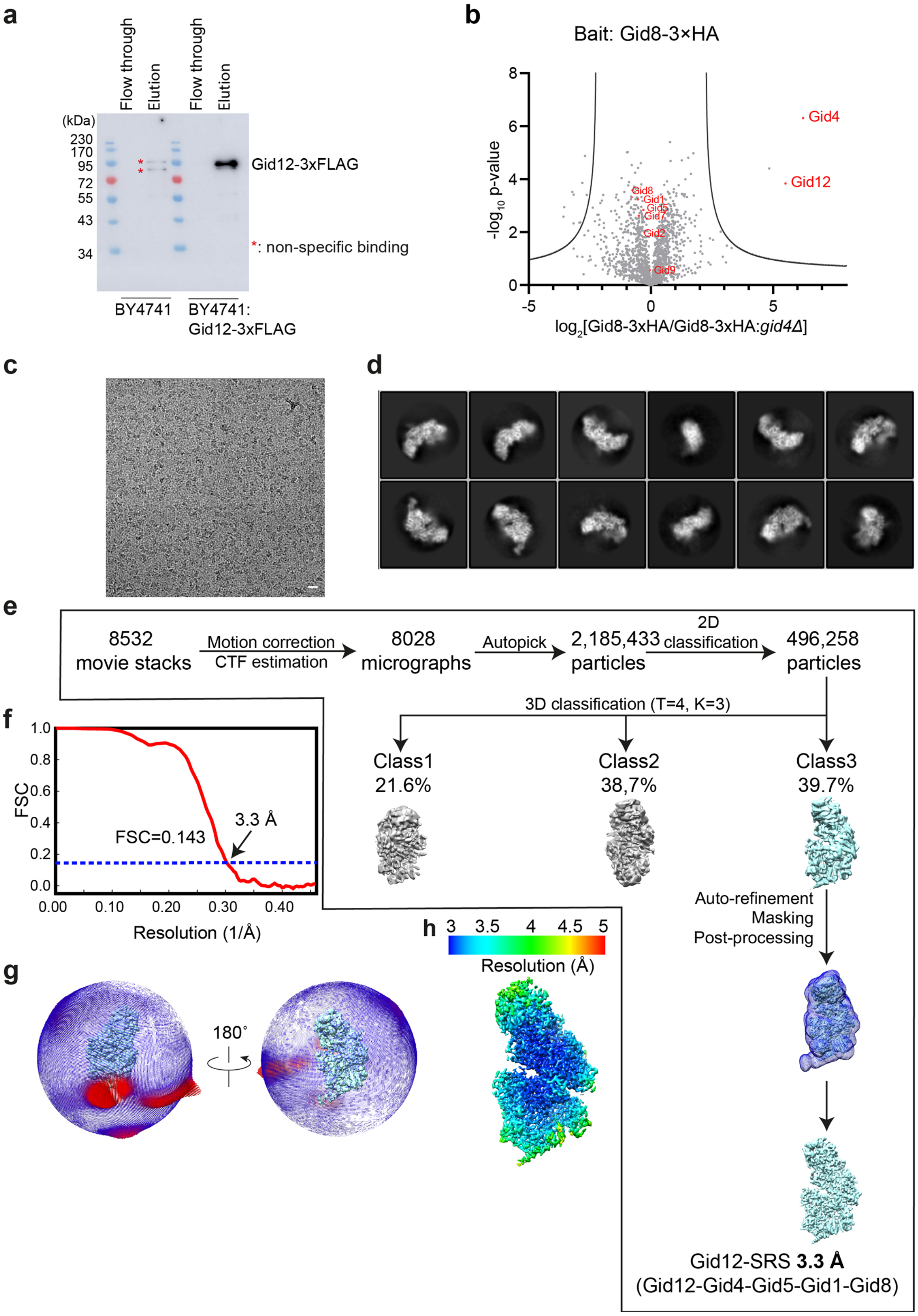
a
Flow through
Elution
Flow through
Elution
(kDa)
230
170
95
72
55
43
34
Gid12-3xFLAG
*: non-specific binding
BY4741
BY4741:
Gid12-3xFLAG
b
Bait: Gid8-3×HA
Gid4
Gid12
Gid8
Gid1
Gid5
Gid7
Gid2
Gid9
-log10 p-value
log2[Gid8-3xHA/Gid8-3xHA:gid4Δ]
c
d
e
8532
movie stacks
Motion correction
CTF estimation
8028
micrographs
Autopick
2,185,433
particles
2D
classification
496,258
particles
3D classification (T=4, K=3)
Class1
21.6%
Class2
38,7%
Class3
39.7%
Auto-refinement
Masking
Post-processing
Gid12-SRS 3.3 Å
(Gid12-Gid4-Gid5-Gid1-Gid8)
f
FSC
FSC=0.143
3.3 Å
Resolution (1/Å)
g
180˚
h
3 3.5 4 4.5 5
Resolution (Å)

Supplementary Figure 1. Analysis of Gid12 binding partners and cryo-EM image processing of the Gid12-SRS subcomplex (Gid1-Gid8-Gid5-Gid4-Gid12)

a. Immunoblot analysis of endogenously purified Gid12 from untagged and Gid12-3xFLAG tagged BY4741 yeast strains using anti-FLAG antibody. Cell lysates from yeasts grown under YPD conditions were subjected to FLAG pulldown assay. Those samples were prepared for Gid12 interactome analysis (n = 3 biologically independent experiments) (Figure 1a).
b. Quantitative MS identified that Gid12 cannot co-immunoprecipitate with Gid8 in a Gid4 deletion strain. Assay was carried out under glycolytic conditions (n=3 technically independent samples). Data are log-transformed ratios of protein LFQ intensities versus −log10-transformed *P* values of two-tailed Student's *t*-tests. The hyperbolic curve separates specifically interacting proteins from background (square; false-discovery-rate-adjusted *P* = 0.01; minimal fold change s0 = 1). The bait protein is Gid8-3xHA.
c. Representative cryo-EM image of Gid12-SRS particles on cryo-EM grids. Scale bar, 15 nm.
d. Reference-free 2D class averages of Gid12-SRS.
e. Flow chart showing classification and refinement steps for cryo-EM data of Gid12-SRS.
f. Fourier shell correlation curve with the blue dotted line indicating the 0.143 cut-off criterion for nominal resolution.
g. Angular distribution of particles used for 3D reconstruction of Gid12-SRS.
h. Color-coded surface representations of Gid12-SRS map based on the local resolution.

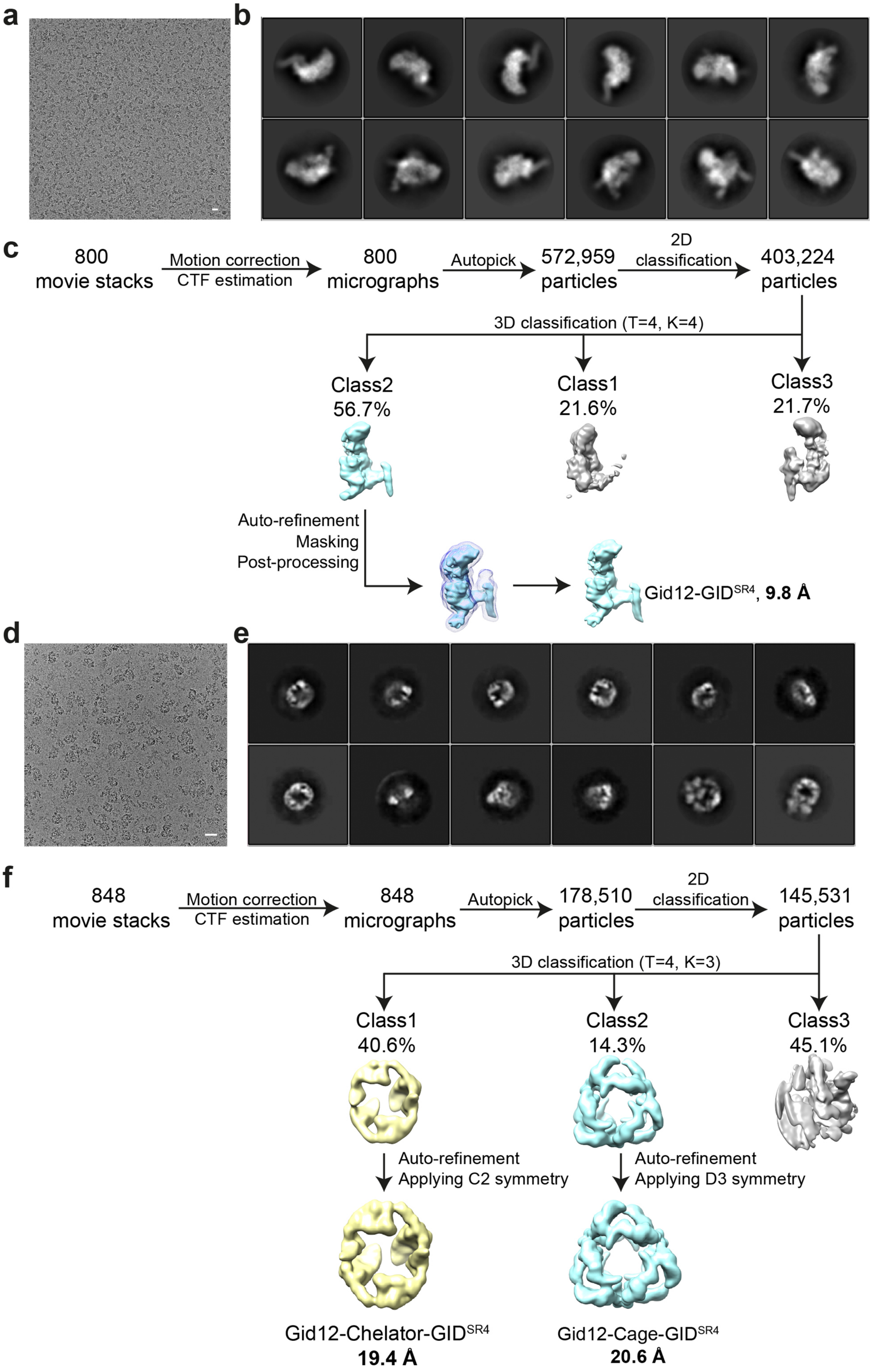

a
b
c
800 movie stacks
Motion correction
CTF estimation
800 micrographs
Autopick
572,959 particles
2D classification
403,224 particles
3D classification (T=4, K=4)
Class2 56.7%
Class1 21.6%
Class3 21.7%
Auto-refinement
Masking
Post-processing
Gid12-GID^SR4, 9.8 Å
d
e
f
848 movie stacks
Motion correction
CTF estimation
848 micrographs
Autopick
178,510 particles
2D classification
145,531 particles
3D classification (T=4, K=3)
Class1 40.6%
Class2 14.3%
Class3 45.1%
Auto-refinement
Applying C2 symmetry
Auto-refinement
Applying D3 symmetry
Gid12-Chelator-GID^SR4
19.4 Å
Gid12-Cage-GID^SR4
20.6 Å

Supplementary Figure 2. Cryo-EM data processing of Gid12-GID^{SR4} (Gid1-Gid2-Gid4-Gid5-Gid8-Gid9-Gid12), Gid12-Chelator-GID^{SR4} and Gid12-Cage-GID^{SR4} (Gid1-Gid2-Gid4-Gid5-Gid7-Gid8-Gid9-Gid12).

a. Micrograph showing Gid12-GID^{SR4} particles in cryo-EM grids. Scale bar, 20 nm.
b. Reference-free 2D class averages of Gid12 -GID^{SR4}.
c. Flow chart of classification and refinement steps for cryo-EM data of Gid12 -GID^{SR4}.
d. Micrograph showing Gid12-Chelator-GID^{SR4} and Gid12-Cage-GID^{SR4} particles in cryo-grids. Scale bar, 40 nm.
e. Reference-free 2D class averages of Gid12-Chelator-GID^{SR4} and Gid12-Cage-GID^{SR4}.
f. Flow chart of 3D classification and refinement steps for cryo-EM data of Gid12-Chelator-GID^{SR4} and Gid12-Cage-GID^{SR4}.

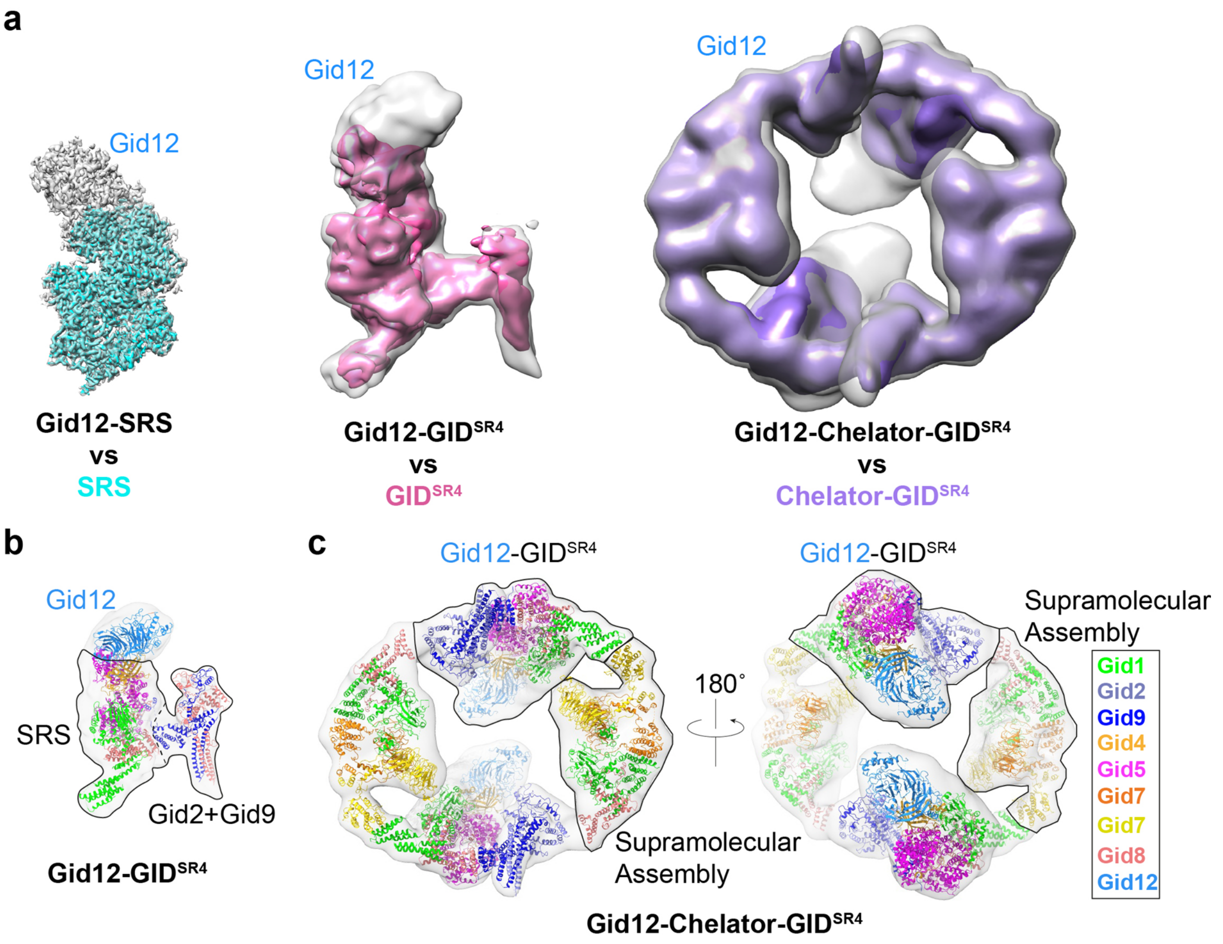

Supplementary Figure 3. Gid12 bound density maps and atomic coordinates of Gid12-GID^{SR4} and Gid12-Chelator-GID^{SR4}.

a. Determination of the density of Gid12 by superimposing Gid12-bound maps with previously reconstructed structures. The density map of substrate receptor scaffolding module (SRS: Gid1-Gid8-Gid5-Gid4; EMD-10330) was fitted into Gid12-SRS map (left). Likewise, GID^{SR4} map (Gid1-Gid8-Gid5-Gid4-Gid2-Gid9; EMD-10327) was fitted into Gid12-GID^{SR4} map (middle), while Chelator-GID^{SR4} map (Gid1-Gid8-Gid5- Gid4-Gid7-Gid2-Gid9; EMD-12541) was fitted into Gid12-Chelator-GID^{SR4} map (right).
b. Incorporation of composite atomic coordinates of Gid12-GID^{SR4} into its 3D reconstruction map.
c. Incorporation of composite atomic coordinates of Gid12-Chelator-GID^{SR4} into its 3D reconstruction map.

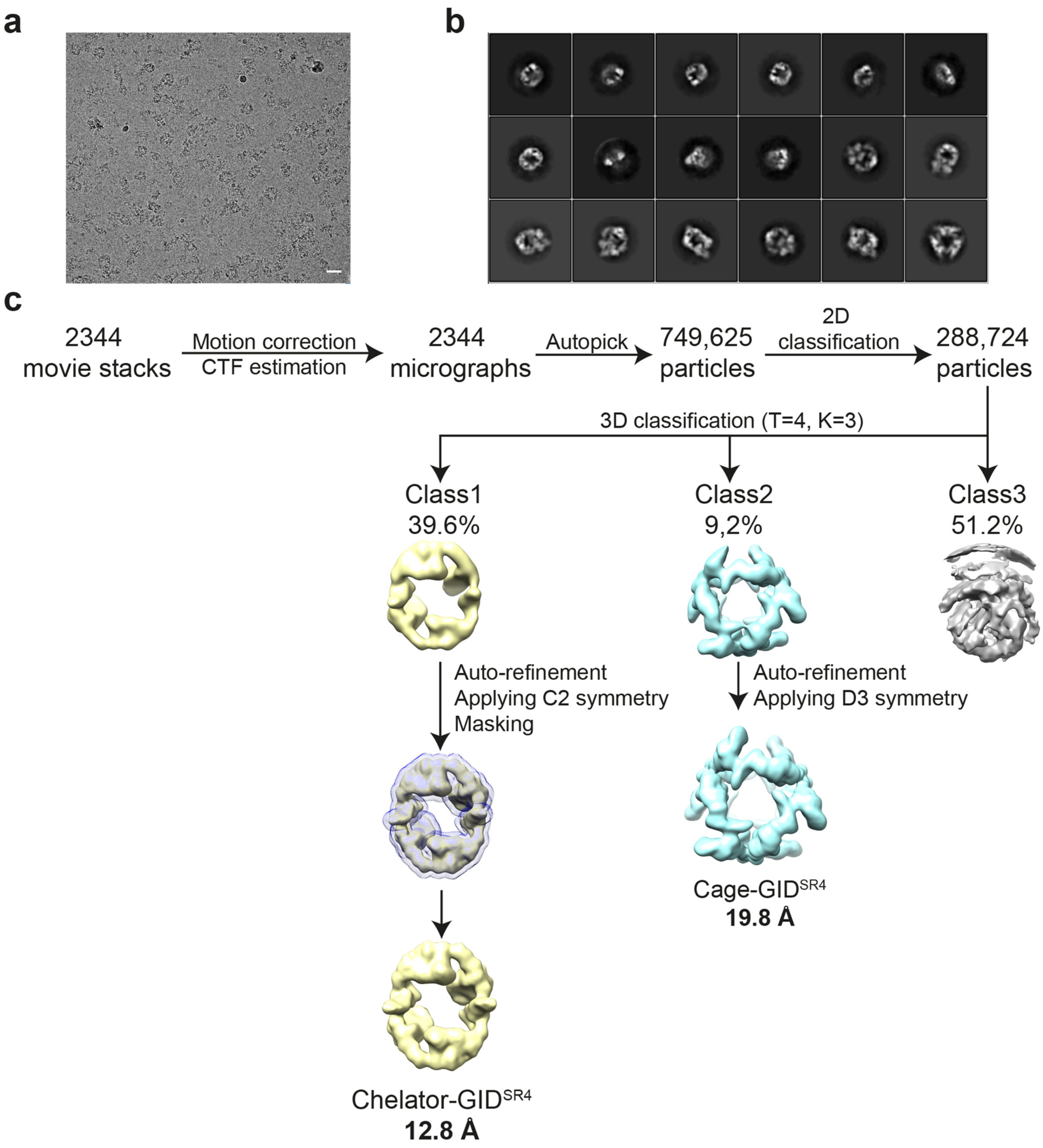

Supplementary Figure 4. Cryo-EM data analysis of Chelator-GID^{SR4} and Cage-GID^{SR4} (Gid1-Gid2-Gid4-Gid5-Gid7-Gid8-Gid9).

a. Representative micrograph showing Chelator-GID^{SR4} and Cage-GID^{SR4} particles in cryo-EM grids. Scale bar, 40 nm.
b. Reference-free 2D class averages of Chelator-GID^{SR4} and Cage-GID^{SR4}.
c. Flow chart of classification and refinement steps for cryo-EM data of Chelator-GID^{SR4} and Cage-GID^{SR4}.

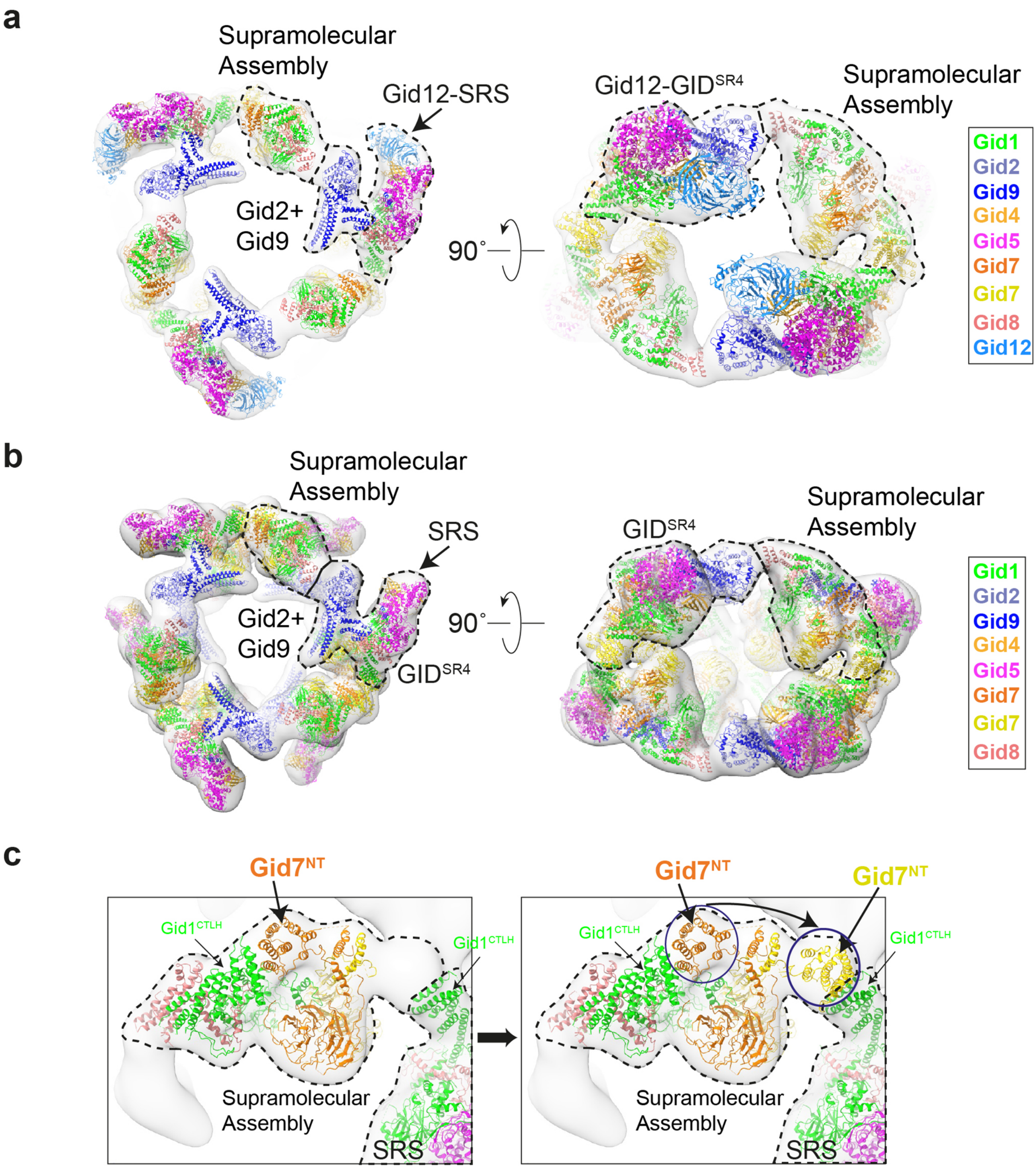

Supplementary Figure 5. Overall architecture of the different Cage-GID complexes.

a. Incorporation of composite atomic coordinates of Gid12-Cage-GID^{SR4} into its 3D reconstruction map.
b. Incorporation of composite atomic coordinates of Cage-GID^{SR4} into its 3D reconstruction map.
c. Closeups highlighting the coordinates of Gid7 N-terminal domain (Gid7^{NT}) interacting with CTLH domain of Gid1 (Gid1^{CTLH}) in the supramolecular assembly module (PDB: 7NSB, shown in left). These coordinates of Gid7^{NT} was used further to substitute the missing Gid7^{NT} coordinates in the supramolecular assembly module (right) which connects to CTLH domain of Gid1 in the SRS module.

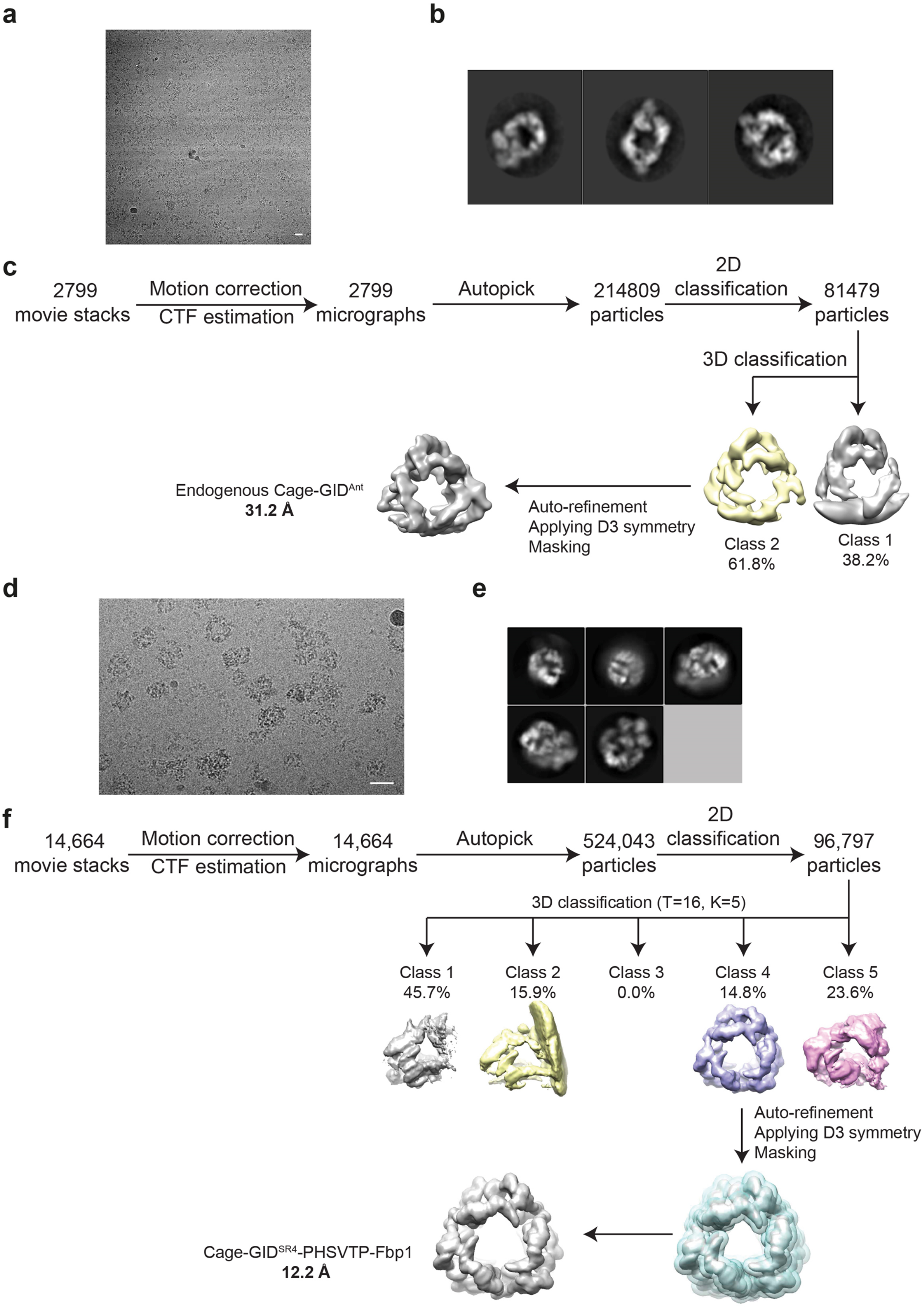
a
b
c
2799
movie stacks
Motion correction
CTF estimation
2799
micrographs
Autopick
214809
particles
2D
classification
81479
particles
3D classification
Endogenous Cage-GID^Ant
31.2 Å
Auto-refinement
Applying D3 symmetry
Masking
Class 2
61.8%
Class 1
38.2%
d
e
f
14,664
movie stacks
Motion correction
CTF estimation
14,664
micrographs
Autopick
524,043
particles
2D
classification
96,797
particles
3D classification (T=16, K=5)
Class 1
45.7%
Class 2
15.9%
Class 3
0.0%
Class 4
14.8%
Class 5
23.6%
Auto-refinement
Applying D3 symmetry
Masking
Cage-GID^SR4-PHSVTP-Fbp1
12.2 Å

Supplementary Figure 6. Cryo-EM data analysis of endogenous Cage-GID^{Ant} and Cage-GID^{SR4}-PHSVTP-Fbp1 datasets.

a. Representative micrograph showing endogenous Cage-GID^{Ant} particles in cryo-EM grids. Scale bar, 40 nm.
b. Reference-free 2D class averages of endogenous Cage-GID^{Ant}.
c. Flow chart of classification and refinement steps for cryo-EM data of endogenous Cage-GID^{Ant}.
d. Representative micrograph showing Cage-GID^{SR4}-PHSVTP-Fbp1 particles in cryo-EM grids. Scale bar, 40 nm.
e. Reference-free 2D class averages of Cage-GID^{SR4}-PHSVTP-Fbp1.
f. Flow chart of classification and refinement steps for cryo-EM data of Cage-GID^{SR4}-PHSVTP-Fbp1.

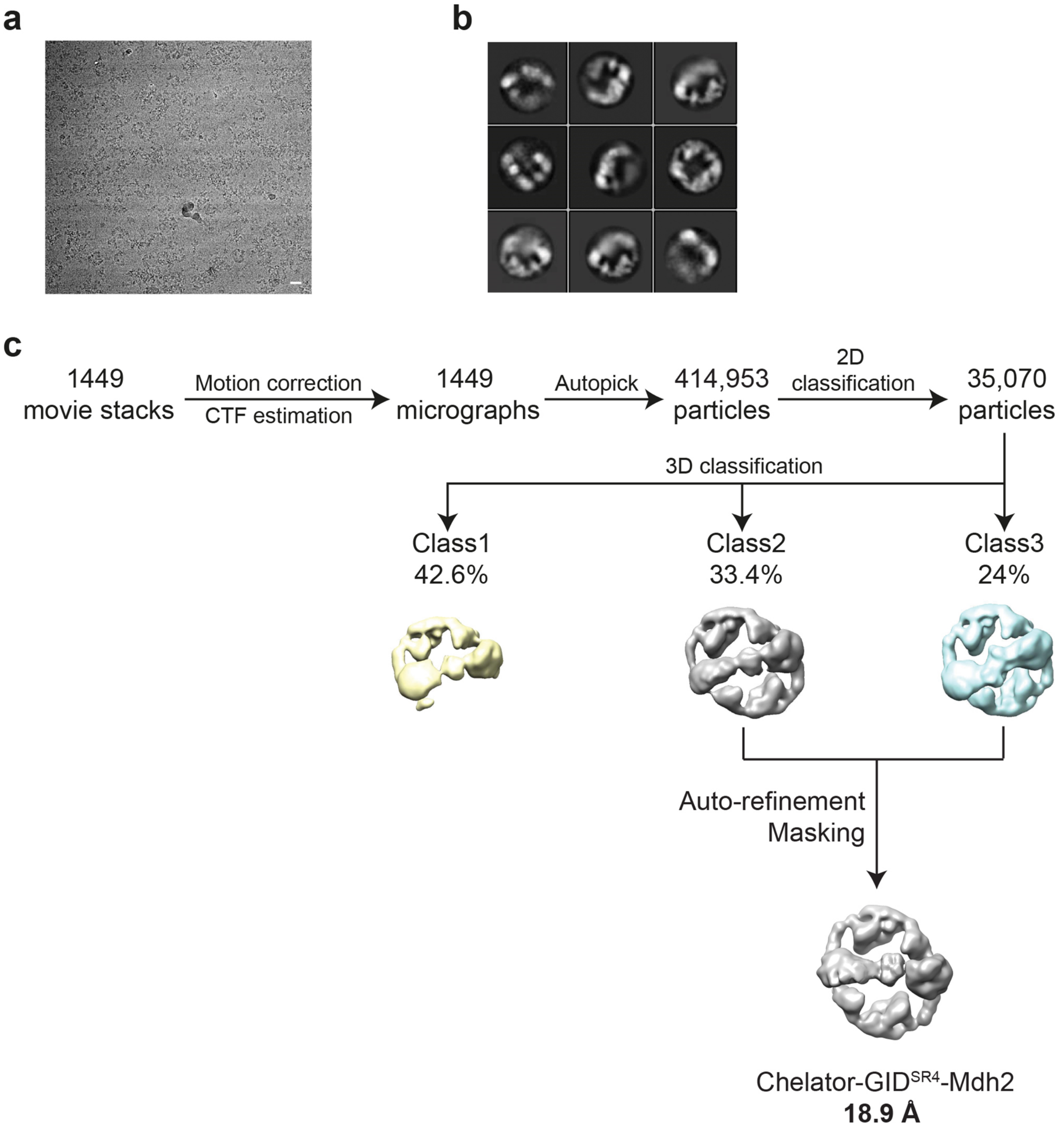

Supplementary Figure 7. Cryo-EM data analysis of Chelator-GID^{SR4}-Mdh2 dataset.

a. Representative micrograph showing Chelator-GID^{SR4}-Mdh2 particles in cryo-EM grids. Scale bar, 30 nm.
b. Reference-free 2D class averages of Chelator-GID^{SR4}-Mdh2.
c. Flow chart of classification and refinement steps for cryo-EM data of Chelator-GID^{SR4}-Mdh2.

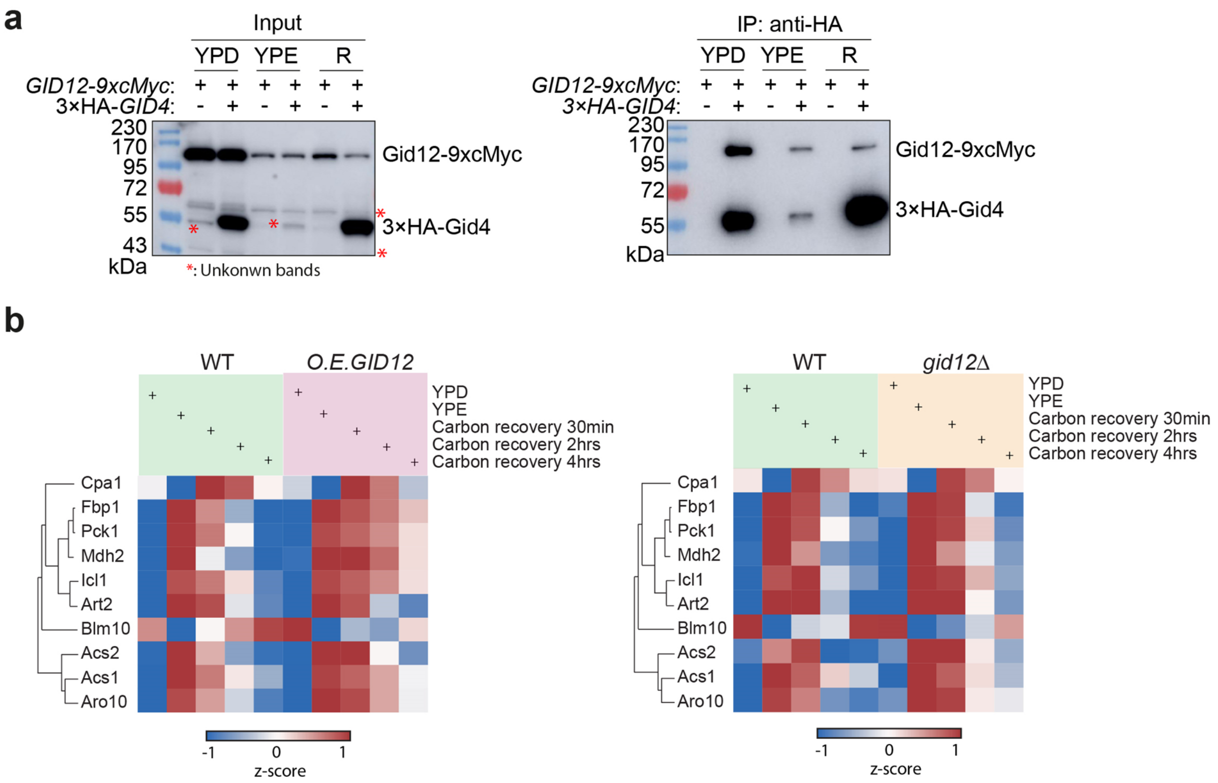

Supplementary Figure 8. Analyses of Gid4 interactome and total yeast proteomes

a. Immunoblot analysis of immunoprecipitated Gid4 from a Gid12-9xcMyc and a 3xHA-Gid4 Gid12-9xcMyc double tagged BY4741 yeast strain using anti-HA antibody. Cells were cultured under YPD, YPE and carbon recovery (R) conditions. The left image represents the input sample of the lysate before co-IP, the right image shows the results of anti-HA co-IP. The 3xHA-Gid4 and Gid12-9xcMyc tagged proteins were detected using anti-HA and anti-cMyc antibodies, respectively. The co-IP samples obtained were further submitted to qMS for Gid4 interactome analysis (n = 3 biologically independent experiments).
b. Heat map showing z-scored protein abundance (log2 protein intensities) from total proteome analysis of WT versus *gid12*Δ, or WT versus *GID12* overexpression (O.E.*GID12*) strains under different metabolic conditions (YPD, YPE, recovery at 30 mins, 2 hours and 4 hours) (n=3 independent biological replicates).

Substrate receptor Gid4

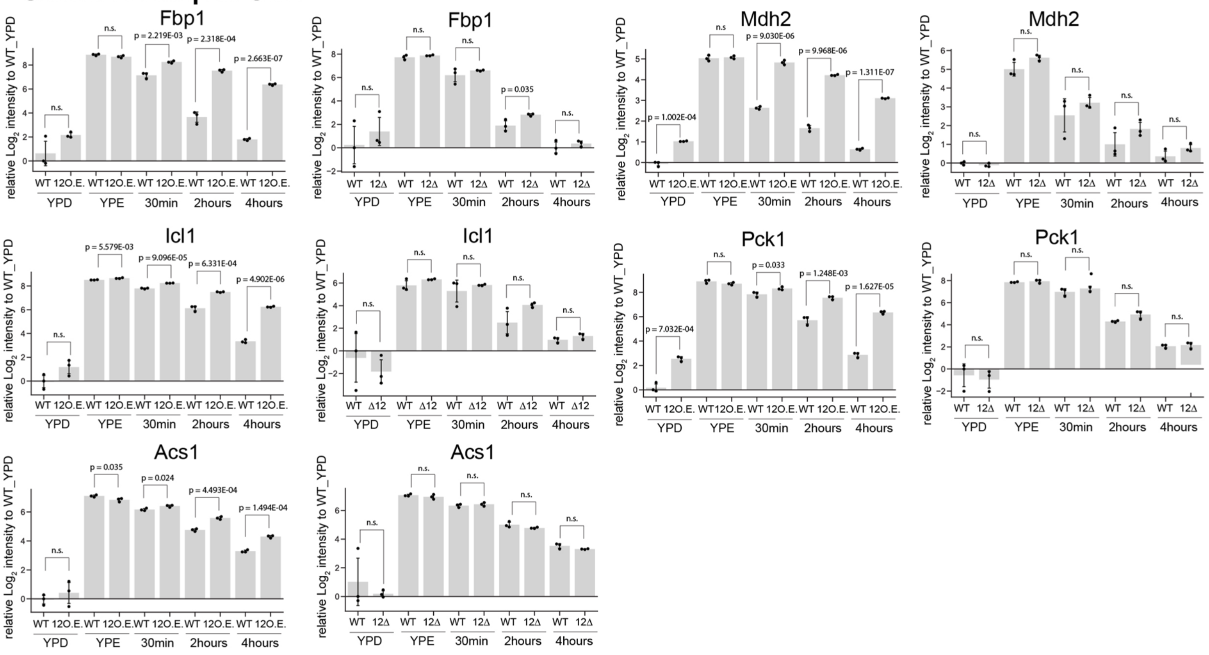

Substrate receptor Gid10

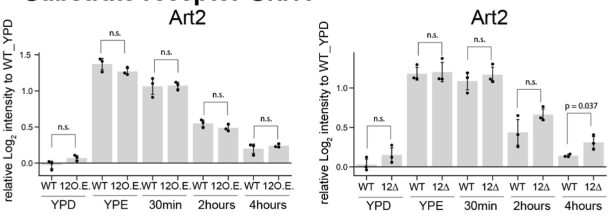

Pro/N-degron substrate receptor unassigned

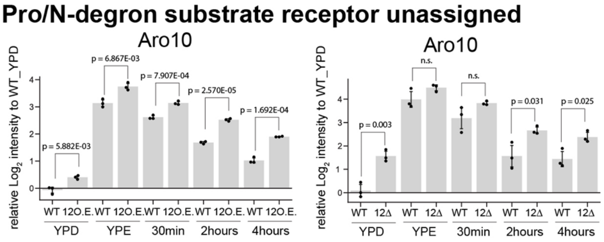

Substrate receptor Gid11

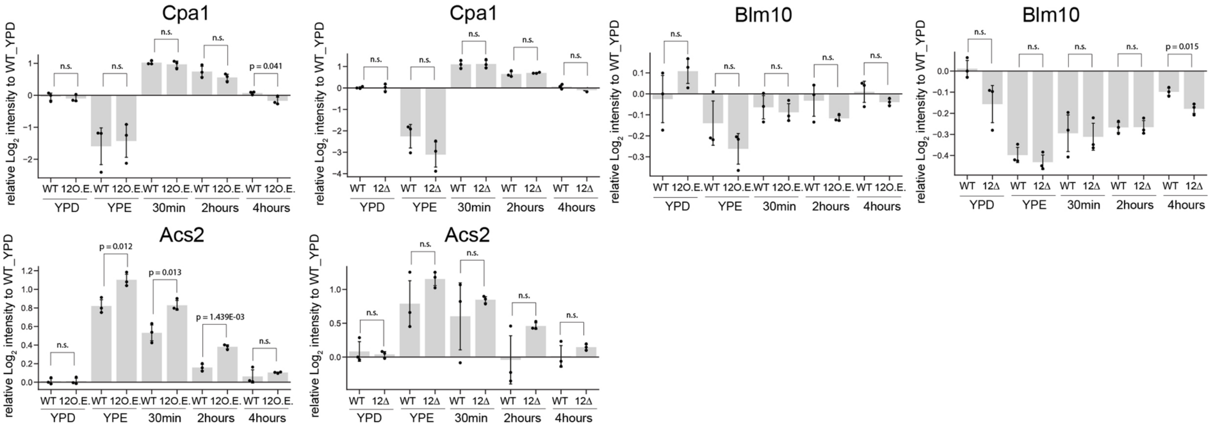

Gid subunits

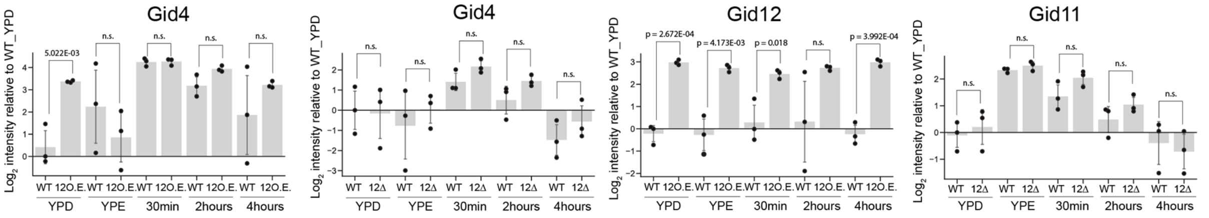

Supplementary Figure 9. Effect of Gid12 abundance on the degradation of known GID substrates.

Bar graphs showing relative levels of different substrates and substrate receptors of GID under different conditions (YPD, YPE and recovery at 30 mins, 2 hours and 4 hours) in WT, *gid12Δ* and *GID12* overexpression (O.E. *GID12*) yeast strains. Error bars represent the standard deviation of three biological replicates. Significance was determined using an unpaired two-tailed Student's t-test (ns., $p \geq 0.05$, degree of freedom = 4).

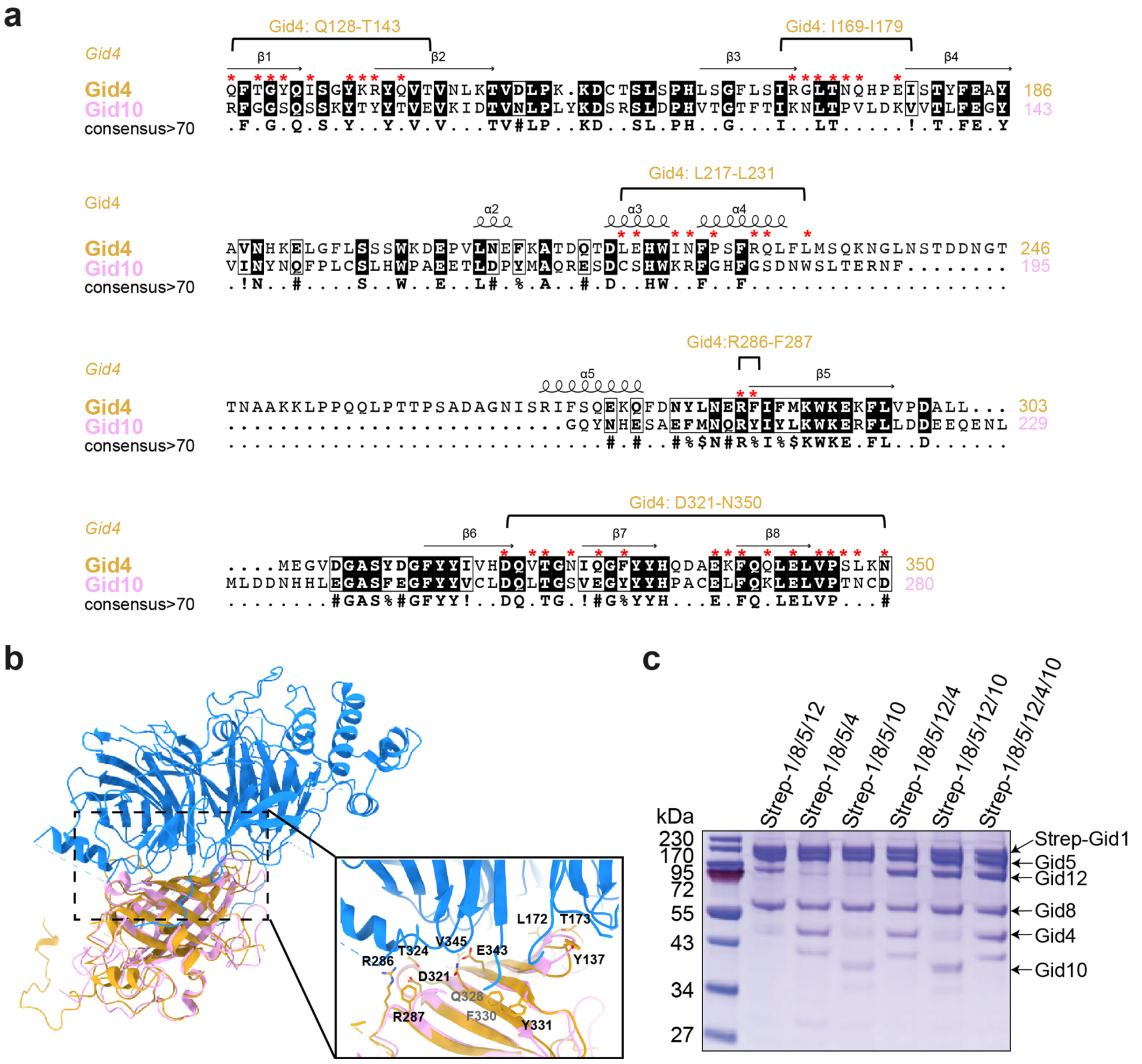

Supplementary Figure 10. Potential for other Gid substrate receptors to bind Gid12

a. Sequence alignment showing that the substrate binding domains of Gid4 and Gid10 are highly conserved. The Gid4 residues buried in the Gid12-Gid4 interface are braced and labeled on its top. The Gid4 residues in contact with Gid12 are labelled with a star on top. Gid4 is colored gold, Gid10 in pink.

b. Structural alignment of Gid10 (PDB:7QQY) and Gid12 bound Gid4 (0.93 Å RMSD) shows a similar Gid12-binding interface between the two substrate receptors, which indicates Gid10 can potentially bind Gid12. In the close-up, Gid4 residues in contact with Gid12 are shown as sticks, identical residues to those in Gid10 are shown in black whereas similar residues are shown in grey.

c. SDS-PAGE of Strep-Gid1 affinity purifications after co-infecting insect cells with respective Gid subunits indicated above the lane. The results indicate substantial binding of Gid12 with Gid4 and Gid10 (n = 3 biologically independent experiments).

Supplementary Tables

Supplementary Table 1. List of strains and plasmids used in this study

Strain or plasmid	Selection marker	Reference
**Yeast strains**		
Saccharomyces cerevisiae S288C: BY4741; MATa his3Δ1 leu2Δ0 met15Δ0 ura3Δ0		Euroscarf (Cat#Y00000)
BY4741 *GID12-3×FLAG::kanMX4*	kanMX4	This study
BY4741 *GID12-9×cMYC::kanMX4*	kanMX4	This study
BY4741 *GID12-9×cMYC::kanMX4*, *3×HA-GID4*	kanMX4, HIS3MX6	This study
BY4741 *GID12-6×HA::HIS3MX6*, *FBP1-3×FLAG::kanMX4*	kanMX4, HIS3MX6	This study
BY4741 *GID12-6×HA::HIS3MX6*, *MDH2-3×FLAG::kanMX4*	kanMX4, HIS3MX6	This study
BY4741 *GID12-6×HA::HIS3MX6*, *PCK1-3×FLAG::kanMX4*	kanMX4, HIS3MX6	This study
BY4741 *GID12-6×HA::HIS3MX6*, *ICL1-3×FLAG::kanMX4*	kanMX4, HIS3MX6	This study
BY4741 *gid12Δ::natNT2*, *FBP1-3×FLAG ::kanMX4*	kanMX4, natNT2	This study
BY4741 *gid12Δ::natNT2*, *MDH2-3×FLAG ::kanMX4*	kanMX4, natNT2	This study
BY4741 *gid12Δ::natNT2*, *PCK1-3×FLAG ::kanMX4*	kanMX4, natNT2	This study
BY4741 *gid12Δ::natNT2*, *ICL1-3×FLAG ::kanMX4*	kanMX4, natNT2	This study
BY4741 *natNT2::P$_{GPD}$-GID12-6×HA::HIS3MX6*, *FBP1-3×FLAG::kanMX4*	kanMX4, HIS3MX6	This study
BY4741 *natNT2::P$_{GPD}$-GID12-6×HA::HIS3MX6*, *MDH2-3×FLAG::kanMX4*	kanMX4, HIS3MX6	This study
BY4741 *natNT2::P$_{GPD}$-GID12-6×HA::HIS3MX6*, *PCK1-3×FLAG::kanMX4*	kanMX4, HIS3MX6	This study
BY4741 *natNT2::P$_{GPD}$-GID12-6×HA::HIS3MX6*, *ICL1-3×FLAG::kanMX4*	kanMX4, HIS3MX6	This study
BY4741; gid12Δ::kanMX4	kanMX4	This study
BY4741; gid10::natNT2-P$_{GPD}$-GID10	natNT2	Langlois et al. 2022
BY4741; gid10::3xFLAG-GID10 gid8::GID8-3xHA-kanMX4	kanMX4	This study
BY4741; gid10::3xFLAG-GID10 gid8::GID8-3xHA-kanMX4, gid4Δ::natNT2	kanMX4, natNT2	This study
BY4741; gid7;;gid7-3xHA-hphNT1, gid5;;gid5-3xFLAG-kanMX4	kanMX4, hphNT1	Sherpa et al. 2021
**Plasmids**		
*p*FLN2-GID1	Ampicillin	This study
*p*FLN2-Strep-GID1	Ampicillin	This study
*p*FLN2-GID2	Ampicillin	This study
pFLN2-GID4	Ampicillin	This study
*p*FLN2-GID5	Ampicillin	This study
*p*FLN2-Strep-GID5	Ampicillin	This study
*p*FLN2-GID7	Ampicillin	This study
*p*FLN2-GID8-C*2×Strep	Ampicillin	This study
*p*FLN2-GID9	Ampicillin	This study
*p*FLN2-GID12	Ampicillin	This study
*p*FLN2-Moh1	Ampicillin	This study
*p*GA-GST-GID1/2×Strep-GID5/GID12	Ampicillin	This study
*p*GA-GST-GID1/GID5/GID12	Ampicillin	This study
*p*GA-GID7/GID8/GID9	Ampicillin	This study
*p*GA-GID7/Strep-GID8/GID9	Ampicillin	This study
*p*GA-GID2/GID4/Moh1	Ampicillin	This study

*p*GA-GID1/GID5/GID12	Ampicillin	This study
*p*GA-Strep-GID1/GID5/GID12	Ampicillin	This study
*p*GA-GID7/GID8- C*2×Strep /GID9	Ampicillin	This study
*p*GA-GID8/GID9	Ampicillin	This study
*p*GA-GID2/GID4/MOH1	Ampicillin	This study
*p*GA-GID8- C*2×Strep /GID9	Ampicillin	This study
*p*GA- Strep-GID1/GID5/GID12/GID8 /GID9/ GID2/GID4/MOH1	Ampicillin	This study
*p*GA- GID1/GID5/GID12/ GID7/GID8- C*2×Strep /GID9/ GID2/GID4/MOH1	Ampicillin	This study
*p*FLN2-Gst-Gid7	Ampicillin	This study
*p*GEX-Gid7	Ampicillin	This study
*p*GEX-Gid3	Ampicillin	This study
*p*ET15-wtUb	Ampicillin	Lab storage
*p*RSF-duet1-Gid3-LPETGG-6×HIS	Kanamycin	This study
*p*RSF-duet1-Fbp1-LPETGG-6×HIS	Kanamycin	This study
*p*RSF-duet1-Mdh2-LPETGG-6×HIS	Kanamycin	This study
*p*RSF-duet1-Icl1-LPETGG-6×HIS	Kanamycin	This study
*p*FLN2-Strep-GID4	Ampicillin	This study
*p*FLN2-Strep-GID4(1-358aa)	Ampicillin	This study
*p*FLN2-Strep-GID4/Gid12	Ampicillin	This study
*p*GA- GID1/GID5/GID8- C*3×FLAG /GID9/ GID2/GID4/MOH1	Ampicillin	This study
*p*GA- GID1/GID5/GID12/GID8- C*3×FLAG /GID9/ GID2/GID4/MOH1	Ampicillin	This study
Bacterial strains		
E.coli BL21 (DE3) RIL	Chloramphenicol	Stratagene (Cat#230245)

Supplementary Table 2. Cryo-EM data collection, 3D reconstruction and map refinement

	Gid12-SRS	Gid12-GID^{SR4}	Chelator-GID^{SR4}-Mdh2	Gid12-Chelator-GID^{SR4}	Endogenous Cage-GID^{Ant}	Cage-GID^{SR4}	Cage-GID^{SR4}-Fbp1	Gid12-Cage-GID^{SR4}
EMDB Code	EMD-32830	EMD-32831	EMD-14323	EMD-32833	EMD-14338	EMD-32834	EMD-14324	EMD-32835
Microscope/ Detector	Krios/ K3	Arctica/ Falcon III	Glacios K2	Arctica/ Falcon III	Glacios/K2	Arctica/ Falcon III	Krios/K3	Arctica/ Falcon III
Particles	197,014	228,628	20,164	58,212	50,416	26,562	14,371	20,810
Pixel size	1.09	1.612	1.885	1.997	1.885	1.997	0.85	1.997
Defocus range (μM)	1.1-3.2	1.5-3.5	1.5-3	1.5-3.5	1.5-3	1.5-3.5	0.7-2.5	1.5-3.5
Voltage (kv)	300	200	200	200	200	200	300	200
Electron dose (e^-/ Å^2/s)	6.675	21.3	3.8	23.07	3.7	21.4	11.43	22.4
Exposure time (s)	8	3	16	3	16	3	6	3
Map Resolution (Å)[a]	3.3	9.8	18.9	19.4	31.2	19.8	12.2	20.6
FSC threshold	0.143	0.143	0.143	0.143	0.143	0.143	0.143	0.143
Map resolution range (Å)	3.3-7.2	N/A	N/A	N/A	N/A	N/A	N/A	N/A
Map sharpening B-factor (Å^2)	-92	-70	N/A	N/A	N/A	N/A	N/A	N/A

[a]According to the Fourier Shell Correlation (FSC) cut-off criterion of 0.143 defined in [1]

Supplementary Table 3. GID complexes newly identified and with 3D reconstructions in this study

GID complex	Subunits	Stoichiometry	Number of subunits
Gid12-SRS	Gid1, Gid5, Gid8, Gid4, Gid12	1:1:1:1:1	5
Gid12-GID^{SR4}	Gid1, Gid5, Gid8, Gid2, Gid9, Gid4, Gid12	1:1:1:1:1:1:1	7
Chelator-GID^{SR4}-Mdh2	Gid1, Gid5, Gid8, Gid2, Gid9, Gid7, Gid4, Mdh2 dimer	4:2:4:2:2:4:2:1	21
Gid12-Chelator-GID^{SR4}	Gid1, Gid5, Gid8, Gid2, Gid9, Gid7, Gid4, Gid12	4:2:4:2:2:4:2:2	22
Cage-GID^{Ant}	Gid1, Gid5, Gid8, Gid2, Gid9, Gid7	12:6:12:6:6:12	54
Cage-GID^{SR4}	Gid1, Gid5, Gid8, Gid2, Gid9, Gid7, Gid4,	12:6:12:6:6:12:6	60
Cage-GID^{SR4}-Fbp1	Gid1, Gid5, Gid8, Gid2, Gid9, Gid7, Gid4, PHSVTP-Fbp1 tetramer	12:6:12:6:6:12:6:3	63
Gid12-Cage-GID^{SR4}	Gid1, Gid5, Gid8, Gid2, Gid9, Gid7, Gid4, Gid12	12:6:12:6:6:12:6:6	66

Supplementary Table 4. Model refinement and validation statistics

Map	Gid12-SRS
Refinement	
Model composition	
Non-hydrogen atoms	17515
Protein residues	2239
Resolution<br>FSC map vs. model@0.143[b]	3.3
RMS deviations	
Bond lengths (Å)	0.004
Bond angles (Å)	0.679
Validation	
Molprobity score/Percentile	1.4
Clashscore/Percentile	3.19
Rotamer outliers (%)	0
Ramachandran plot	
% favored	95.84
% allowed	4.12
% outliers	0.05

[b]According to the map vs. model correlation coefficient defined in [2]

Supplementary Table 5. iBAQ (intensity based absolute quantification)[3] values of Gid subunits extracted from mass spectrometry datasets. Measurements for each condition were performed in triplicates.

	YPD			YPE			Carbon Recovery		
	iBAQ 01	iBAQ 02	iBAQ 03	iBAQ 01	iBAQ 02	iBAQ 03	iBAQ 01	iBAQ 02	iBAQ 03
Gid1	94498	69984	60015	16606	17383	18574	85177	142930	78381
Gid2	54150	40248	40493	9201.9	9014.6	8911.3	55578	89842	56183
Gid5	80736	67929	64910	15143	14434	15222	75866	99240	72213
Gid7	658.91	279.6	277.65	1503.6	1650.2	1550.7	2284.5	3537.6	962.41
Gid8	75282	64118	57983	8999.9	9598.1	6612.8	73550	97735	67427
Gid9	55561	43937	38244	6913.6	6958.4	7940.4	53954	83935	52944
Gid4	163990	135800	115740	10501	9493.8	9387	262110	313950	223030
Gid12	33503	25113	26117	9839.9	10783	10999	2023.9	5187.1	1902

Supplementary Table 6. Summary of raw datasets of MS which uploaded into PRIDE database.

Raw file name	Sample type	Figure
20200923_TTOF01_LC12_17_SA_Orbi3661_Sample01_C1_1_2465.d	wt-YPD, untagged	Fig. 1a
20200923_TTOF01_LC12_17_SA_Orbi3661_Sample02_C2_1_2466.d	wt-YPD, untagged	Fig. 1a
20200923_TTOF01_LC12_17_SA_Orbi3661_Sample03_C3_1_2467.d	wt-YPD, untagged	Fig. 1a
20200923_TTOF01_LC12_17_SA_Orbi3661_Sample04_C4_1_2469.d	YPD, bait: Gid12-3xFLAG	Fig. 1a
20200923_TTOF01_LC12_17_SA_Orbi3661_Sample05_C5_1_2470.d	YPD, bait: Gid12-3xFLAG	Fig. 1a
20200923_TTOF01_LC12_17_SA_Orbi3661_Sample06_C6_1_2471.d	YPD, bait: Gid12-3xFLAG	Fig. 1a
20201216_TTOF01_LC12_17_SA_Orbi3743_Sample04_D4_1_2985.d	YPD, bait: Gid4-3xHA	Fig. 7c, Suppl. Table 5
20201216_TTOF01_LC12_17_SA_Orbi3743_Sample05_D5_1_2986.d	YPD, bait: Gid4-3xHA	Fig. 7c, Suppl. Table 5
20201216_TTOF01_LC12_17_SA_Orbi3743_Sample12_E3_1_2995.d	YPD, bait: Gid4-3xHA	Fig. 7c, Suppl. Table 5
20201216_TTOF01_LC12_17_SA_Orbi3743_Sample16_E7_1_3008.d	Recovery, bait: Gid4-3xHA	Fig. 7c, Suppl. Table 5
20201216_TTOF01_LC12_17_SA_Orbi3743_Sample17_E8_1_3009.d	Recovery, bait: Gid4-3xHA	Fig. 7c, Suppl. Table 5
20201216_TTOF01_LC12_17_SA_Orbi3743_Sample18_E9_1_3010.d	Recovery, bait: Gid4-3xHA	Fig. 7c, Suppl. Table 5
20210120_TTOF01_LC12_17_SA_Orbi3753_Sample04_B4_1_3123.d	YPE, bait: Gid4-3xHA	Fig. 7c, Suppl. Table 5
20210120_TTOF01_LC12_17_SA_Orbi3753_Sample05_B5_1_3124.d	YPE, bait: Gid4-3xHA	Fig. 7c, Suppl. Table 5
20210120_TTOF01_LC12_17_SA_Orbi3753_Sample06_B6_1_3125.d	YPE, bait: Gid4-3xHA	Fig. 7c, Suppl. Table 5

20220120_EXPLcore_BaSt_LC12_16_SA_Orbi4087_Sample1.raw	YPD, wt, bait: Gid8-3xHA	Suppl. Fig. 1b
20220120_EXPLcore_BaSt_LC12_16_SA_Orbi4087_Sample1_2.raw	YPD, wt, bait: Gid8-3xHA	Suppl. Fig. 1b
20220120_EXPLcore_BaSt_LC12_16_SA_Orbi4087_Sample1_3.raw	YPD, wt, bait: Gid8-3xHA	Suppl. Fig. 1b
20220120_EXPLcore_BaSt_LC12_16_SA_Orbi4087_Sample2_1.raw	YPD, *gid4*Δ strain, bait: Gid8-3xHA	Suppl. Fig. 1b
20220120_EXPLcore_BaSt_LC12_16_SA_Orbi4087_Sample2_2.raw	YPD, *gid4*Δ strain, bait: Gid8-3xHA	Suppl. Fig. 1b
20220120_EXPLcore_BaSt_LC12_16_SA_Orbi4087_Sample2_3.raw	YPD, *gid4*Δ strain, bait: Gid8-3xHA	Suppl. Fig. 1b
20220122_EXPL1_TuVu_SA_M650_Yeast_global_1.raw	WT_YPD	Suppl. Fig. 8b, 9
20220122_EXPL1_TuVu_SA_M650_Yeast_global_2.raw	WT_YPE	Suppl. Fig. 8b, 9
20220122_EXPL1_TuVu_SA_M650_Yeast_global_3.raw	WT_REC_30min	Suppl. Fig. 8b, 9
20220122_EXPL1_TuVu_SA_M650_Yeast_global_4.raw	WT_REC_2hours	Suppl. Fig. 8b, 9
20220122_EXPL1_TuVu_SA_M650_Yeast_global_5.raw	WT_REC_4hours	Suppl. Fig. 8b, 9
20220122_EXPL1_TuVu_SA_M650_Yeast_global_6.raw	O.E.*GID12*_YPD	Suppl. Fig. 8b, 9
20220122_EXPL1_TuVu_SA_M650_Yeast_global_7raw	O.E.*GID12*_YPE	Suppl. Fig. 8b, 9
20220122_EXPL1_TuVu_SA_M650_Yeast_global_8.raw	O.E.*GID12*_REC_30min	Suppl. Fig. 8b, 9
20220122_EXPL1_TuVu_SA_M650_Yeast_global_9.raw	O.E.*GID12*_REC_2hours	Suppl. Fig. 8b, 9
20220122_EXPL1_TuVu_SA_M650_Yeast_global_10.raw	O.E.*GID12*_REC_4hours	Suppl. Fig. 8b, 9
20220122_EXPL1_TuVu_SA_M650_Yeast_global_11.raw	WT_YPD	Suppl. Fig. 8b, 9
20220122_EXPL1_TuVu_SA_M650_Yeast_global_12.raw	WT_YPE	Suppl. Fig. 8b, 9
20220122_EXPL1_TuVu_SA_M650_Yeast_global_13.raw	WT_REC_30min	Suppl. Fig. 8b, 9
20220122_EXPL1_TuVu_SA_M650_Yeast_global_14.raw	WT_REC_2hours	Suppl. Fig. 8b, 9
20220122_EXPL1_TuVu_SA_M650_Yeast_global_15.raw	WT_REC_4hours	Suppl. Fig. 8b, 9
20220122_EXPL1_TuVu_SA_M650_Yeast_global_16.raw	O.E.*GID12*_YPD	Suppl. Fig. 8b, 9
20220122_EXPL1_TuVu_SA_M650_Yeast_global_17raw	O.E.*GID12*_YPE	Suppl. Fig. 8b, 9
20220122_EXPL1_TuVu_SA_M650_Yeast_global_18.raw	O.E.*GID12*_REC_30min	Suppl. Fig. 8b, 9
20220122_EXPL1_TuVu_SA_M650_Yeast_global_19.raw	O.E.*GID12*_REC_2hours	Suppl. Fig. 8b, 9
20220122_EXPL1_TuVu_SA_M650_Yeast_global_20.raw	O.E.*GID12*_REC_4hours	Suppl. Fig. 8b, 9
20220122_EXPL1_TuVu_SA_M650_Yeast_global_21.raw	WT_YPD	Suppl. Fig. 8b, 9
20220122_EXPL1_TuVu_SA_M650_Yeast_global_22.raw	WT_YPE	Suppl. Fig. 8b, 9
20220122_EXPL1_TuVu_SA_M650_Yeast_global_23.raw	WT_REC_30min	Suppl. Fig. 8b, 9

20220122_EXPL1_TuVu_SA_M650_Yeast_global_24.raw	WT_REC_2hours	Suppl. Fig. 8b, 9
20220122_EXPL1_TuVu_SA_M650_Yeast_global_25.raw	WT_REC_4hours	Suppl. Fig. 8b, 9
20220122_EXPL1_TuVu_SA_M650_Yeast_global_26.raw	O.E.*GID12*_YPD	Suppl. Fig. 8b, 9
20220122_EXPL1_TuVu_SA_M650_Yeast_global_27raw	O.E.*GID12*_YPE	Suppl. Fig. 8b, 9
20220122_EXPL1_TuVu_SA_M650_Yeast_global_28.raw	O.E.*GID12*_REC_30min	Suppl. Fig. 8b, 9
20220122_EXPL1_TuVu_SA_M650_Yeast_global_29.raw	O.E.*GID12*_REC_2hours	Suppl. Fig. 8b, 9
20220122_EXPL1_TuVu_SA_M650_Yeast_global_30.raw	O.E.*GID12*_REC_4hours	Suppl. Fig. 8b, 9
20211130_EXPL3_OzKa_SA_yeast_proteome_1.raw	WT_YPD	Suppl. Fig. 8b, 9
20211130_EXPL3_OzKa_SA_yeast_proteome_2.raw	WT_YPE	Suppl. Fig. 8b, 9
20211130_EXPL3_OzKa_SA_yeast_proteome_3.raw	WT_REC_30min	Suppl. Fig. 8b, 9
20211130_EXPL3_OzKa_SA_yeast_proteome_4.raw	WT_REC_2hours	Suppl. Fig. 8b, 9
20211130_EXPL3_OzKa_SA_yeast_proteome_5.raw	WT_REC_4hours	Suppl. Fig. 8b, 9
20211130_EXPL3_OzKa_SA_yeast_proteome_11.raw	*gid12*Δ_YPD	Suppl. Fig. 8b, 9
20211130_EXPL3_OzKa_SA_yeast_proteome_12.raw	*gid12*Δ_YPE	Suppl. Fig. 8b, 9
20211130_EXPL3_OzKa_SA_yeast_proteome_13.raw	*gid12*Δ_REC_30min	Suppl. Fig. 8b, 9
20211130_EXPL3_OzKa_SA_yeast_proteome_14.raw	*gid12*Δ_REC_2hours	Suppl. Fig. 8b, 9
20211130_EXPL3_OzKa_SA_yeast_proteome_15.raw	*gid12*Δ_REC_4hours	Suppl. Fig. 8b, 9
20211130_EXPL3_OzKa_SA_yeast_proteome_16.raw	WT_YPD	Suppl. Fig. 8b, 9
20211130_EXPL3_OzKa_SA_yeast_proteome_17.raw	WT_YPE	Suppl. Fig. 8b, 9
20211130_EXPL3_OzKa_SA_yeast_proteome_18.raw	WT_REC_30min	Suppl. Fig. 8b, 9
20211130_EXPL3_OzKa_SA_yeast_proteome_19.raw	WT_REC_2hours	Suppl. Fig. 8b, 9
20211130_EXPL3_OzKa_SA_yeast_proteome_20.raw	WT_REC_4hours	Suppl. Fig. 8b, 9
20211130_EXPL3_OzKa_SA_yeast_proteome_26.raw	*gid12*Δ_YPD	Suppl. Fig. 8b, 9
20211130_EXPL3_OzKa_SA_yeast_proteome_27.raw	*gid12*Δ_YPE	Suppl. Fig. 8b, 9
20211130_EXPL3_OzKa_SA_yeast_proteome_28.raw	*gid12*Δ_REC_30min	Suppl. Fig. 8b, 9
20211130_EXPL3_OzKa_SA_yeast_proteome_29.raw	*gid12*Δ_REC_2hours	Suppl. Fig. 8b, 9
20211130_EXPL3_OzKa_SA_yeast_proteome_30.raw	*gid12*Δ_REC_4hours	Suppl. Fig. 8b, 9
20211130_EXPL3_OzKa_SA_yeast_proteome_31.raw	WT_YPD	Suppl. Fig. 8b, 9
20211130_EXPL3_OzKa_SA_yeast_proteome_32.raw	WT_YPE	Suppl. Fig. 8b, 9

20211130_EXPL3_OzKa_SA_yeast_proteome_33.raw	WT_REC_30min	Suppl. Fig. 8b, 9
20211130_EXPL3_OzKa_SA_yeast_proteome_34.raw	WT_REC_2hours	Suppl. Fig. 8b, 9
20211130_EXPL3_OzKa_SA_yeast_proteome_35.raw	WT_REC_4hours	Suppl. Fig. 8b, 9
20211130_EXPL3_OzKa_SA_yeast_proteome_41.raw	*gid12*Δ_YPD	Suppl. Fig. 8b, 9
20211130_EXPL3_OzKa_SA_yeast_proteome_42.raw	*gid12*Δ_YPE	Suppl. Fig. 8b, 9
20211130_EXPL3_OzKa_SA_yeast_proteome_43.raw	*gid12*Δ_REC_30min	Suppl. Fig. 8b, 9
20211130_EXPL3_OzKa_SA_yeast_proteome_44.raw	*gid12*Δ_REC_2hours	Suppl. Fig. 8b, 9
20211130_EXPL3_OzKa_SA_yeast_proteome_45.raw	*gid12*Δ_REC_4hours	Suppl. Fig. 8b, 9

Supplementary Table 7. T-statistic of unpaired two-tailed Student's t-test performed in supplementary Figure 9.

WT_YPD vs O.E.*GID12*_YPD	WT_YPE vs O.E.*GID12*_YPE	WT_REC_30min vs O.E.*GID12*_REC_30min	WT_REC_2hours vs O.E.*GID12*_REC_2hours	WT_REC_4hours vs O.E.*GID12*_REC_4hours	Genes
0.5620	-0.3027	0.6655	1.3584	2.9668	CPA1
-2.0837	2.3193	-6.9776	-12.5529	-68.8731	FBP1
-9.4366	1.8934	-3.2117	-8.1246	-24.5766	PCK1
-15.5370	-0.4420	-28.4919	-27.7942	-82.2378	MDH2
-2.2826	-5.4302	-15.9218	-9.6966	-33.2109	ICL1
-2.2333	1.6431	-0.1251	1.4977	-0.8471	ART2
-5.5907	0.9832	-0.1309	-2.5924	-1.0748	GID4
-1.4767	1.3471	0.4928	1.5667	1.3237	BLM10
-0.0439	-4.3556	-4.2203	-7.8269	-0.8199	ACS2
-0.8460	3.1470	-3.5624	-10.5940	-14.0383	ACS1
-5.3509	-5.1239	-9.1534	-21.9059	-13.6008	ARO10
-12.1047	-5.8828	-3.8714	-1.8771	-10.9213	GID12

WT_YPD vs *gid12*Δ_YPD	WT_YPE vs *gid12*Δ_YPE	WT_REC_30min vs *gid12*Δ_REC_30min	WT_REC_2hours vs *gid12*Δ_REC_2hours	WT_REC_4hours vs *gid12*Δ_REC_4hours	Genes
0.0264	1.5008	-0.1180	-0.6374	2.0985	CPA1
-0.8162	-1.3111	-1.0328	-3.1244	-0.8903	FBP1
0.4241	-0.8448	-1.2092	-2.7846	1.4557	PCK1
1.3304	-2.2195	-1.0219	-1.6262	-1.8605	MDH2
0.7214	-1.7829	-0.7692	-2.1958	-1.5437	ICL1
-1.5206	-0.2014	-0.7871	-1.7656	-3.0653	ART2
0.1399	-0.6421	-2.0907	-1.8456	-1.1909	GID4
2.4670	0.9734	0.2217	-0.0536	4.0830	BLM10
0.4067	-1.4548	-0.6966	-1.9653	-1.1634	ACS2

0.7076	1.0536	-0.8607	1.9346	1.9944	ACS1
-6.2718	-1.9735	-1.9972	-3.2572	-3.5083	ARO10
-0.5624	-1.4581	-1.9764	-1.4129	0.4402	GID11

Supplementary Table 8. Cohen's d effect size of unpaired two-tailed Student's t-test performed in supplementary Figure 9.

WT_YPD vs O.E.*GID12*_YPD	WT_YPE vs O.E.*GID12*_YPE	WT_REC_30min vs O.E.*GID12*_REC_30min	WT_REC_2hours vs O.E.*GID12*_REC_2hours	WT_REC_4hours vs O.E.*GID12*_REC_4hours	PG.Genes
0.4589	-0.2471	0.5434	1.1091	2.4223	CPA1
-1.7013	1.8937	-5.6972	-10.2494	-56.2346	FBP1
-7.7049	1.5459	-2.6223	-6.6337	-20.0667	PCK1
-12.6859	-0.3609	-23.2635	-22.6938	-67.1468	MDH2
-1.8637	-4.4337	-13.0001	-7.9172	-27.1165	ICL1
-1.8235	1.3415	-0.1021	1.2229	-0.6917	ART2
-4.5648	0.8028	-0.1069	-2.1167	-0.8776	GID4
-1.2057	1.0999	0.4024	1.2792	1.0808	BLM10
-0.0359	-3.5564	-3.4459	-6.3906	-0.6695	ACS2
-0.6907	2.5695	-2.9087	-8.6500	-11.4622	ACS1
-4.3690	-4.1837	-7.4737	-17.8861	-11.1050	ARO10
-9.8835	-4.8033	-3.1610	-1.5326	-8.9172	GID12

WT_YPD vs *gid12*Δ_YPD	WT_YPE vs *gid12*Δ_YPE	WT_REC_30min vs *gid12*Δ_REC_30min	WT_REC_2hours vs *gid12*Δ_REC_2hours	WT_REC_4hours vs *gid12*Δ_REC_4hours	PG.Genes
0.0215	1.2254	-0.0964	-0.5204	1.7134	CPA1
-0.6664	-1.0705	-0.8433	-2.5510	-0.7269	FBP1
0.3463	-0.6898	-0.9873	-2.2736	1.1886	PCK1
1.0863	-1.8122	-0.8344	-1.3278	-1.5191	MDH2
0.5890	-1.4557	-0.6281	-1.7928	-1.2604	ICL1
-1.2416	-0.1644	-0.6427	-1.4416	-2.5028	ART2

0.1142	-0.5243	-1.7071	-1.5069	-0.9724	GID4
2.0143	0.7948	0.1810	-0.0438	3.3338	BLM10
0.3320	-1.1878	-0.5688	-1.6046	-0.9499	ACS2
0.5777	0.8603	-0.7028	1.5796	1.6284	ACS1
-5.1209	-1.6114	-1.6307	-2.6595	-2.8645	ARO10
-0.4592	-1.1905	-1.6137	-1.1537	0.3594	GID11

Supplementary Table 9. 95 % confidence interval of unpaired two-tailed Student's t-test performed in supplementary Figure 9. (lb, lower bound, ub, upper bound)

WT_YPD vs O.E.*GID12*_YPD		WT_YPE vs O.E.*GID12*_YPE		WT_REC_30min vs O.E.*GID12*_REC_30min		WT_REC_2hours vs O.E.*GID12*_REC_2hours		WT_REC_4hours vs O.E.*GID12*_REC_4hours		Genes
lb	ub	lb	ub	lb	ub	lb	ub	lb	ub	
-0.2300	0.3443	-1.6828	1.3535	-0.2073	0.3135	-0.2225	0.5935	-0.0457	0.5268	CPA1
-4.5027	1.4417	-0.0389	0.3647	-1.6701	-0.5490	-5.1037	-2.6428	-4.7723	-4.3971	FBP1
-3.1580	-1.5917	-0.1040	0.5007	-0.9248	-0.0256	-2.6241	-1.0656	-3.9195	-3.0498	PCK1
-1.3794	-0.7977	-0.3411	0.2583	-2.4254	-1.9599	-2.9272	-2.1898	-2.5592	-2.3615	MDH2
-2.6960	0.2659	-0.2279	-0.0533	-0.5603	-0.3362	-1.9638	-0.7882	-3.2176	-2.5874	ICL1
-0.2250	0.0319	-0.0929	0.3001	-0.2992	0.2781	-0.0548	0.1817	-0.2175	0.1363	ART2
-5.2172	-0.6935	-2.7413	5.4984	-0.4034	0.3674	-1.8748	0.3527	-6.7207	4.0137	GID4
-0.4168	0.1525	-0.1426	0.3871	-0.1168	0.1651	-0.1343	0.3022	-0.0908	0.1890	BLM10
-0.0835	0.0809	-0.4614	-0.1011	-0.5082	-0.0849	-0.3063	-0.1377	-0.2559	0.1730	ACS2
-2.3428	1.4142	0.0095	0.5270	-0.4275	-0.0521	-1.0424	-0.6091	-1.2046	-0.8067	ACS1
-0.7476	-0.1920	-0.9408	-0.2788	-0.6852	-0.3661	-0.9454	-0.7322	-1.1428	-0.6071	ARO10
-4.2007	-2.1795	-5.0408	-0.9323	-4.4102	0.0631	-7.9015	3.0862	-4.2907	-2.1449	GID12

WT_YPD vs *gid12*Δ_YPD	WT_YPE vs *gid12*Δ_YPE	WT_REC_30min vs *gid12*Δ_REC_30min	WT_REC_2hours vs *gid12*Δ_REC_2hours	WT_REC_4hours vs *gid12*Δ_REC_4hours	Genes

lb	ub	lb	ub	lb	ub	lb	ub	lb	ub	
-0.4190	0.4248	-0.7260	2.4280	-0.4673	0.4292	-0.3826	0.2782	-0.0656	0.3893	CPA1
-5.2142	2.9006	-0.6238	0.3083	-2.0672	1.2602	-2.1102	0.2548	-1.5184	0.8698	FBP1
-2.2226	2.9969	-0.4526	0.2902	-1.0369	0.4268	-1.5292	0.2680	-0.3354	0.9242	PCK1
-0.1128	0.3197	-1.5877	0.3636	-3.0876	1.7438	-2.3869	0.7451	-1.1421	0.2615	MDH2
-4.2637	6.6982	-1.7863	0.7267	-3.4657	2.4090	-4.4316	1.3249	-0.9451	0.2719	ICL1
-0.3546	0.1084	-0.3266	0.2848	-0.3679	0.2061	-0.6417	0.1963	-0.3875	0.0530	ART2
-3.0104	3.3206	-5.1306	3.5023	-1.8401	0.3167	-2.8133	0.9182	-3.0441	1.2170	GID4
-0.0596	0.3947	-0.0634	0.1318	-0.2021	0.2359	-0.0800	0.0770	0.0246	0.1363	BLM10
-0.3575	0.4445	-1.3100	0.5825	-1.7342	1.2441	-1.5597	0.5596	-0.5851	0.3151	ACS2
-4.0900	5.7579	-0.2382	0.4649	-0.3734	0.1968	-0.2314	0.6901	-0.2393	0.6831	ACS1
-2.1369	-0.8076	-1.4078	0.4001	-1.9681	0.6823	-2.3558	0.1548	-1.7536	-0.1330	ARO10
-1.9473	1.3181	-0.5366	0.2011	-1.7805	0.3919	-1.7741	0.6634	-1.7381	2.3794	GID11

Supplementary References

1 Rosenthal, P. B. & Henderson, R. Optimal determination of particle orientation, absolute hand, and contrast loss in single-particle electron cryomicroscopy. *J Mol Biol* **333**, 721-745, doi:10.1016/j.jmb.2003.07.013 (2003).

2 Afonine, P. V. *et al.* Real-space refinement in PHENIX for cryo-EM and crystallography. *Acta Crystallogr D Struct Biol* **74**, 531-544, doi:10.1107/s2059798318006551 (2018).

3 Schwanhäusser, B. *et al.* Global quantification of mammalian gene expression control. *Nature* **473**, 337-342, doi:10.1038/nature10098 (2011).
