# Supplementary material for: Cryo-EM structures of Gid12-bound GID E3 reveal steric blockade as a mechanism inhibiting substrate ubiquitylation
Source: Nat Commun. 2022 Jun 1;13:3041. doi: 10.1038/s41467-022-30803-9 (PMC9160049; doi:10.1038/s41467-022-30803-9)
Supplement: Supplementary file 3 — Reporting Summary [file 41467_2022_30803_MOESM3_ESM.pdf]

nature portfolio

Corresponding author(s): Brenda A. Schulman

Last updated by author(s): 3 May 2022

Reporting Summary

Nature Portfolio wishes to improve the reproducibility of the work that we publish. This form provides structure for consistency and transparency in reporting. For further information on Nature Portfolio policies, see our Editorial Policies and the Editorial Policy Checklist.

Statistics

For all statistical analyses, confirm that the following items are present in the figure legend, table legend, main text, or Methods section.

n/a	Confirmed	
☐	☒	The exact sample size (*n*) for each experimental group/condition, given as a discrete number and unit of measurement
☐	☒	A statement on whether measurements were taken from distinct samples or whether the same sample was measured repeatedly
☐	☒	The statistical test(s) used AND whether they are one- or two-sided<br>*Only common tests should be described solely by name; describe more complex techniques in the Methods section.*
☒	☐	A description of all covariates tested
☒	☐	A description of any assumptions or corrections, such as tests of normality and adjustment for multiple comparisons
☐	☒	A full description of the statistical parameters including central tendency (e.g. means) or other basic estimates (e.g. regression coefficient) AND variation (e.g. standard deviation) or associated estimates of uncertainty (e.g. confidence intervals)
☐	☒	For null hypothesis testing, the test statistic (e.g. *F*, *t*, *r*) with confidence intervals, effect sizes, degrees of freedom and *P* value noted<br>*Give P values as exact values whenever suitable.*
☒	☐	For Bayesian analysis, information on the choice of priors and Markov chain Monte Carlo settings
☒	☐	For hierarchical and complex designs, identification of the appropriate level for tests and full reporting of outcomes
☒	☐	Estimates of effect sizes (e.g. Cohen's *d*, Pearson's *r*), indicating how they were calculated

Our web collection on statistics for biologists contains articles on many of the points above.

Software and code

Policy information about availability of computer code

Data collection	Typhoon™ FLA 7000 biomolecular imager; AmershamTM Imager 600; OtofControl 6.2; EPU-2; SerialEM 3.8;Dark room; Orbitrap Exploris 480
Data analysis	Relion 3.1; PHENIX software suite; Chimera1.11.2; ChimeraX1.2 and PyMol-v 1.8.2; Coot0.8.9.1, CCP4-7.0, Perseus software platform (version 1.6.15.0); MaxQuant computational platform (version 1.6.17.0); Spectronaut version 15; Python version 3.5.5

For manuscripts utilizing custom algorithms or software that are central to the research but not yet described in published literature, software must be made available to editors and reviewers. We strongly encourage code deposition in a community repository (e.g. GitHub). See the Nature Portfolio guidelines for submitting code & software for further information.

Data

Policy information about availability of data

All manuscripts must include a data availability statement. This statement should provide the following information, where applicable:

- Accession codes, unique identifiers, or web links for publicly available datasets
- A description of any restrictions on data availability
- For clinical datasets or third party data, please ensure that the statement adheres to our policy

The PDB and EM maps will be available from the RCSB and EMDB upon publication as follows: Gid12-SRS, EMD-32830 [https://www.ebi.ac.uk/pdbe/entry/emdb/EMD-32830], PDB ID: 7WUG [https://doi.org/10.2210/pdb7WUG/pdb]; Gid12-GIDSR4, EMD-32831 [https://www.ebi.ac.uk/pdbe/entry/emdb/EMD-32831]; Gid12-Chelator-GIDSR4, EMD-32833 [https://www.ebi.ac.uk/pdbe/entry/emdb/EMD-32833]; Gid12-Cage-GIDSR4, EMD-32835 [https://www.ebi.ac.uk/pdbe/entry/emdb/EMD-32835]; Cage-GIDSR4, EMD-32834 [https://www.ebi.ac.uk/pdbe/entry/emdb/EMD-32834]; Chelator-GIDSR4-Mdh2, EMD-14323 [https://www.ebi.ac.uk/pdbe/entry/emdb/EMD-14323]; Endogenous Cage-GIDAnt, EMD-14338 [https://www.ebi.ac.uk/pdbe/entry/emdb/EMD-14338]; Cage-GIDSR4-Fbp1, EMD-14324 [https://www.ebi.ac.uk/pdbe/entry/emdb/EMD-14324]; Proteomics data of both the interactomes and the total proteome data will be available upon publication

from the ProteomeXchange via the Pride database with the data set identifier PXD028579 [http://proteomecentral.proteomexchange.org/cgi/GetDataset?ID=PXD028579] (interactome) (username: reviewer_pxd028579@ebi.ac.uk and password: 4QjjFS8w) and PXD031713 [http://proteomecentral.proteomexchange.org/cgi/GetDataset?ID=PXD031713] (total proteome) (username: reviewer_pxd031713@ebi.ac.uk and password: msghv4mn) (Supplementary Table 6). Source data are provided with this paper.

Field-specific reporting

Please select the one below that is the best fit for your research. If you are not sure, read the appropriate sections before making your selection.

- [x] Life sciences
- [ ] Behavioural & social sciences
- [ ] Ecological, evolutionary & environmental sciences

For a reference copy of the document with all sections, see nature.com/documents/nr-reporting-summary-flat.pdf

Life sciences study design

All studies must disclose on these points even when the disclosure is negative.

Sample size	Sample size is indicated in the figure legend for each experiments. No sample size calculation was done either for in vivo or in vitro studies. For IP-MS assay, n=3 samples per group is sufficient to detect meaningful biological differences with good reproducibility. For activity assay, pulldown assay, ubiquitin discharge assay, substrate receptor exchange assay, in vivo substrate receptor and substrate stability assay, GID subunits expression profile assay, results of three independent biological replicates were used.
Data exclusions	No data were excluded from the analyses
Replication	Data shown are representative results of three independent experiments with similar results. Consistent difference (or no difference) was observed, as indicated by the average value of each group. All attempts at replication were successful.
Randomization	Samples were handled and processed identically. For IP-MS assays, LC-MS data was acquired in a randomized order.
Blinding	Data were collected by the same person carrying out the expriments. Authors worked individually and collaboratively on the project. Therefore, the data were not collected in a blinded manner.

Reporting for specific materials, systems and methods

We require information from authors about some types of materials, experimental systems and methods used in many studies. Here, indicate whether each material, system or method listed is relevant to your study. If you are not sure if a list item applies to your research, read the appropriate section before selecting a response.

Materials & experimental systems

n/a	Involved in the study	
[ ]	[x]	Antibodies
[ ]	[x]	Eukaryotic cell lines
[x]	[ ]	Palaeontology and archaeology
[x]	[ ]	Animals and other organisms
[x]	[ ]	Human research participants
[x]	[ ]	Clinical data
[x]	[ ]	Dual use research of concern

Methods

n/a	Involved in the study	
[x]	[ ]	ChIP-seq
[x]	[ ]	Flow cytometry
[x]	[ ]	MRI-based neuroimaging

Antibodies

Antibodies used	HA-Tag Antibody (F-7) (Santa Cruz Biotechnology, sc-7392), 1:5000 dilution; Anti-cMyc (9E10) (Santa Cruz Biotechnology, sc-40),1:5000 dilution ; Anti-FLAG M2 monoclonal antibody (Sigma, F1804), 1:5000 dilution; PGK1 monoclonal antibody(22C5D8), Invitrogen, Catalog #459250, 1:50,000 dilution.
Validation	All the primary antibodies used in this study have been validated in various species for WB by the manufactures. The primary antibodies of anti-FLAG, anti-Myc and anti-HA were validated with tagged and untagged wild type yeast cell lysates, only tagged yeast strains have signal. The primary antibody of anti-PGK1 was validated with wild yeast strain.<br><br>From manufacturer information:<br>Anti-HA-Tag Antibody (F-7) is a mouse monoclonal IgG2a κ HA-Tag antibody, cited in 2,643 publications, provided at 200 µg/ml; Specific to epitope mapping within an internal region of the the influenza hemagglutinin (HA) protein; HA-Tag Antibody (F-7) is recommended for detection of proteins containing the HA tag by WB, IP, IF, FCM and ELISA.<br><br>Anti-Myc/c-Myc Antibody (9E10) is a mouse monoclonal IgG1 κ Myc/c-Myc antibody, cited in 9,111 publications, provided at 200 µg/ml; Raised against an epitope corresponding to amino acids 408-439 within the C-terminal domain of c-Myc of human origin;

This Myc antibody is recommended for detection of c-Myc p67 and c-Myc tagged fusion proteins of mouse, rat, human, monkey, feline and canine origin by WB, IP, IF, IHC(P), FCM and ELISA; non cross-reactive with N-Myc or L-Myc proteins. Widely used in combination with eukaryotic expression vectors encoding proteins with c-Myc (amino acids 408-439) epitope tag.

The Anti-FLAG M2 mouse, affinity purified monoclonal antibody binds to fusion proteins containing a FLAG peptide sequence. The antibody recognizes the FLAG peptide sequence at the N-terminus, Met-N-terminus,C-terminus, and internal sites of the fusion protein; Method of purification - Affinity tag purification; Specificity (Binding site):N-Asp-Tyr-Lys-Asp-Asp-Asp-Asp-Lys-C; Immunogen: FLAG; peptide sequence DYKDDDDK; Application :For highly sensitive and specific detection of FLAG fusion proteins by immunoblotting, immunoprecipitation (IP) , immunohistochemisty, immunofluorescence and immunocyotchemistry. Optimized for single banded detection of FLAG fusion proteins in mammalian, plant, and bacterial expression systems. Western Blotting and EIA.

PGK1 Monoclonal Antibody, cited in 155 publications, specially react to Yeast; Immunogen: Full-length native S. cerevisiae PGK protein (purified); Applications: Western Blot (WB) and others; This product reacts with Saccharomyces cerevisiae PGK1 - predicted molecular weight: 45 kDa.

Eukaryotic cell lines

Policy information about cell lines

Cell line source(s)	S. cerevisiae S288C with genotype MATa his3Δ1 leu2Δ0 met15Δ0 ura3Δ0 was used in this study. The strain was obtained form Euroscarf (Cat#Y00000)
Authentication	Authentication of cell lines that were not generated in this study was done on the basis of the expected phenotype of each specific cell line.
Mycoplasma contamination	No testing necessary since only yeast was used.
Commonly misidentified lines (See ICLAC register)	No commonly misidentified cell lines were used.
