# Supplementary material for: Cryo-EM structures of Gid12-bound GID E3 reveal steric blockade as a mechanism inhibiting substrate ubiquitylation
Source: Nat Commun. 2022 Jun 1;13:3041. doi: 10.1038/s41467-022-30803-9 (PMC9160049; doi:10.1038/s41467-022-30803-9)
Supplement: Supplementary file 2 — Peer Review File [file 41467_2022_30803_MOESM2_ESM.pdf]

nature portfolio

Peer Review File

Cryo-EM structures of Gid12-bound GID E3 reveal steric blockade as a mechanism inhibiting substrate ubiquitylation

Open Access This file is licensed under a Creative Commons Attribution 4.0 International License, which permits use, sharing, adaptation, distribution and reproduction in any medium or format, as long as you give appropriate credit to the original author(s) and the source, provide a link to the Creative Commons license, and indicate if changes were made. In the cases where the authors are anonymous, such as is the case for the reports of anonymous peer reviewers, author attribution should be to 'Anonymous Referee' followed by a clear attribution to the source work. The images or other third party material in this file are included in the article's Creative Commons license, unless indicated otherwise in a credit line to the material. If material is not included in the article's Creative Commons license and your intended use is not permitted by statutory regulation or exceeds the permitted use, you will need to obtain permission directly from the copyright holder. To view a copy of this license, visit http://creativecommons.org/licenses/by/4.0/.

REVIEWER COMMENTS

Reviewer #1 (Remarks to the Author):

This manuscript addresses the potential role of Gid12 as a negative regulator of the yeast GID ubiquitin ligase complex in targeting the substrate Fbp1 for degradation. The authors used a combination of cryo-EM and biochemical and genetic assays to gain detailed mechanistic and structural insights into the assembly and function of Gid12 with the known core GID complex. The authors demonstrated that Gid12 directly interacts with the substrate receptor Gid4 and the scaffold protein Gid5, and reshapes the degron binding site in Gid4. Based on a low-resolution cryo-EM map, they also proposed that Gid12 binding would limit the encapsulation of the Fbp1 tetramer in a supramolecular chelate GID complex structure. These structural observations are supported by a set of in vitro and in vivo assays to show the impact of Gid12 on substrate ubiquitination and protein level. The structure determination and subsequent functional studies are well performed and clearly described. Overall, these findings substantially advance the molecular understanding of regulatory mechanism of substrate recruitment by the GID ligase complex. These are important advances that certainly merit publication in Nature Communication.

Before publication, the following minor concerns and suggestions should be addressed.

1) Impact of Gid12 on the protein level of other substrates especially Mdh2 should be addressed. Recent studies have shown that the substrate chelating factor Gid7 is required for the degradation of Fbp1 but dispensable for the turnover of other substrates including Mdh2. Here the authors proposed that Gid12 inhibits Fbp1 ubiquitination by blocking its access to the central oval of the chelator GID that depends on Gid7. However, Fig7c shows that overexpression of Gid12 has even more effect on the stability of Mdh2 than Fbp1.

2) Physiological relevance of the Cage-GIDSR4 needs to be clarified. Fig. S5 indicates that Cage-GID only represents a minor species (9.2%) based on the Cryo-EM data. The authors also indicated that they were unable to isolate Cage-GID in vitro. It is thus unclear to the reviewer that whether this conformation is just an artifact.

3) In Fig. 2a and b, it appears that Gid12 also consists of several helixes in addition to the beta-propeller. But it is not discussed in the text.

4) Blades numbers should be labeled in Fig. 2b.

5) Fig. 5c is confusing. Can it be shown in a 3D representation as in Fig. 5a and b?

6) Fig. 7c. Why the time course for Fbp1 and Mdh2 are from 0-2 hr, while it is 0-4 hr for Ic1 and Pck1? It should be consistent for every substrate.

Reviewer #2 (Remarks to the Author):

Dear Editors of Nature Communication,

The manuscript "Cryo-EM structures of Gid12-bound GID E3 reveal steric blockade as a mechanism inhibiting substrate ubiquitylation" describes the function of the protein Ipf1 (YDL176W) as a potential regulator of GID-E3 ligase activity. Ipf1 (in the following publication named Gid12) was previously shown to interact with different subunits of the Gid-complex [1; 2; 3]. The Gid-complex is an evolutionary conserved ubiquitin-ligase

complex that targets key enzymes of gluconeogenesis for glucose induced degradation in yeast and exists with different subunit compositions. Gid4 is one of several substrate recognition factors that bind yeast substrates via their N-terminal proline residues to allow polyubiquitination and subsequent proteasomal degradation. Gid4 levels strongly vary depending on the carbon source, thus allowing substrate degradation depending on Gid4 levels. Gid4 is almost absent in cells grown on YPEthanol (ethanol as C-source) and is strongly upregulated on YPD (glucose as C-source), allowing substrate degradation depending on Gid4 levels [4; 5]. Other layers of GID-complex regulation are currently unknown.

The current study from the Schulman lab uses co-immunoprecipitation methods to isolate endogenous Ipf1 from cell lysates under glycolytic conditions. The authors find the core Gid-complex subunits enriched in the precipitate, confirming previous data that Ipf1 binds Gid-complex subunits. Co-expression of Gid12 with other Gid-subunits and subsequent Cryo-EM, allows insights into structural composition and suggests direct binding to the subunits Gid4 and Gid5 within the complex. Additionally, the structural data indicates that Gid12 binding induces conformational changes of loop structures at the entrance of the substrate binding tunnel within Gid4 and partially occupies space between Gid4 and the Gid2/9 RING domains. The authors suggest that substrate accessibility could be compromised by Gid12 binding. Additionally, the authors describe a previously unrecognized 5 MDa Gid-complex assembly that seems to consists of three Chelator-GidSR assemblies that was named the Cage-GIDSR4 assembly. This complex assembles with or without Gid12 in vitro. Biochemical assays suggest, that Gid12 blocks Gid4 assembly into the anticipatory Gid-complex and inhibits polyubiquitination of Fbp1, Mdh2 and Icl1 in vitro. In vivo, overexpression of Gid12 results in stabilization of gluconeogenic key enzymes upon glucose replenishment. Gid12 is abundant when cells are grown on YPD, on YPE its levels drop and increase upon glucose recovery. The authors immunoprecipitate Gid4 from lysates generated from yeast cells grown on different carbon sources and find Gid12 co-precipitating when cells are grown on YPD and on YPE but not after glucose recovery.

In summary, the manuscript provides interesting novel insights into the function of Ipf1 in the regulation of Gid-complex activity. However, more detailed information of how Gid12 regulation works is required. Also at this stage it remains completely unclear if the CAGE-assembly described in the manuscript exists in vivo and which function it may have.The following points should be addressed prior to publication.
Major concerns:
1. In Figure 5 the authors describe a 5 MDa Gid-complex assembly that they name the Cage-GID complex. Unfortunately, the authors neither provide in vivo data to convince the reader of its existence in cells, nor do they provide experimental data of a possible function. Furthermore, the authors show that the assembly is independent of Gid12. The focus of the manuscript is put on the function of Gid12, in order to keep the focus I recommend to remove the entire Figure 5 from the manuscript. A minimal requirement is to purify the complex from lysates and to determine the molecular mass of the different sub-compositions by gel chromatography or glycerol gradient centrifugation experiments.
2. The authors convincingly show that Ipf1/Gid12 binds to the Gid-complex and suggest that it prevents Gid4 incorporation into the complex, and thus substrate polyubiquitination and degradation. The data is consistent and supports such theory. However, it remains unclear how Gid12 is released from the complex and if Gid12 functions for other substrate recognition factors as well.

Questions arrising:
- Is Ipf1 a general factor that blocks docking of substrate recognition factors like Gid10 and Gid11? Does overexpression of GID12 prevent the incorporation of Gid10 into the complex and does Gid12 overexpression block degradation of e.g. Phm8 as well?

- Gid4 is a substrate recognition factor but also a substrate of the Gid-complex. Upon glucose replenishment Gid4 gets first upregulated and then degraded probably together with the substrate. Is Gid4 degradation also affected by Gid12 overexpression?

- Gid12 levels differ dependent on the growth conditions (Figure 7a). Cells grown on YPD and 1 h after glucose supplementation display high levels of Gid12, while grown on non-fermentable carbon sources Gid12 is present at low levels. How is the level of Gid12 regulated? Is there transcriptional regulation? Is Gid12 turned over depending on the carbon source and might this be a way to activate the complex?

- Figure 1B and Figure 2 suggest that Gid12 primarily associates with the Gid-complex via the subunits Gid4 and Gid5. To test whether this is also the case in vivo, Co-IPs should be performed with a tagged subunit of the Gid-complex and Gid12-6*HA in WT and in gid4 deletion strains.

- In Figure 7b the authors precipitate Gid4 and quantify Co-IPed Gid2 and Gid12. Surprisingly, after Co-IP the level of Gid4 and Gid12 is similar to the level of Gid2 when grown on YPE. This suggests, that the entire remaining low levels of Gid4 and Gid12 under these conditions are trapped in the complex in an inactive state. It appears that Gid12 not only prevents unbound Gid4 from associating with the GidAnt, but also traps remaining Gid4 within the primed but inactivated complex. Glucose induced removal of Gid12 from the primed inactive complex would guarantee a rapid first response to glucose supplementation. To better assess this possibility a Gid-complex subunit like Gid1 would be better suited for pull down experiments because the protein level does not fluctuate dependent on growth conditions (like for Gid4). Most of the Gid4 protein pulled down in Fig7b is protein not bound to the complex.
Minor points:
1. Please use either ubiquitylation or ubiquitination. P.30 Figure legend 6c change ubiquitination to ubiquitylation.
2. Throughout the whole manuscript the term carbon starvation is used. I find the term misleading because ethanol is also a C-source. Please change the term e.g. use the term non-fermentable carbon source or glucose retraction
3. P9 line9: change the sentence "Use of a tagged Gid4 version therefore allows to distinguish between Gid4 assembled into...and Gid4 free in solution.
4. P10 heading. ...stabilizes Fbp1 protein in vivo (not the levels)
5. P10 line 16: The sentence ' this is consistent with prior.... I don't understand why a tag is consistent with protein levels, please explain
6. P10 line 20 change carbon stress to growth on a non-fermentable carbon source
7. P10 line 23 is restored should be was restored
8. P10 line 25 delete , as might be expected for a modulatory protein
9. P10 line 26 insert '...Gid12 in complex with Gid4 by co-immunoprecipitation
10. P11 line 12 ...had the capacity to inhibit...
11. P12 line 6 towards FBP1
12. P12 line13 by the effect
13. P12 line 20 ... Gid12 may inhibit_ GID E3....
14. P13 line 2 delete enzyme TCA cycle enzymes.
15. P14 ...by centrifugation at 20000 rpm ...please mention rotor or use g instead
16. P17 might be my progressing cataract but some sections appear grey...please change to black
17. P23 in the acknowledgement delete one of the two 'for peptide synthesis'
18. Please cite Liu et al., 2020 on P 3 line 16

[1] N.J. Krogan, G. Cagney, H. Yu, G. Zhong, X. Guo, A. Ignatchenko, J. Li, S. Pu, N. Datta,

A.P. Tikuisis, T. Punna, J.M. Peregrin-Alvarez, M. Shales, X. Zhang, M. Davey, M.D. Robinson, A. Paccanaro, J.E. Bray, A. Sheung, B. Beattie, D.P. Richards, V. Canadien, A. Lalev, F. Mena, P. Wong, A. Starostine, M.M. Canete, J. Vlasblom, S. Wu, C. Orsi, S.R. Collins, S. Chandran, R. Haw, J.J. Rilstone, K. Gandi, N.J. Thompson, G. Musso, P. St Onge, S. Ghanny, M.H. Lam, G. Butland, A.M. Altaf-Ul, S. Kanaya, A. Shilatifard, E. O'Shea, J.S. Weissman, C.J. Ingles, T.R. Hughes, J. Parkinson, M. Gerstein, S.J. Wodak, A. Emili, and J.F. Greenblatt, Global landscape of protein complexes in the yeast Saccharomyces cerevisiae. Nature 440 (2006) 637-43.
[2] S. Pitre, F. Dehne, A. Chan, J. Cheetham, A. Duong, A. Emili, M. Gebbia, J. Greenblatt, M. Jessulat, N. Krogan, X. Luo, and A. Golshani, PIPE: a protein-protein interaction prediction engine based on the re-occurring short polypeptide sequences between known interacting protein pairs. BMC Bioinformatics 7 (2006) 365.
[3] R.I. Subbotin, and B.T. Chait, A pipeline for determining protein-protein interactions and proximities in the cellular milieu. Mol Cell Proteomics 13 (2014) 2824-35.
[4] O. Santt, T. Pfirrmann, B. Braun, J. Juretschke, P. Kimmig, H. Scheel, K. Hofmann, M. Thumm, and D.H. Wolf, The yeast GID complex, a novel ubiquitin ligase (E3) involved in the regulation of carbohydrate metabolism. Mol Biol Cell 19 (2008) 3323-33.
[5] R. Menssen, K. Bui, and D.H. Wolf, Regulation of the Gid ubiquitin ligase recognition subunit Gid4. FEBS Lett 592 (2018) 3286-3294.

Reviewer #3 (Remarks to the Author):

This paper describes the investigation of the role of the Gid12 protein in the regulation of the multi-subinit GID E3 ligase system primarily by cryo-EM based structural analysis of several GID protein assemblies. In particular, the authors report a high resolution structure of a 5 subunit Gid12- SRS complex and intermediate resolution structures of larger structures referred to as Gid12-GIDSR4, Gid12-Chelator-GIDSR4 and Gid12-Cage-GIDSR4. The first three structures build on recent previous structural analysis by the Schulman group of equivalent structures lacking the Gid12 protein, while the fourth represents a novel higher order complex containing three copies of Gid12-Chelator-GIDSR4. GID E3 ligases are interesting and relevant systems which have been substantially characterised in recent studies to which the Schulman group has substantially contributed. The structural analysis and biochemical measurements presented here indicate that Gid12 acts by sterically interfering with ability of GID E3 ligase complexes to engage productively with its Fbp1 substrate.
Overall I consider that this is a well conducted and valuable piece of work which contributes to a greater understanding of the Gid12 protein, the role of which, nevertheless, remains somewhat nebulous.
Detailed points:
1) The paper describes a range of GID complexes characterised by different combinations of subunits and symmetries with a range of different names made up of acronyms. For readers less familiar with the GID story I think it might help to provide a guide perhaps in the form of a table or set of cartoons to make it easier to keep track of what is going on.

2) Aside from the Gid12- SRS complex, the cryo-EM analysis of other larger Gid complexes described in this paper are of somewhat limited resolution. This is most likely a consequence of either compositional or conformational heterogeneity (or both). It would be interesting to learn to what extent this could be compensated for by local or multi body refinement or in the case of the symmetric complexes by symmetry expansion.

Reviewer comments and point-by-point responses in blue:

Reviewer #1 (Remarks to the Author):

This manuscript addresses the potential role of Gid12 as a negative regulator of the yeast GID ubiquitin ligase complex in targeting the substrate Fbp1 for degradation. The authors used a combination of cryo-EM and biochemical and genetic assays to gain detailed mechanistic and structural insights into the assembly and function of Gid12 with the known core GID complex. The authors demonstrated that Gid12 directly interacts with the substrate receptor Gid4 and the scaffold protein Gid5, and reshapes the degron binding site in Gid4. Based on a low-resolution cryo-EM map, they also proposed that Gid12 binding would limit the encapsulation of the Fbp1 tetramer in a supramolecular chelate GID complex structure. These structural observations are supported by a set of in vitro and in vivo assays to show the impact of Gid12 on substrate ubiquitination and protein level. The structure determination and subsequent functional studies are well performed and clearly described. Overall, these findings substantially advance the molecular understanding of regulatory mechanism of substrate recruitment by the GID ligase complex. These are important advances that certainly merit publication in Nature Communication.

We thank the reviewer for such kind comments! We are very pleased by the enthusiastic response!

Before publication, the following minor concerns and suggestions should be addressed.

1) Impact of Gid12 on the protein level of other substrates especially Mdh2 should be addressed. Recent studies have shown that the substrate chelating factor Gid7 is required for the degradation of Fbp1 but dispensable for the turnover of other substrates including Mdh2. Here the authors proposed that Gid12 inhibits Fbp1 ubiquitination by blocking its access to the central oval of the chelator GID that depends on Gid7. However, Fig7c shows that overexpression of Gid12 has even more effect on the stability of Mdh2 than Fbp1.

We addressed this question with several new experiments:

1) We tested effects of Gid12 on ubiquitylation of fluorescently-labeled model substrates wherein a lysine is flexibly tethered to substrate's Pro/N-degron sequence. Prior studies showed differences amongst substrate degrons: the Mdh2 degron motif is sufficient to confer robust GID^{SR4}-mediated polyubiquitylation of the model peptide substrate, whereas a peptide harboring the weaker Fbp1 degron is ubiquitylated to a lesser extent (for the Fbp1 protein, the weak degron of an individual protomer is overcome by tetramerization, which enables avid binding to higher-order Chelator- or Cage-GID^{SR4} assemblies). We now show that Gid12 swaps intrinsic preference of GID^{SR4} toward the different degrons. Gid12 inhibits ubiquitylation of the peptide with the Mdh2 degron, but potentiates ubiquitylation of that with the Fbp1 degron. These new data - shown in Fig. 6c - provide a biochemical explanation for why overexpression of Gid12 stabilizes Mdh2.

2) We obtained a cryo-EM map of Mdh2-bound Chelator-GID^{SR4}. The data show Mdh2 encapsulated in the center of the E3 complex. The new data, shown in Fig. 6d, suggest that as for Fbp1, Gid12 would obstruct proper placement of Mdh2 in the context of higher-order GID^{SR4} E3 assemblies.

3) We performed quantitative total proteome analyses with wild type yeast, a *gid12* deletion strain, and a strain overexpressing Gid12. Experiments were performed under glycolytic conditions and various times after replenishing glucose. We compared the levels of all reported substrates. While impact of *gid12* deletion is subtle, overexpression of Gid12

stabilizes all known Gid4-dependent substrates Fbp1, Mdh2, Icl1 and Pck1), and Aro10. These new data are reported in Supplementary Figs. 8b and 9.

2) Physiological relevance of the Cage-GIDSR4 needs to be clarified. Fig. S5 indicates that Cage-GID only represents a minor species (9.2%) based on the Cryo-EM data. The authors also indicated that they were unable to isolate Cage-GID in vitro. It is thus unclear to the reviewer that whether this conformation is just an artifact.

To address this question, we reanalyzed our cryo-EM data obtained for endogenous GID purified from yeast grown under carbon starvation (Sherpa, Chrustowicz et al., 2021), which yielded a 3D reconstruction corresponding to Cage-GID. This is now included as Supplementary Figs. 6a-c in the revised manuscript.

3) In Fig. 2a and b, it appears that Gid12 also consists of several helixes in addition to the beta-propeller. But it is not discussed in the text.

In the revision, we now briefly discuss the helical embellishments to the β-propeller, and show these in more detail in the extended data figures.

4) Blades numbers should be labeled in Fig. 2b.

We numbered the blades in the figure in the revised manuscript.

5) Fig. 5c is confusing. Can it be shown in a 3D representation as in Fig. 5a and b?

We thank the reviewer for pointing this out, and deleted the confusing figure in the revision.

6) Fig. 7c. Why the time course for Fbp1 and Mdh2 are from 0-2 hr, while it is 0-4 hr for Ic1 and Pck1? It should be consistent for every substrate.

The GID E3 gluconeogenic enzyme substrates are degraded over different time-courses after glucose replenishment. We therefore monitor each enzyme over the time-course during which its level declines.

Reviewer #2 (Remarks to the Author):

Dear Editors of Nature Communication,

The manuscript "Cryo-EM structures of Gid12-bound GID E3 reveal steric blockade as a mechanism inhibiting substrate ubiquitylation" describes the function of the protein Ipf1 (YDL176W) as a potential regulator of GID-E3 ligase activity. Ipf1 (in the following publication named Gid12) was previously shown to interact with different subunits of the Gid-complex [1; 2; 3]. The Gid-complex is an evolutionary conserved ubiquitin-ligase complex that targets key enzymes of gluconeogenesis for glucose induced degradation in yeast and exists with different subunit compositions. Gid4 is one of several substrate recognition factors that bind yeast substrates via their N-terminal proline residues to allow polyubiquitination and subsequent proteasomal degradation. Gid4 levels strongly vary depending on the carbon source, thus allowing substrate degradation depending on Gid4 levels. Gid4 is almost absent in cells grown on YPEthanol (ethanol as C-source) and is strongly upregulated on YPD (glucose as C-source), allowing substrate degradation depending on Gid4 levels [4; 5]. Other layers of GID-complex regulation are currently unknown.

The current study from the Schulman lab uses co-immunoprecipitation methods to isolate endogenous Ipf1 from cell lysates under glycolytic conditions. The authors find the core Gid-

complex subunits enriched in the precipitate, confirming previous data that Ipf1 binds Gid-complex subunits. Co-expression of Gid12 with other Gid-subunits and subsequent Cryo-EM, allows insights into structural composition and suggests direct binding to the subunits Gid4 and Gid5 within the complex. Additionally, the structural data indicates that Gid12 binding induces conformational changes of loop structures at the entrance of the substrate binding tunnel within Gid4 and partially occupies space between Gid4 and the Gid2/9 RING domains. The authors suggest that substrate accessibility could be compromised by Gid12 binding. Additionally, the authors describe a previously unrecognized 5 MDa Gid-complex assembly that seems to consists of three Chelator-GidSR assemblies that was named the Cage-GIDSR4 assembly. This complex assembles with or without Gid12 in vitro. Biochemical assays suggest, that Gid12 blocks Gid4 assembly into the anticipatory Gid-complex and inhibits polyubiquitination of Fbp1, Mdh2 and Icl1 in vitro. In vivo, overexpression of Gid12 results in stabilization of gluconeogenic key enzymes upon glucose replenishment. Gid12 is abundant when cells are grown on YPD, on YPE its levels drop and increase upon glucose recovery. The authors immunoprecipitate Gid4 from lysates generated from yeast cells grown on different carbon sources and find Gid12 co-precipitating when cells are grown on YPD and on YPE but not after glucose recovery.

In summary, the manuscript provides interesting novel insights into the function of Ipf1 in the regulation of Gid-complex activity. However, more detailed information of how Gid12 regulation works is required. Also at this stage it remains completely unclear if the CAGE-assembly described in the manuscript exists in vivo and which function it may have.The following points should be addressed prior to publication.

We thank the reviewer for such kind comments! We are very pleased by the enthusiastic response!

Major concerns:
1. In Figure 5 the authors describe a 5 MDa Gid-complex assembly that they name the Cage-GID complex. Unfortunately, the authors neither provide in vivo data to convince the reader of its existence in cells, nor do they provide experimental data of a possible function. Furthermore, the authors show that the assembly is independent of Gid12. The focus of the manuscript is put on the function of Gid12, in order to keep the focus I recommend to remove the entire Figure 5 from the manuscript. A minimal requirement is to purify the complex from lysates and to determine the molecular mass of the different sub-compositions by gel chromatography or glycerol gradient centrifugation experiments.

To address this question, we reanalyzed our cryo-EM data obtained for endogenous GID purified from yeast grown under carbon starvation (Sherpa, Chrustowicz et al., 2021), which yielded a 3D reconstruction corresponding to Cage-GID Å resolution. This is now included as Supplementary Figs. 6a-c in the revised manuscript.

2. The authors convincingly show that Ipf1/Gid12 binds to the Gid-complex and suggest that it prevents Gid4 incorporation into the complex, and thus substrate polyubiquitination and degradation. The data is consistent and supports such theory. However, it remains unclear how Gid12 is released from the complex and if Gid12 functions for other substrate recognition factors as well.

We apologize for lack of clarity, but our data suggest that Gid12 binds Gid4, seals Gid4 onto GID E3, reshapes the degron-binding site in Gid4 in such a way that reduces intrinsic recognition of the Mdh2 degron and potentiates recognition of that from Fbp1, and projects into the region substrate occupies to access the ubiquitylation active sites.

Data indicating that Gid12 stably binds versions of the GID complex that contain Gid4 include:

1) Fig. 1b and supplementary Fig. 10c show that Gid12 binding to the recombinant GID complex was substantially reduced upon exclusion of Gid4.

2) Proteomic analysis of the GID complex immunoprecipitated from yeast lysates (by virtue of Gid8 3xHA tagged at the endogenous locus) in WT versus *gid4* deletion strains shows loss endogenous Gid12 association upon loss of Gid4 (Supplementary Fig. 1b).

3) The structure shows Gid12 substantially binds to Gid4 in GID E3 complexes.

4) Our substrate receptor exchange assay in Fig. 6a suggests that Gid12 seals Gid4 onto the GID complex and prevents Gid4 dissociation.

At this point, we do not know how Gid12 is released from the complex. Given that we could only produce Gid12 in complex with Gid4, we speculate that rather than dissociation, Gid12 may be subjected to degradation along with Gid4. Importantly, Gid12 becomes substoichiometric to Gid4 over time after the switch from gluconeogenic to glycolytic conditions. So new production of excess Gid4 could allow formation of active GID E3 ligases. We added this concept to the discussion of the revised manuscript.

Gid4 is highly homologous to Gid10 (Supplementary Fig. 10a, b). Moreover, from our pulldown experiments, we see that Gid12 can form complex with Gid10 (Figure 10c), so although we do not detect Gid10 in the Gid12 immunoprecipitates, future studies may discover that Gid12 can function with substrate recognition factors in addition to Gid4.

Questions arrising:
- Is Ipf1 a general factor that blocks docking of substrate recognition factors like Gid10 and Gid11? Does overexpression of GID12 prevent the incorporation of Gid10 into the complex and does Gid12 overexpression block degradation of e.g. Phm8 as well?

We performed quantitative total proteome analyses with wild type yeast, a *gid12* deletion strain, and a strain overexpressing Gid12 under glycolytic conditions and various times after replenishing glucose. However, Gid10 was not identified in any conditions in the total proteome experiments and Gid11(YLR149C) was identified only in *gid12* deletion strains (Supplementary Fig.8c). We compared the levels of all reported substrates, including those depending on Gid10 and Gid11. While impact of *gid12* deletion is subtle, overexpression of Gid12 stabilizes all known Gid4-dependent substrates Fbp1, Mdh2, Icl1 and Pck1), and one additional substrate (Aro10). Gid12 overexpression did not These new data are reported in Supplementary Figs. 8b and 9.

We also attempted to test if Gid12 could bind recombinant GID complexes containing either Gid10 or Gid11. Although we were not able to express Gid11, we did observe Gid12 binding to the substrate receptor-scaffolding subcomplex containing Gid10 (Gid1-Gid8-Gid5-Gid10). These results are shown in Supplementary Fig.10c.

- Gid4 is a substrate recognition factor but also a substrate of the Gid-complex. Upon glucose replenishment Gid4 gets first upregulated and then degraded probably together with the substrate. Is Gid4 degradation also affected by Gid12 overexpression?

We performed new experiments to address this question. The answer is yes, Gid4 levels increase in yeast overexpressing Gid12 (Fig. 7b, Supplementary Fig. 9).

- Gid12 levels differ dependent on the growth conditions (Figure 7a). Cells grown on YPD and 1 h after glucose supplementation display high levels of Gid12, while grown on non-fermentable carbon sources Gid12 is present at low levels. How is the level of Gid12

regulated? Is there transcriptional regulation? Is Gid12 turned over depending on the carbon source and might this be a way to activate the complex?

At this point, how the levels of Gid12 are regulated remains unknown. We feel our current work will prompt future studies to address these questions.

- Figure 1B and Figure 2 suggest that Gid12 primarily associates with the Gid-complex via the subunits Gid4 and Gid5. To test whether this is also the case in vivo, Co-IPs should be performed with a tagged subunit of the Gid-complex and Gid12-6*HA in WT and in gid4 deletion strains.

We performed the experiment, immunoprecipitating with a core subunit (Gid8) tagged at the endogenous locus. Indeed, the interaction with Gid12 is reduced in the *gid4* deletion strain. These data are shown in Revised Supplementary Fig. 1b.

- In Figure 7b the authors precipitate Gid4 and quantify Co-IPed Gid2 and Gid12. Surprisingly, after Co-IP the level of Gid4 and Gid12 is similar to the level of Gid2 when grown on YPE. This suggests, that the entire remaining low levels of Gid4 and Gid12 under these conditions are trapped in the complex in an inactive state. It appears that Gid12 not only prevents unbound Gid4 from associating with the GidAnt, but also traps remaining Gid4 within the primed but inactivated complex. Glucose induced removal of Gid12 from the primed inactive complex would guarantee a rapid first response to glucose supplementation. To better assess this possibility a Gid-complex subunit like Gid1 would be better suited for pull down experiments because the protein level does not fluctuate dependent on growth conditions (like for Gid4). Most of the Gid4 protein pulled down in Fig7b is protein not bound to the complex.

Unfortunately, the low levels of Gid4 and Gid12 present under these conditions precluded sufficient detection above background in IPs with core subunits under most conditions.

We do appreciate this suggestion, and have added the following speculation to the Discussion: “It seems that after the switch from gluconeogenesis to glycolytic conditions, the production of new Gid4 in substantial excess over Gid12 levels would allow formation of active GID E3 ligases. Future studies will be required to determine whether additional forms of regulation, for example Gid12 degradation, further contribute to rapid activation of glucose-induced GID E3 ligase activity.”

Minor points:

We appreciate all the minor suggestions below and we have changed all of them in the main text and also cited all the additional papers.

1. Please use either ubiquitylation or ubiquitination. P.30 Figure legend 6c change ubiquitination to ubiquitylation.
2. Throughout the whole manuscript the term carbon starvation is used. I find the term misleading because ethanol is also a C-source. Please change the term e.g. use the term non-fermentable carbon source or glucose retraction
3. P9 line9: change the sentence “Use of a tagged Gid4 version therefore allows to distinguish between Gid4 assembled into…and Gid4 free in solution.
4. P10 heading. …stabilizes Fbp1 protein in vivo (not the levels)
5. P10 line 16: The sentence ‘ this is consistent with prior…. I don’t understand why a tag is consistent with protein levels, please explain
6. P10 line 20 change carbon stress to growth on a non-fermentable carbon source
7. P10 line 23 is restored should be was restored
8. P10 line 25 delete , as might be expected for a modulatory protein

9. P10 line 26 insert '…Gid12 in complex with Gid4 by co-immunoprecipitation
10. P11 line 12 …had the capacity to inhibit…
11. P12 line 6 towards FBP1
12. P12 line13 by the effect
13. P12 line 20 … Gid12 may inhibit_ GID E3….
14. P13 line 2 delete enzyme TCA cycle enzymes.
15. P14 …by centrifugation at 20000 rpm …please mention rotor or use g instead
16. P17 might be my progressing cataract but some sections appear grey…please change to black
17. P23 in the acknowledgement delete one of the two 'for peptide synthesis'
18. Please cite Liu et al., 2020 on P 3 line 16

[1] N.J. Krogan, G. Cagney, H. Yu, G. Zhong, X. Guo, A. Ignatchenko, J. Li, S. Pu, N. Datta, A.P. Tikuisis, T. Punna, J.M. Peregrin-Alvarez, M. Shales, X. Zhang, M. Davey, M.D. Robinson, A. Paccanaro, J.E. Bray, A. Sheung, B. Beattie, D.P. Richards, V. Canadien, A. Lalev, F. Mena, P. Wong, A. Starostine, M.M. Canete, J. Vlasblom, S. Wu, C. Orsi, S.R. Collins, S. Chandran, R. Haw, J.J. Rilstone, K. Gandi, N.J. Thompson, G. Musso, P. St Onge, S. Ghanny, M.H. Lam, G. Butland, A.M. Altaf-Ul, S. Kanaya, A. Shilatifard, E. O'Shea, J.S. Weissman, C.J. Ingles, T.R. Hughes, J. Parkinson, M. Gerstein, S.J. Wodak, A. Emili, and J.F. Greenblatt, Global landscape of protein complexes in the yeast Saccharomyces cerevisiae. Nature 440 (2006) 637-43.
[2] S. Pitre, F. Dehne, A. Chan, J. Cheetham, A. Duong, A. Emili, M. Gebbia, J. Greenblatt, M. Jessulat, N. Krogan, X. Luo, and A. Golshani, PIPE: a protein-protein interaction prediction engine based on the re-occurring short polypeptide sequences between known interacting protein pairs. BMC Bioinformatics 7 (2006) 365.
[3] R.I. Subbotin, and B.T. Chait, A pipeline for determining protein-protein interactions and proximities in the cellular milieu. Mol Cell Proteomics 13 (2014) 2824-35.
[4] O. Santt, T. Pfirrmann, B. Braun, J. Juretschke, P. Kimmig, H. Scheel, K. Hofmann, M. Thumm, and D.H. Wolf, The yeast GID complex, a novel ubiquitin ligase (E3) involved in the regulation of carbohydrate metabolism. Mol Biol Cell 19 (2008) 3323-33.
[5] R. Menssen, K. Bui, and D.H. Wolf, Regulation of the Gid ubiquitin ligase recognition subunit Gid4. FEBS Lett 592 (2018) 3286-3294.

Reviewer #3 (Remarks to the Author):

This paper describes the investigation of the role of the Gid12 protein in the regulation of the multi-subinit GID E3 ligase system primarily by cryo-EM based structural analysis of several GID protein assemblies. In particular, the authors report a high resolution structure of a 5 subunit Gid12- SRS complex and intermediate resolution structures of larger structures referred to as Gid12-GIDSR4, Gid12-Chelator-GIDSR4 and Gid12-Cage-GIDSR4. The first three structures build on recent previous structural analysis by the Schulman group of equivalent structures lacking the Gid12 protein, while the fourth represents a novel higher order complex containing three copies of Gid12-Chelator-GIDSR4. GID E3 ligases are interesting and relevant systems which have been substantially characterised in recent studies to which the Schulman group has substantially contributed. The structural analysis and biochemical measurements presented here indicate that Gid12 acts by sterically interfering with ability of GID E3 ligase complexes to engage productively with its Fbp1 substrate. Overall I consider that this is a well conducted and valuable piece of work which contributes to a greater understanding of the Gid12 protein, the role of which, nevertheless, remains somewhat nebulous.

We are very pleased by the Reviewer's enthusiasm for our study. Thank you for your kind comments!

Detailed points:
1) The paper describes a range of GID complexes characterised by different combinations of subunits and symmetries with a range of different names made up of acronyms. For readers less familiar with the GID story I think it might help to provide a guide perhaps in the form of a table or set of cartoons to make it easier to keep track of what is going on.

We added a Supplementary Table 3, which includes the names of the all the GID complexes and subcomplexes, the respective subunits and their stoichiometries.

2) Aside from the Gid12- SRS complex, the cryo-EM analysis of other larger Gid complexes described in this paper are of somewhat limited resolution. This is most likely a consequence of either compositional or conformational heterogeneity (or both). It would be interesting to learn to what extent this could be compensated for by local or multi body refinement or in the case of the symmetric complexes by symmetry expansion.

We collected a Titan Krios dataset with in vitro purified Cage-GID together with PHSVTP-Fbp1 (Fbp1 with its N-terminal degron -PTLVNG exchanged for Mdh2 degron – PHSVTP and we were able to improve the resolution by symmetry expansion to 12 A. These data are shown in Supplementary Figure 6.

REVIEWERS' COMMENTS

Reviewer #1 (Remarks to the Author):

The manuscript by Qiao et al has been substantially revised in response to the previous round of reviews. The authors have satisfied my major concerns with both clarification of the text and additional experiments. This is a very interesting body of work that will make a strong contribution to yeast biology and ubiquitination field.

Reviewer #2 (Remarks to the Author):

All my suggestions and concerns were adressed and answered adequately. The manuscript is ready for publication.

Reviewer comments and point-by-point responses in blue:

Reviewer #1 (Remarks to the Author):

The manuscript by Qiao et al has been substantially revised in response to the previous round of reviews. The authors have satisfied my major concerns with both clarification of the text and additional experiments. This is a very interesting body of work that will make a strong contribution to yeast biology and ubiquitination field.

We thank the reviewer for such kind comments! We are very pleased by the enthusiastic response!

Reviewer #2 (Remarks to the Author):

All my suggestions and concerns were addressed and answered adequately. The manuscript is ready for publication.

We thank the reviewer for such kind comments! We are very pleased by the enthusiastic response!
